# Supplementary material for: Analysis of categorical data from biological experiments with logistic regression and CMH tests
Source: PLoS One. 2025 Nov 17;20(11):e0335143. doi: 10.1371/journal.pone.0335143 (PMC12622779; doi:10.1371/journal.pone.0335143)
Supplement: S1 Table — A complete list of strains used in this study. (PDF) [file pone.0335143.s001.pdf]

Supplemental Table 1

**Strain Name**	**Genotype**	**Reference**
N2	wild type	Caenorhabditis Genetics Center
ZB4065	*bzIs166[pmec-4::mCherry]*	Melentijevic et al. 2017
ZG31	*hif-1(ia4)*	Jiang et al 2001
JT307	*egl-9(sa307)*	Trent et al 1983
