# Supplementary material for: Analysis of categorical data from biological experiments with logistic regression and CMH tests
Source: PLoS One. 2025 Nov 17;20(11):e0335143. doi: 10.1371/journal.pone.0335143 (PMC12622779; doi:10.1371/journal.pone.0335143)
Supplement: S1 File — Detailed explanation of the provided code to reformat data for analysis, run CMH and logistic regression analyses, and plot these results in R. (PDF) [file pone.0335143.s002.pdf]

Analysis of Categorical Data

Forked from untitled protocol

Rebecca Androwski[1], Tatiana Popovitchenko[2], Joelle Smart[2], Sho Ogino[2], Guoqiang Wang[1], Mark Saba[1], Christopher Rongo[2], Monica Driscoll[1], Jason Roy[3]

[1]Department of Molecular Biology and Biochemistry, Nelson Biological Laboratories, Rutgers, The State University of New Jersey, Piscataway, New Jersey 08854, USA.;
[2]Department of Genetics, Waksman Institute, Rutgers, The State University of New Jersey, Piscataway, New Jersey 08854, USA.;
[3]Department of Biostatistics and Epidemiology, Rutgers School of Public Health, Piscataway, New Jersey 08854, USA.

Rebecca Androwski
Rutgers University

Protocol Info: Rebecca Androwski, Tatiana Popovitchenko, Joelle Smart, Sho Ogino, Guoqiang Wang, Mark Saba, Christopher Rongo, Monica Driscoll, Jason Roy . Analysis of Categorical Data. **protocols.io** **https://protocols.io/view/analysis-of-categorical-data-d74j9qun**

Created: April 16, 2025

Last Modified: September 13, 2025

Protocol Integer ID: 126827

Keywords: Biostatistics, R programming, Cochran–Mantel–Haenszel, Tutorial, C. elegans, Behavioral assays, Generalized linear models (GLM), Data hygiene

Funders Acknowledgements:
RA
Grant ID: NIH 5T32NS115700-04
TP
Grant ID: NIH 5T32NS115700-04
CR
Grant ID: NIH R01GM101972
MD
Grant ID: NIH R01AG047101

Abstract

The choice of appropriate statistical tests in experimental biology is critical for scientific rigor and can be challenging in the case of categorical data analysis. Using example datasets from *Caenorhabditis elegans* research, we conduct statistical analysis of (1) a rare cellular event involving the formation of a neuronal extrusion called an exopher and (2) a variable behavioral response across time. We employ the Cochran–Mantel–Haenszel (CMH) test and logistic regression for analysis. Recognizing potential accessibility issues using logistic regression, we provide step-by-step tutorials and example code. We emphasize that logistic regression can handle both simple and complex multivariable datasets; logistic regression can also provide more comprehensive insights into experimental outcomes when compared to simpler tests like CMH. By analyzing real biological examples and demonstrating their analysis with R code, we provide a practical guide for biologists to enhance the rigor and reproducibility of categorical data analysis in experimental studies.

Materials

Active internet connection to download packages in R

Computer with operating system compatible with a recent version of R and R Studio

A working keyboard

Software Loading

1 Download RStudio for your OS at: https://posit.co/downloads/

This software is known as an integrated development environment (IDE), and it is away to make coding more visual. The alternative is to open terminal (a command line interface) and code there.

2 Install R following the instructions at: **https://cran.r-project.org/**

Setting up the coding environment

3 Fig1. Open R studio. You will see a window that looks like the following:

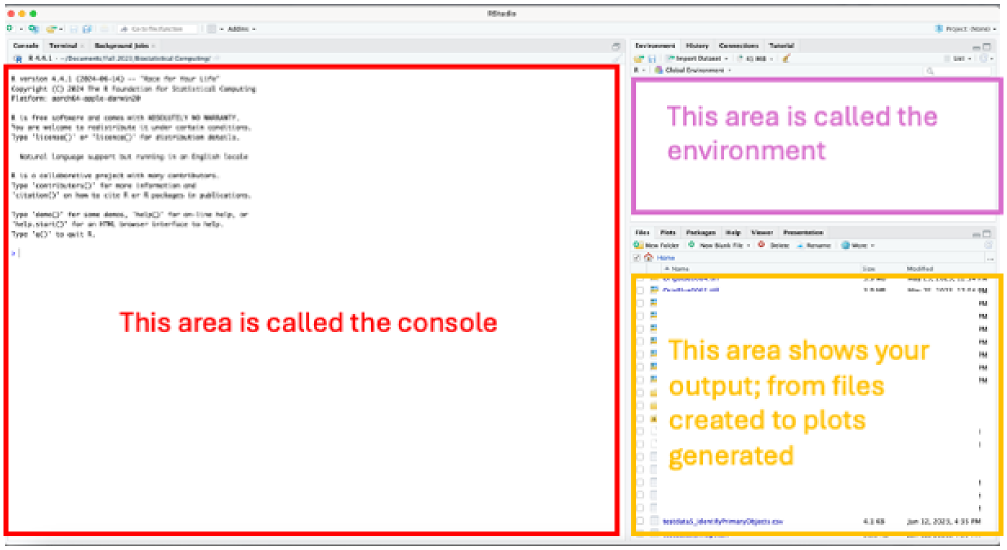

Fig1. This is the R studio initial window.

4 Open the project file (highlighted in pink belowFig2. Finder window)

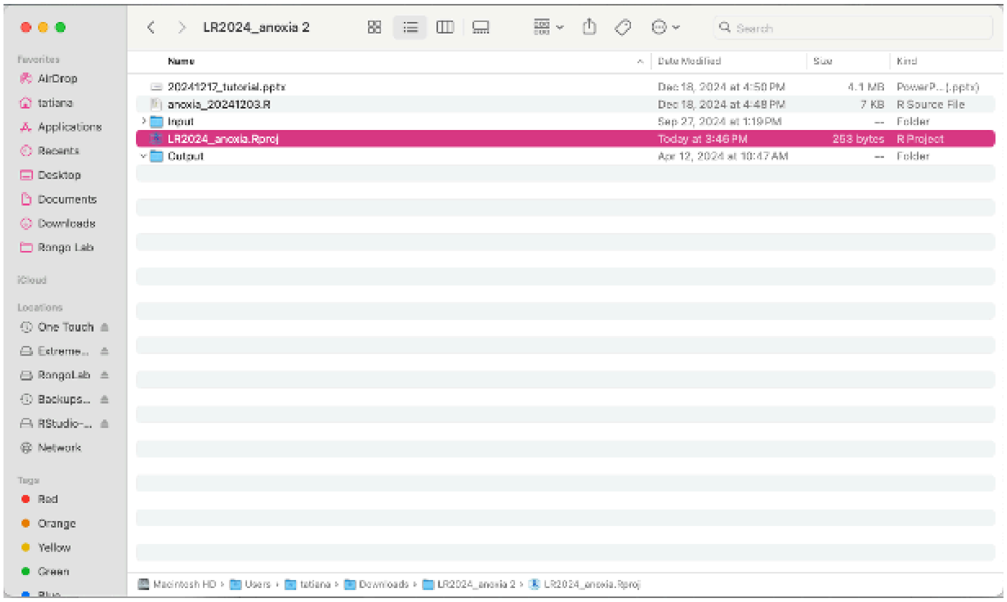

Fig2. Finder window

5 Open the R file in the output box, see yellow outlined box and the inset.

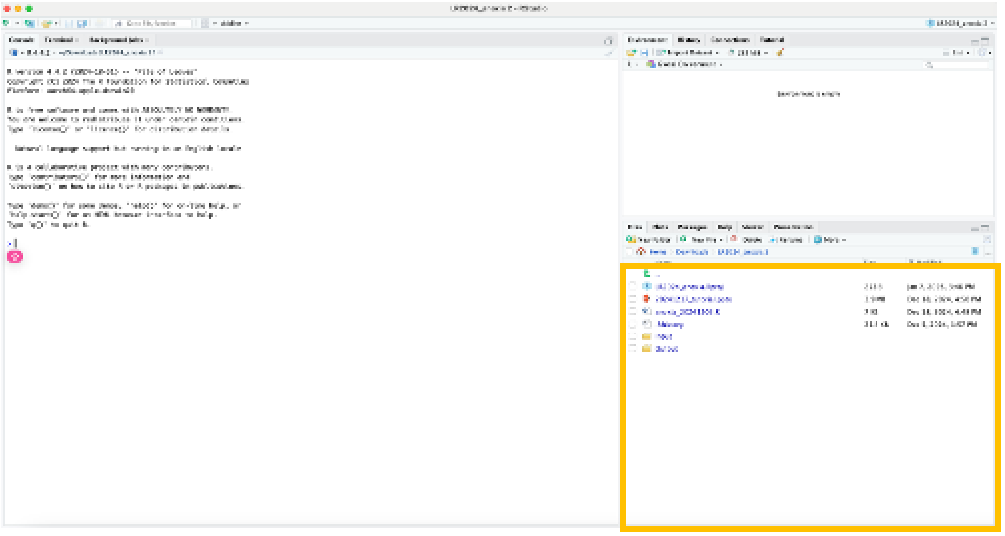

Fig3. Here is the R Studio window.

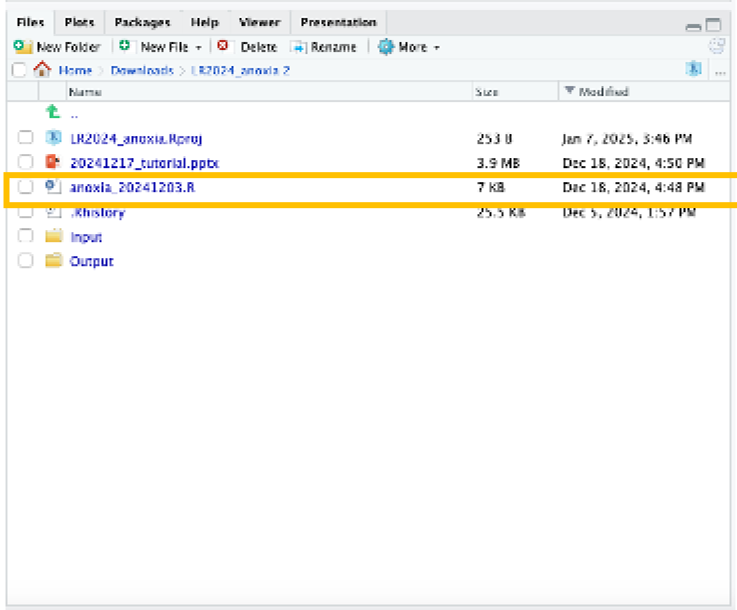

Fig4. Here is a zoomed-in view of the yellow boxed area in Fig3. Open the file outlined in yellow.

Your new R Studio window will look like the following, notice the addition of a new section called the console:

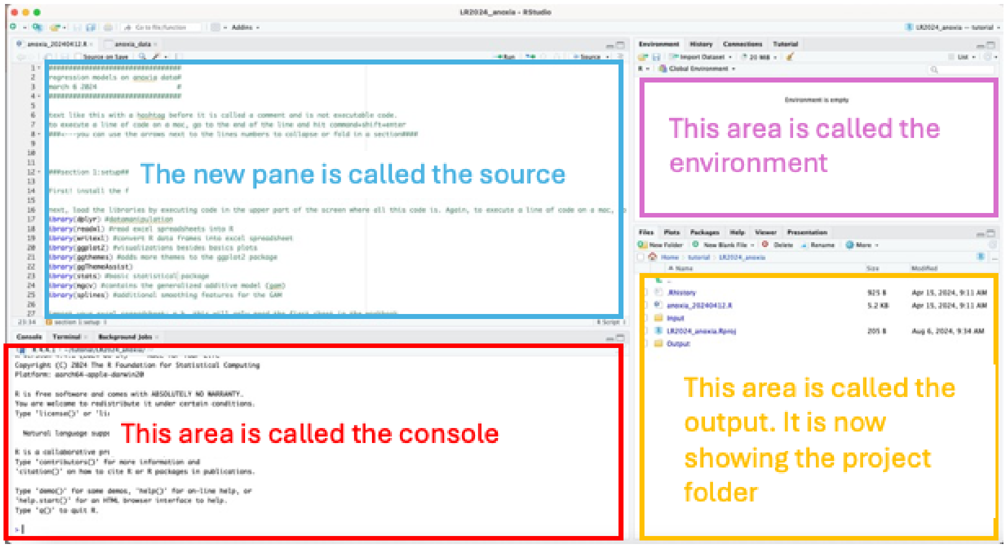

Fig5. The R studio window after opening a source file now has a console section.

You will notice that the source is already full of code if you opened the project file. You will be going through the code line by line to generate the regression models and the graphs in this project.

Each step will refer to line numbers, found here:

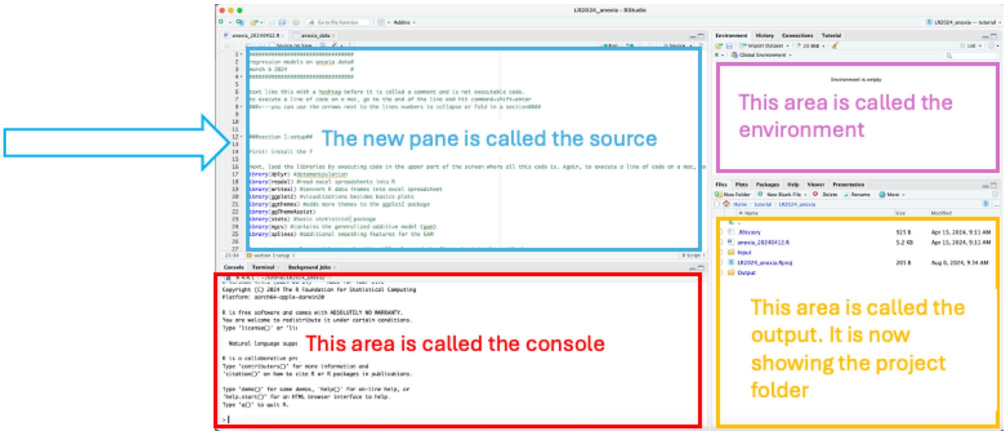

Fig6. An arrow indicating where the line numbers to the code are.

Getting started coding

6 lines 14-15:

Install the packages by pasting this line of code into the console, and hit enter:

```
install.packages(c('dplyr', 'readxl', 'writexl', 'ggplot2',
'ggthemes', 'ggThemeAssist', 'stats', 'mgcv', 'splines’,
‘ggprism’))
```

This step requires an active internet connection.

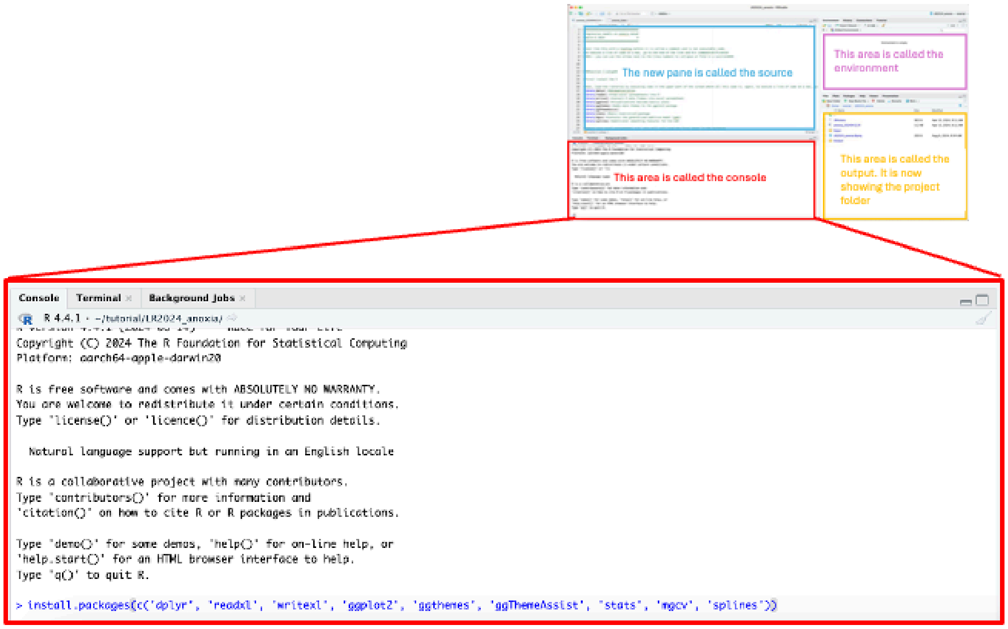

Fig7. Zoomed in view of console where packages need to be installed.

[More information on packages here.]

7 lines 17-28:

After installing packages, you now have access to the libraries contained within those packages. Load the libraries by highlighting lines 17-28 in the source and hit command+enter on your keyboard to execute:

```
#next, load the libraries by executing code in the upper part of
the screen where all this code is. Again, to execute a line of
code on a mac, go to the end of the line and hit
command+shift+enter
library(dplyr) #datamanipulation
library(readxl) #read excel spreadsheets into R
library(writexl) #convert R data frames into excel spreadsheet
library(ggplot2) #visualizations besides basics plots
library(ggthemes) #adds more themes to the ggplot2 package
library(ggprism) #makes your graphs look like ones generated in
prism
library(ggsignif) #To add significance stars to your ggplot2 graph
library(forcats) #To reorder the data
library(stats) #basic statistical package
library(mgcv) #contains the generalized additive model (gam)
library(splines) #additional smoothing features for the GAM
```

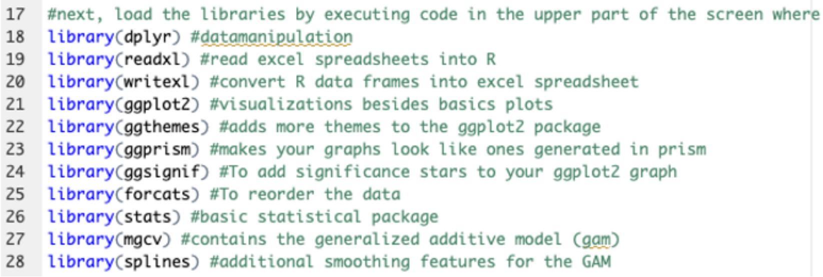

```
17 #next, load the libraries by executing code in the upper part of the screen where all this code is.
18 library(dplyr) #datamanipulation
19 library(readxl) #read excel spreadsheets into R
20 library(writexl) #convert R data frames into excel spreadsheet
21 library(ggplot2) #visualizations besides basics plots
22 library(ggthemes) #adds more themes to the ggplot2 package
23 library(ggprism) #makes your graphs look like ones generated in prism
24 library(ggsignif) #To add significance stars to your ggplot2 graph
25 library(forcats) #To reorder the data
26 library(stats) #basic statistical package
27 library(mgcv) #contains the generalized additive model (gam)
28 library(splines) #additional smoothing features for the GAM
```

Fig8. Showing which lines to highlight in the code to install the libraries needed to carry out the code.

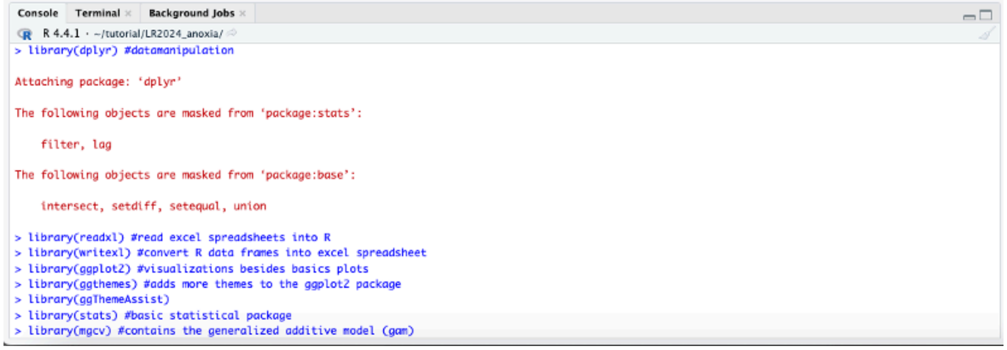


```
> library(dplyr) #datamanipulation

Attaching package: ‘dplyr’

The following objects are masked from ‘package:stats’:

    filter, lag

The following objects are masked from ‘package:base’:

    intersect, setdiff, setequal, union

> library(readxl) #read excel spreadsheets into R
> library(writexl) #convert R data frames into excel spreadsheet
> library(ggplot2) #visualizations besides basics plots
> library(ggthemes) #adds more themes to the ggplot2 package
> library(ggThemeAssist)
> library(stats) #basic statistical package
> library(mgcv) #contains the generalized additive model (gam)
```

Fig9. The command in the source that you just executed will be shown as a task having been done in the console.

8 lines 30-31:

Import your data. The project data is in the form of an excel spreadsheet (called “an_final10.xlsx”). If your data is in another kind of data table, you will search for the correct command to import that specific file format. This command (“read_excel”) imports excel spreadsheets into R.

```
#import your excel spreadsheet; nota bene (n.b.) this will only
read the first sheet in the workbook
anoxia_data <- read_excel("input/data_final.xlsx")
```

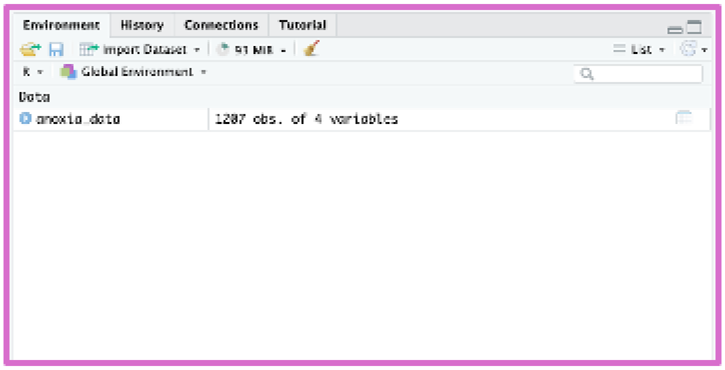

Fig10. Notice that when you execute line 31, the new object you created (the R version of your excel spreadsheet) appears in your environment pane. If you click on that object in your environment pane, it will open in a new window in the R studio program next to your source code.

Let's take a second to dissect the anatomy of a command in R.

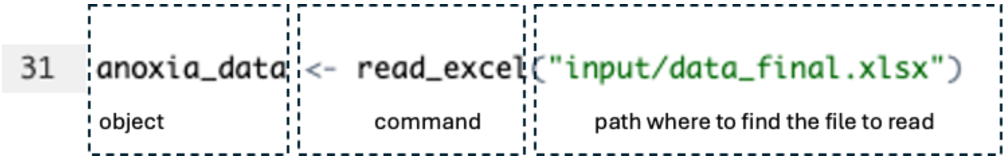

Fig11. In this case, I am telling R to look in the folder called input for a file called data_final.xlsx. You
will notice that in the .zip file you downloaded (Logistic_Regression_Anoxia.zip), there is the project file, the R code, and two folders called input and output. Opening up a project tells R you will work in the folder the project is located. If you wanted to import a file not in this folder, then just write out the full path of the file.

More information on figuring out a path: [Mac](#) [PC](#) [Linux](#)

9 We are now done with section 1, let's collapse Section 1 code by clicking on the arrow next to line number 12. This is not necessary, just aesthetically organized.

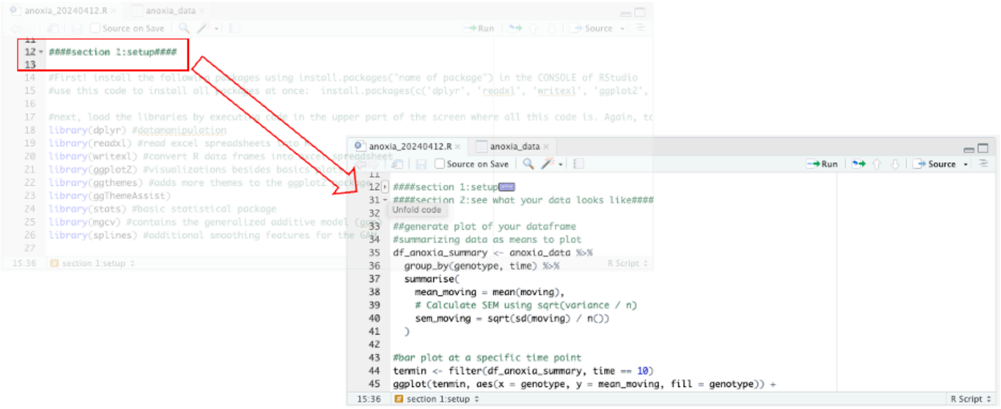

Fig12. Collapsing section 1 in R Studio

You can also navigate sections by clicking through the list here:

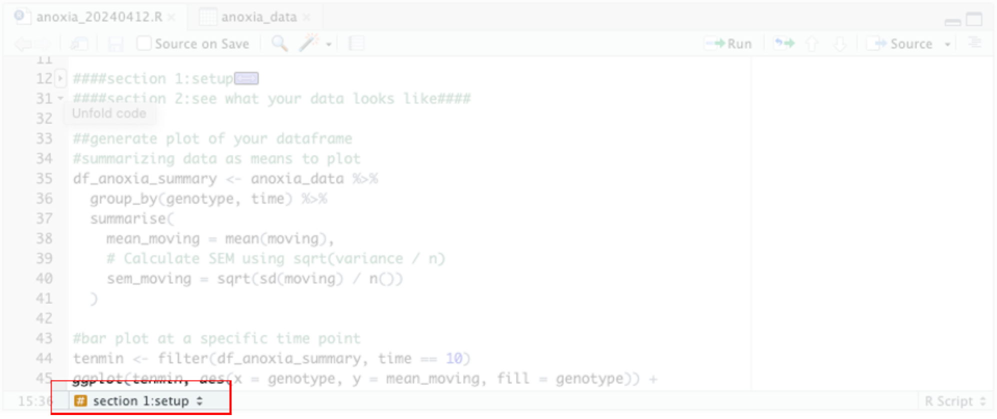

Fig13. Alternative to navigating through sections of code.

Data Visualization

10 This section will go into tools in R that allow you to graph your data. Executing this tutorial as written should be straightforward, but as scientists, sometimes we get stuck on little details of the graphing or aesthetics. If this happens to you- don't fret! It's normal.

Feel free to skip this section if you are here to learn about the statistical output in R.

11 lines 35-40:

These lines of code generate our first visualization using ggplot.

```
#Plot all trials separately
ggplot(anoxia_data, aes(x = genotype, y = moving, fill =
genotype)) +
  stat_summary(fun = "mean", geom = "bar") +
  stat_summary(fun.data = "mean_se", geom = "errorbar", width =
0.2) +
  theme_prism()+
  facet_wrap(~ trial)  # Create separate plots for each time point
```

This code tells R to go into the anoxia_data object we created in the previous section and create a plot from that dataset. We are making a simple bar plot (geom= "bar") of the means of each genotype (x axis in the "aes" function on line 36) in the moving column (y axis in the same line of code). I am interested in looking at these means in each trial (facet_wrap(~trial) in line 40), as this experiment is variable and not all trials worked (egl-9 must be > than N2)

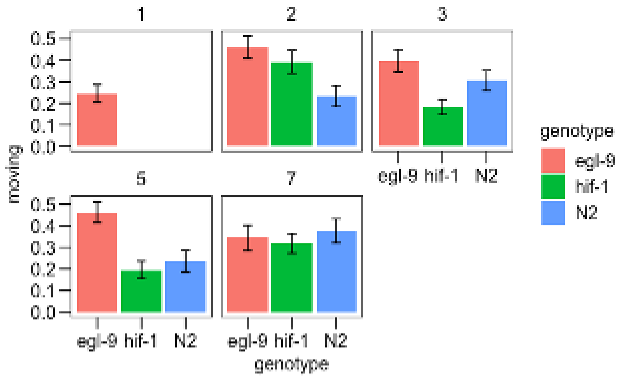

Fig 14. Facet wrap plot from ggplots

The format of the ggplot code is very stereotyped. It is a good idea to get sample code to start with and then customize from there.

We call on the ggplot function to plot our anoxia_data as a bar plot (geom = "bar"). ggplot (actually, ggplot2 is the current version) has many different visualization options. This is not the only way to create graphs within R. But it is a very versatile option; we can customize axes names, chart line widths, and use a vast variety of themes to give our charts a nice look.

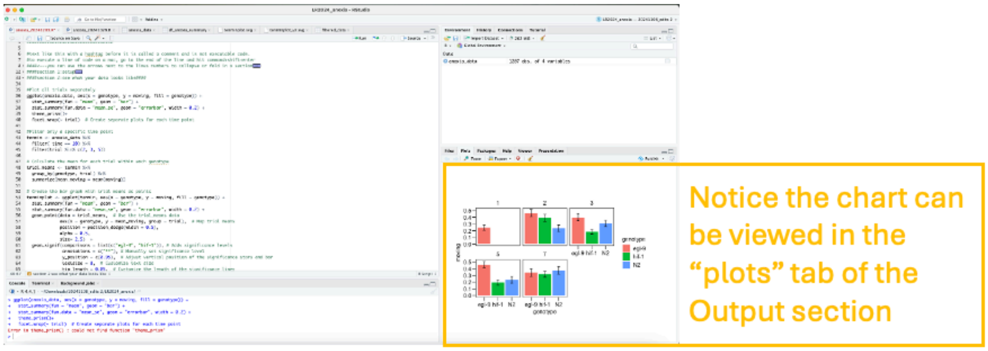

Fig15. Viewing your plot in Rstudio

Please do browse more **information on ggplot2**. We won't go into what each line of code here means, but this information is easily accessible.

12 One basic point in the code is you can continue to add customizations to the graph by adding a plus sign and then the desired lines of code. This is notable most R code uses the pipe operator (%>%) to group multiple operations on a single dataset.

Let's create a simple bar graph of a single time point in our dataset.

```
# Create the bar graph with trial means as points
tenminplot <- ggplot(tenmin, aes(x = genotype, y = moving, fill =
genotype)) +
  stat_summary(fun = "mean", geom = "bar") +
  stat_summary(fun.data = "mean_se", geom = "errorbar", width =
0.2) +
  geom_point(data = trial_means,  # Use the trial_means data
             aes(x = genotype, y = mean_moving, group = trial),  #
Map trial means
             position = position_dodge(width = 0.5),
             alpha = 0.5,
             size= 2.5)  +
  geom_signif(comparisons = list(c("egl-9", "hif-1")), # Adds
significance levels
              annotations = c("**"), # Manually set significance
level
              y_position = c(0.95),  # Adjust vertical position of
the significance stars and bar
              textsize = 8,  # Customize text size
              tip_length = 0.05,  # Customize the length of the
significance lines
              vjust = 0.5) +  # Adjust vertical position of stars
in relation to bar
  geom_signif(comparisons = list(c("egl-9", "N2")), # Adds
significance levels
              annotations = c("**"),  # Manually set significance
level
              y_position = c(1.1),  # Adjust vertical position of
the significance stars and bar
              textsize = 8,  # Customize text size
              tip_length = 0.05,  # Customize the length of the
significance lines
              vjust = 0.5) +  # Adjust vertical position of stars
in relation to bar
  labs(title = "Movement at 10 Minutes",
       x = "Genotype",
       y = "Mean Movement") +
  scale_y_continuous(expand = expansion(mult = c(0.00, 0.1)))+
#moves 0 at y-axis to intersect the x-axis
  theme_prism()+
  theme(
    axis.text.x = element_text(size = 14, angle = 45, vjust = 1,
hjust = 1, face = "bold.italic"),
    axis.text.y = element_text(size = 14, face = "bold"),
    axis.title.x = element_text(size = 16, face = "bold", vjust =
-3),
```

```
    axis.title.y = element_text(size = 16, face = "bold"),
    plot.title = element_text(size = 20, face = "bold"),
    legend.position = "none")  # Removes the legend
```

```
52 # Create the bar graph with trial means as points
53 ggplot(tenmin, aes(x = genotype, y = moving, fill = genotype)) +
54   stat_summary(fun = "mean", geom = "bar") +
55   stat_summary(fun.data = "mean_se", geom = "errorbar", width = 0.2) +
56   geom_point(data = trial_means,  # Use the trial_means data
57              aes(x = genotype, y = mean_moving, group = trial),  # Map trial means
58              position = position_dodge(width = 0.5),
59              alpha = 0.5,
60              size= 2.5)  +
61   geom_signif(comparisons = list(c("egl-9", "hif-1")), # Adds significance levels
62               annotations = c("**"), # Manually set significance level
63               y_position = c(0.95),  # Adjust vertical position of the significance stars and bar
64               textsize = 8,  # Customize text size
65               tip_length = 0.05,  # Customize the length of the significance lines
66               vjust = 0.5) +  # Adjust vertical position of stars in relation to bar
67   geom_signif(comparisons = list(c("egl-9", "N2")), # Adds significance levels
68               annotations = c("**"),  # Manually set significance level
69               y_position = c(1.1),  # Adjust vertical position of the significance stars and bar
70               textsize = 8,  # Customize text size
71               tip_length = 0.05,  # Customize the length of the significance lines
72               vjust = 0.5) +  # Adjust vertical position of stars in relation to bar
73   labs(title = "Movement at 10 Minutes",
74        x = "Genotype",
75        y = "Mean Movement") +
76   scale_y_continuous(expand = expansion(mult = c(0.00, 0.15)))+ #moves 0 at y-axis to intersect the x-axis
77   theme_prism()+
78   theme(
79     axis.text.x = element_text(size = 14, angle = 45, vjust = 1, hjust = 1, face = "bold.italic"),
80     axis.text.y = element_text(size = 14, face = "bold"),
81     axis.title.x = element_text(size = 16, face = "bold", vjust = -3),
82     axis.title.y = element_text(size = 16, face = "bold"),
83     plot.title = element_text(size = 20, face = "bold"),
84     legend.position = "none")  # Removes the legend
```

In this example:
- we have specified the dataset to use for the graph (line 53)
- the type of graph (line 54)
- added error bars and customized them (lines line 55)
- superimposed points that represent each trial over the bars (lines 56-60) added significance bars and stars (lines 61-72). Note that the testing for significance will be done in the next section, so these values come from there.
- added names to the graph and axes (lines 73-75)
- stylized everything according to a theme with edits (line 77-84).

Fig16. Line by line description of generating a plot

13 We could have added a line of code into the previous lines of code (52-84) that told R to create a graph from only one time point within **anoxia_data**. Another way to do this is to create a new object, dataset, that only contains the time point and trials that I want graphed. Personally, I opt for this to be able to examine each object; but, most people will opt for efficiency. This was done twice:

- lines 43-45:
- These lines of code generate a new, filtered, dataset.

```
42 #Filter only a specific time point
43 tenmin <- anoxia_data %>%
44   filter( time == 10) %>%
45   filter(trial %in% c(2, 3, 5))
```

This code tells R to go into the **anoxia_data** object we created in the previous section and create a filtered dataset from that (**tenmin**, line 43). I want to first only look at the ten-minute time point to compare to a previous publication (line 44). After seeing which trials were successful with the previous lines of code (lines 35-40), I only want to keep those in my new dataset (line 45).

- lines 48-50:
- Here, we create a new object that summarizes each trial as a mean value- to be used in the creation of superimposed points over the bar graph (line 56).

```
47 # Calculate the mean for each trial within each genotype
48 trial_means <- tenmin %>%
49   group_by(genotype, trial) %>%
50   summarize(mean_moving = mean(moving))
```

Fig17. Alternative to generating a graph of a single time point

Any object can be viewed by clicking on it in the environment pane. We can see our data organized by genotype, time, and moving. But only the ten minute time point is in this

dataset and only the successful trials. It is a good habit to check new objects you create to verify that R did what you wanted it to.

	trial	time	genotype	moving
1	2	10	egl-9	1
2	2	10	egl-9	1
3	2	10	egl-9	1
4	2	10	egl-9	1
5	2	10	egl-9	1
6	2	10	egl-9	1
7	2	10	egl-9	1
8	2	10	egl-9	0
9	2	10	hif-1	1
10	2	10	hif-1	1
11	2	10	hif-1	1
12	2	10	hif-1	0
13	2	10	hif-1	0
14	2	10	hif-1	0

Fig18. Always view your generated datasets

14 Next, let’s create a vector formatted version of our graph (.svg)

line 87:
These lines of code generate a .svg version of our graph.

```
#save your plot for export as an SVG vector format in your
computer files
ggsave("tenminplot.svg", plot = tenminplot, width = 8, height = 6)
```

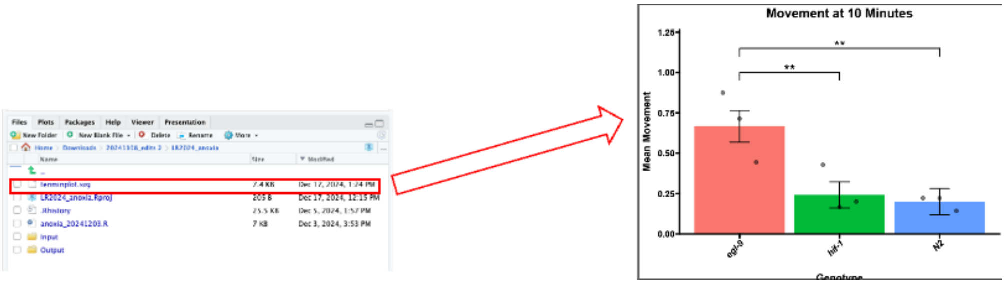

Fig19. Saving your plot as an alternative file format

Note that the new file saves to your working directory and is automatically updated in the computer and your files.

If you want to view this plot in Rstudio without saving a file to your computer in a particular format, you can just delete “tenminplot <-” text from this line of code. The graph will open in your “plots” tab in this same area you see your files. The way we do it here allows you to customize the export.

```
# Create the bar graph with trial means as points
tenminplot <- ggplot(tenmin, aes(x = genotype, y = moving, fill = genotype)) +
```

Fig20. Alternative way to view the plot in R Studio.

15 Now let’s move on to the more complex example of how to create a graph. This time, I want to see how the genotypes behave across the whole hour they were observed.

lines 89-96:
With these lines of code, we create a new object called df_anoxia_summary

```
df_anoxia_summary <- (anoxia_data %>%
  filter(trial %in% c(2,3,5)) %>%
  group_by(genotype, time) %>%
  summarise(
    mean_moving = mean(moving),
    # Calculate SEM using sqrt(variance / n)
    sem_moving = sqrt(sd(moving) / n())
  ))
```

This code tells R to go into the **anoxia_data** object we created in the previous section and to do several things within that data set. The pipe operator (%>%) allows us to accomplish multiple tasks at once.

- The first task is to **group_by** which asks R to reorganize our data first by genotype and then by time within that genotype.

- The second task is to **summarise**, which asks R to create a mean of the moving values at each time point in the respective genotype. We also want to know the variance, in this case the standard error (SEM) of the moving values. So we give R the math equation to calculate SEM.

16 Let's make our graph!

lines 98-118: With these lines of code, we create another kind of visualization.
For this plot, we have used the geom_line plot within ggplot2 (line 100). We have customized the SEM ribbon around the main line to be transparent cyan (line 60) the axes to reflect the fact that we are viewing data scaled to 100 (line 68) and distance between numbers on the x axis (line 67). Here is another resource for customizations and more information.

```
#plot mean values at each time point, with connected line and
shaded SEM
anoxiarecovery <- ggplot(df_anoxia_summary, aes(x = time, y =
mean_moving*100, color = genotype)) + #defines the data for the
graph
  geom_line() +  # Main line for mean
 # scale_color_manual(values = primary_colors) +  # Map primary
colors directly, can delete this line if no color scheme defined
  geom_ribbon(aes(ymin = mean_moving*100 - sem_moving*100,
                  ymax = mean_moving*100 + sem_moving*100),
              fill = "cyan", alpha = 0.2, linetype = "dashed") +
#this is the shaded standard deviation
  theme_prism() + #theme of choice, makes the background beige and
the text light grey
  theme(
    axis.text.x = element_text(size = 14, angle = 45, vjust = 1,
hjust = 1, face = "bold"),
    axis.text.y = element_text(size = 14, face = "bold"),
    axis.title.x = element_text(size = 16, face = "bold", vjust =
-1),
    axis.title.y = element_text(size = 16, face = "bold"),
    plot.title = element_text(size = 20, face = "bold"))+
  labs(title = "Mean anoxia recovery",
       x = "time (min)",
       y = "% animals moving",
       color = "Genotype") +
  # Increase the number of breaks for the x-axis
  scale_x_continuous(breaks = seq(min(0), max(60), length.out =
5)) +
  scale_y_continuous(expand = expansion())
```

Note that I have multiplied the “mean” values by 100 so that the y axis shows the % values and not the ratios.

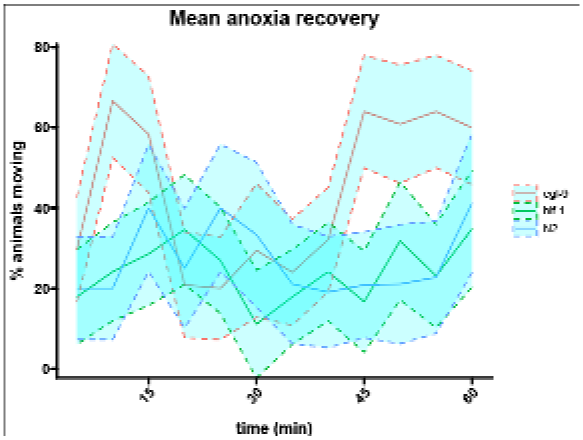

Fig21. Line plot from ggplot2

Expanding and Formatting Data for R Analysis

17 Install the following packages in the **console**: readxl, writexl, tidyr:

```
> install.packages("readxl")
> install.packages("writexl")
> install.packages("tidyr")
```

Load these libraries in the **source**:

```
library(readxl) # Read excel spreadsheets into R
library(writexl) # Write R data frames to excel
library(tidyr)  # Load the tidyr package
```

18 Direct the code to reference your raw data in a spreadsheet. The dataset in this file can be formatted as total observations. NOTE: The following example directs the program to access an "input" folder in the same location as where you saved your R program. Be sure that your code is modified to access the correct spreadsheet or move your data to be located adjacent to the R program file:

```
df <- read_excel("input/Male_Supplement_Aged_Control_AD2_5.xlsx")
```

19 The following code expands the data to be one line per observation and assigns a binary variable to each observation, where "1" indicates an event was observed and "0" means no events occurred. Then, the code combines all of the individual observations into a new dataset:

```
reshape_data <- function(df) {
  # Create a list to store individual worm observations
  data <- list()

  # Loop through each observation in the dataset
  for (i in 1:nrow(df)) {
    row <- df[i, ]

    # Extract original data except the title of the column, in
this case "Exopher" and "No_Exopher"
    original_data <- row[!(names(row) %in% c("Exopher",
"No_Exopher"))]

    # Add rows for instances where the data is positive for
"Exopher" with original data
    if (row$Exopher > 0) {
      for (j in 1:row$Exopher) {
        data[[length(data) + 1]] <- c(original_data, Exopher = 1,
No_Exopher = 0)
      }
    }

    # Add rows for "No_Exophers" events with original data
    if (row$No_Exopher > 0) {
      for (j in 1:row$No_Exopher) {
        data[[length(data) + 1]] <- c(original_data, Exopher = 0,
No_Exopher = 1)
      }
    }
  }

  # Combine worm observations into a data frame
  do.call(rbind, data)
}
```

20 Begin formatting the expanded dataset as a table and "print" the dataset to confirm visually that the data expansion looks correct in the console:

```
# Reshape the data frame- this will require tidyr later to format it as a table
df_expanded <- reshape_data(df)
# Print the expanded data frame
print(df_expanded)
```

	Trial	Treatment	Exopher	No_Exopher
1	1	AD2	9	41
2	1	AD5	0	50
3	2	AD2	14	36
4	2	AD5	3	47

Starting data looks like these total "Exopher" "No_Exopher" counts arranged in a table.

Filter

	Trial	Treatment	Exopher	No_Exopher
1	1	AD2	1	0
2	1	AD2	1	0
3	1	AD2	1	0
4	1	AD2	1	0
5	1	AD2	1	0
6	1	AD2	1	0
7	1	AD2	1	0
8	1	AD2	1	0
9	1	AD2	1	0
10	1	AD2	0	1
11	1	AD2	0	1
12	1	AD2	0	1
13	1	AD2	0	1
14	1	AD2	0	1
15	1	AD2	0	1

Showing 1 to 15 of 200 entries, 4 total columns

After reformatting, the data is expanded to represent one individual per row, with either a "1" or "0" for the Exopher and No_Exopher column.

21 Organize the data into a table with individual columns and check that the table looks correct in the console:

```
#use tidyr to make the lists individual columns, this is
formatting the data back into a table
data_trial_unnested <- unnest(data_expanded, Trial)
data_treatment_unnested <- unnest(data_trial_unnested, Treatment)
data_exopher_unnested <- unnest(data_treatment_unnested, Exopher)
data_final <- unnest(data_exopher_unnested, No_Exopher)

print(data_final) #check your work
```

```
> print(data_final) #check your work
# A tibble: 200 × 4
   Trial Treatment Exopher No_Exopher
   <dbl> <chr>       <dbl>      <dbl>
 1     1 AD2             1          0
 2     1 AD2             1          0
 3     1 AD2             1          0
 4     1 AD2             1          0
 5     1 AD2             1          0
 6     1 AD2             1          0
 7     1 AD2             1          0
 8     1 AD2             1          0
 9     1 AD2             1          0
10     1 AD2             0          1
# ℹ 190 more rows
```

The expanded data will look like this in the console

22 Save a new spreadsheet with the expanded dataset for subsequent analysis:

```
write_xlsx(data_final, "input/aged_exopher_final.xlsx") #write to
an excel file
```

Analyzing an exopher dataset with the Cochran-Mantel-Haenszel (CMH) test in R

23 Here, we use a simple exopher comparison to demonstrate implementing the CMH test and analysis of the CMH output.

This code requires installing the following packages and libraries: readxl and stats. In the **console:**

```
> install.packages("readxl")
> install.packages("writexl")
```

In the **source code**:

```
library(readxl) #read excel spreadsheets into R
library(stats) #basic statistical package
```

24 Import a properly formatted dataset:

```
#import your excel spreadsheet; n.b. this will only read the first
sheet in the workbook
data <- read_excel("input/aged_exopher_final.xlsx")
```

25 Reassign the datatypes into a format that works best for CMH analysis.

25.1 Convert the data to a dataframe for CMH analysis:

```
# Convert to data frame
data_df <- as.data.frame(data)
```

25.2 Convert the columns of the spreadsheet into factors:

```
# Convert columns to factors with two levels
data_df$Trial <- factor(data_df$Trial, levels =
unique(data_df$Trial))
data_df$Treatment <- factor(data_df$Treatment, levels =
unique(data_df$Treatment))
data_df$Exopher <- factor(data_df$Exopher, levels =
unique(data_df$Exopher))
```

26 Perform the CMH test.

```
# Note: The order of the factors matters in running this analysis.
# mantelhaen.test(x,y,z) where x is the row variable, y is the
column variable,
# and z is the stratifying factor. Generally, x refers to the
treatment groups
# (i.e. control vs. experimental group). y refers to the outcome
(is there an exopher?)
# and z refers to the replicates or trials.
#
# Arranging the factors in this order tests for the association of
treatment and
# exopher while controlling for differences between trials.
Essentially the Mantel-Haenszel
# test examines each trial separately before aggregating the
results to conclude
# if there is an overall association between the treatment and
exophers across all
# trials.

data_CMH <- mantelhaen.test(data_df$Treatment, data_df$Exopher,
data_df$Trial)
```

27 Print the result to the console:

```
print(data_CMH)
```

```
> print(data_CMH)

        Mantel-Haenszel chi-squared test with continuity correction

data:  data_df$Treatment and data_df$Exopher and data_df$Trial
Mantel-Haenszel X-squared = 16.026, df = 1, p-value = 6.246e-05
alternative hypothesis: true common odds ratio is not equal to 1
95 percent confidence interval:
  2.882464 36.514733
sample estimates:
common odds ratio
         10.25926
```

Example output from the CMH calculation.

Note that the CMH test produces an X-squared value rather than a Z-value, the X-squared value is stripped of its directionality (i.e. if there is an increase or decrease in exophers).

Our p-value is very significant, as shown by the 95% confidence interval, which does not include "1".

Furthermore, the odds ratio provides information about the magnitude of the difference, indicating a 10.2-fold difference between the treatments.

To calculate the risk estimate and risk difference from the CMH dataset you would start with the 2×2 contingency table and calculate the following:

- *Unconditional risk estimates* → sum exopher incidences across strata and divide by the **total number of subjects** in that treatment group.
- *Unconditional risk difference* → difference between those risks.
- *Stratified (CMH) risk difference* → comes from the CMH numerator divided by total N.

27.1 Calculating the signed z-value from the CMH statistic:

```
chisq_val <- unname(data_CMH$statistic)  # this is Z^2
```

The CMH Z should be positive if the second level of your Treatment factor is associated with a higher probability of “Yes” in Exopher, and negative otherwise.

You can use the Mantel–Haenszel odds ratio from the test:

```
mh_or <- unname(data_CMH$estimate)       # Mantel–Haenszel pooled
odds ratio
sign_val <- sign(log(mh_or))             # +1 or -1 depending on
direction
```

Combine into signed Z

```
Z_val <- sign_val * sqrt(chisq_val)
Z_val
```

ANOVA in R - anoxia example

28 Going back to our bar graph of the ten minute time point, let’s see if the means vary significantly.

lines 125-129:
With these lines of code, we are conducting a one-way ANOVA (line 126) on a filtered data set at 10 min (line 126), with a Tukey’s post-hoc multiple comparisons test (line 127). We then create a summary of the ANOVA (tenminaov).

```
#one-way anova at a single time point
tenminaov <- aov(moving ~ genotype, data = tenmin)
TukeyHSD(tenminaov)
summary(tenminaov)
print(tenminaov)
```

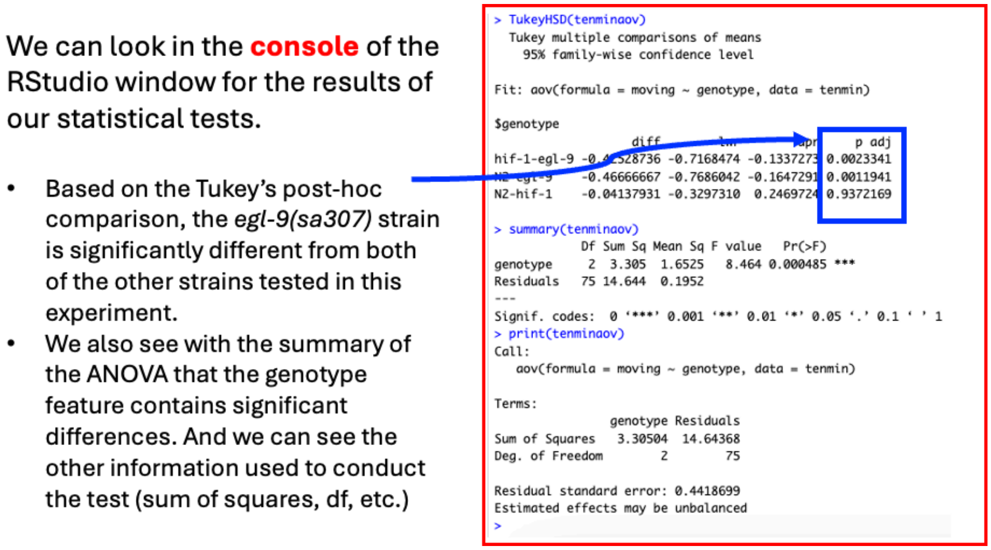

Fig22. Interpreting ANOVA in R

Generating a logistic regression in R

29 As argued in our text, the most appropriate test to compare our time series behavioral data is a logistic regression.

```
##simple logistic regression
an_glm <- glm(moving ~ genotype_unordered, family = binomial(link
= "logit"), # the default is logit
               data = anoxia_data)
summary(an_glm)
plot(an_glm)
```

With these lines of code, we are conducting a logistic regression using a generalized linear mode (lines 136-139).

Notice that we are telling the model to consider the differences within the moving values amongst the genotypes.

We can view the results of the test by asking for a summary of the object we created an_glm (line 138) and ask R to plot the logistic for us (line 139).

Next, we will move onto our logistic regression example. But first! Let's do a little trick to make sure that the model knows which genotype to compare the others to, in our case this I want to compare everything to (the reference) the wild type ("N2").

```
# Define genotype as a factor and make N2 (our wild type strain)
your reference sequenced
genotype_unordered <- factor(anoxia_data$genotype, ordered =
FALSE) #makes genotype a factor
genotype_unordered <- relevel(genotype_unordered, ref = "N2")
#makes N2 within genotype the reference genotype
```

We first define genotype as a factor- this is somewhat unnecessary since this is clearly a categorical variable, but sometimes it is better to make sure the model won't make assumptions (line 132). We next tell R that the reference strain is "N2" (line 133).

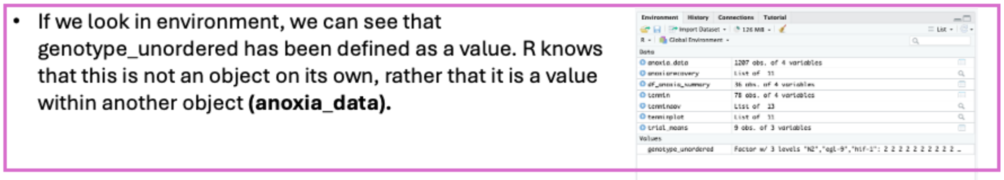

Fig23. Viewing the ordered genotype group in R Studio

Let's again look in the console to see the results of the test. **Here is another resource** that accessibly goes over the results of the logistic GLM in more detail.

```
> summary(an_glm)

Call:
glm(formula = moving ~ genotype_unordered, family = binomial(link = "logit"),
    data = anoxia_data)

Coefficients:
                        Estimate Std. Error z value Pr(>|z|)
(Intercept)              -0.8948     0.1224  -7.309 2.69e-13 ***
genotype_unorderedegl-9   0.4123     0.1550   2.660  0.00781 **
genotype_unorderedhif-1  -0.1342     0.1656  -0.810  0.41792
---
Signif. codes:  0 '***' 0.001 '**' 0.01 '*' 0.05 '.' 0.1 ' ' 1

(Dispersion parameter for binomial family taken to be 1)

    Null deviance: 1506.8  on 1206  degrees of freedom
Residual deviance: 1491.2  on 1204  degrees of freedom
AIC: 1497.2

Number of Fisher Scoring iterations: 4
```

Fig24. Interpreting Logistic Regression in R

Notice the column called "Estimate Std,"
these values are also known as the coefficients. The intercept is the log odds
of moving for moving for the reference group. We can take the exponent of the
values here to give us an odds ratio. And we can do this all within R! Just
write in the console:

```
>exp(0.4123)
```

This is the coefficient of egl-9 versus
N2. The result:
[1] 1.510287
Tells us that when compared to wild type, *egl-9* has a 1.5 positive odds of moving. You can see for *hif-1*, there is a negative coefficient, so this means that this strain was observed as moving less across the time points than N2. Yet, this was not significant, as we can see from the Pr. column. This P value is calculated from the z value and the ratio of the Std. Error.

30 After we run the GLM (lines 136-137), view the statistical summary (line 138), we ask R to plot the results (line 139)

To plot the GLM, you will run the code in line 139. Then, go to console, and as R prompts you above, hit "Enter/Return" on your keyboard.

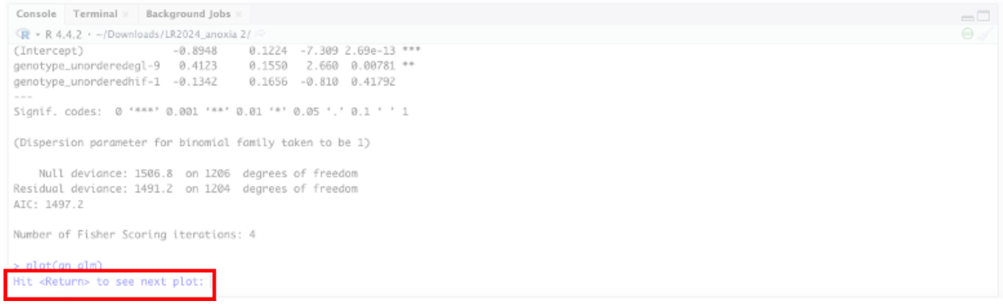

Fig25. Plotting results of a GLM in R

R has a built-in way to plot the results of the GLM; they are used for troubleshooting your model. The interpretation of these graphs goes beyond the scope of the statistical expertise of this project
(i.e. please consult your resident biostatistician); but we provide basic definitions here.

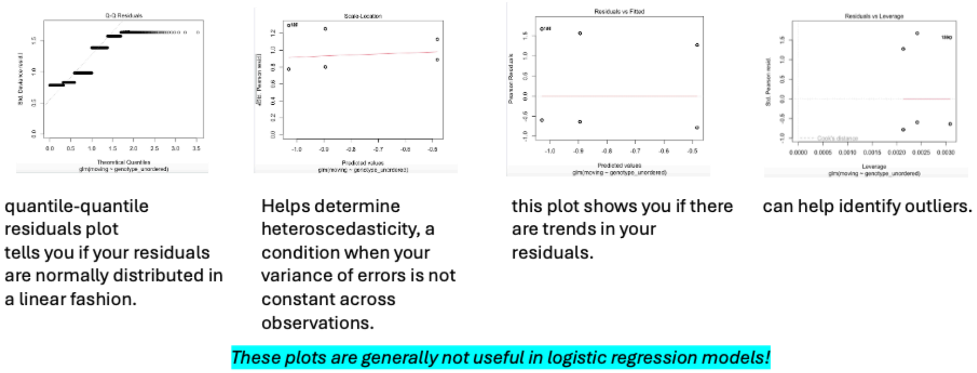

Fig26. Interpreting GLM plots in R

31 Finally, let’s apply a more complex model to the time series behavioral data: the generalized additive model (GAM). This regression model is useful for data that contains non-linear relationships. In our data, it is quite clear that there is a general pattern of behavior, but I really want to be able to define that pattern. A GAM can also help with this.

```
#general additive model
an_gam <- gam(moving ~ genotype_unordered + s(time), data =
anoxia_data)
summary(an_gam)
```

GAM is a regression model and the format of the GAM code is similar to the GLM code. We define what it is to be compared; in this case, its moving versus the behavior of genotype over time.

We can ask R to plot this model. Here, there are three iterations of this.

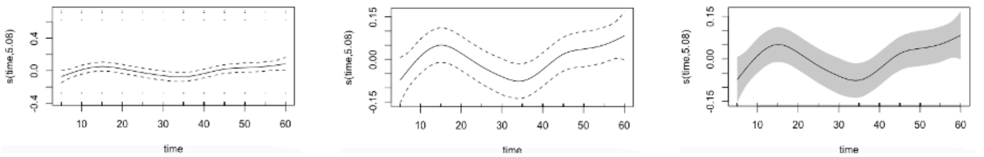

line 144: the basic graph

line 145: adjusted y scale

line 146: and because I like the aesthetic of it, I have shaded in the standard error

Fig27. Plotting a GAM in R

Protocol references

Works Cited

CRAN. "The Comprehensive R Archive Network." R-Project.org, 2019, cran.r-project.org/.

"Curious Black Persian Cat Exploring Python Coding | Website Name | AI Art Generator | Easy-Peasy.AI." Easy-Peasy.AI, 2024, easy-peasy.ai/ai-image-generator/images/curious-black-persian-cat-exploring-python-coding. Accessed 18 Sept. 2024.

Edwards, Benj, and Nick Lewis. "How to Copy the Full Path of a File on Windows 10." How-to Geek, How-To Geek, 7 May 2020, www.howtogeek.com/670447/how-to-copy-the-full-path-of-a-file-on-windows-10/. Accessed 18 Sept. 2024.

"Get File, Folder, and Disk Information on Mac." Apple Support, support.apple.com/guide/mac-help/get-file-folder-and-disk-information-on-mac-mchlp1774/mac.

"Ggplot2 Line Plot : Quick Start Guide - R Software and Data Visualization - Easy Guides - Wiki - STHDA." Www.sthda.com, www.sthda.com/english/wiki/ggplot2-line-plot-quick-start-guide-r-software-and-data-visualization.

"How to Find Full Path of a File in Linux." Linuxhandbook.com, linuxhandbook.com/get-file-path/.

"Logit Regression | R Data Analysis Examples." Stats.oarc.ucla.edu, stats.oarc.ucla.edu/r/dae/logit-regression/.

"Posit." Posit, posit.co/downloads/.

"Quick-R: R Packages." www.statmethods.net, www.statmethods.net/interface/packages.html.

"RStudio User Guide - Pane Layout." Docs.posit.co, 12 Dec. 2022, docs.posit.co/ide/user/ide/guide/ui/ui-panes.html.

Wickham, Hadley. "Create Elegant Data Visualisations Using the Grammar of Graphics." Tidyverse.org, 2019, ggplot2.tidyverse.org/.

Acknowledgements

We thank Nelson Mejia, Ryan Nyugen, and Mark Saba for testing our code and providing comments.
We also thank the Caenorhabditis Genetics Center (CGC, founded by National Institutes of Health - Office of Research Infrastructure Programs (P40OD010440)) for providing some strains.
