# Supplementary material for: Analysis of categorical data from biological experiments with logistic regression and CMH tests
Source: PLoS One. 2025 Nov 17;20(11):e0335143. doi: 10.1371/journal.pone.0335143 (PMC12622779; doi:10.1371/journal.pone.0335143)
Supplement: S2 File — (ZIP) [file pone.0335143.s003.zip › Logistic-Regression-for-Biologists-main/Logistic_Regression_Aging/Formatting_Input_Aging_Code_Annotated.pdf]

Highlighted text are parts of this code that may need to be optimized to match your dataset

Change this to match the name of your raw dataset in the input folder

Change these variables to match the column headers in your dataset

These variables reference the column titles in your dataset

Indicate where you want to save the reformatted data and what you want to name the final spreadsheet

```
# Use this code to reformat your raw data from total instances into an excel
# spreadsheet listing each observation as a separate row.

library(readxl) #read excel spreadsheets into R
library(writexl) #write R data frames to excel
library(tidyr)  # Load the tidyr package

df <- read_excel("input/Male_Supplement_Aged_Control_AD2_5.xlsx")

reshape_data <- function(df) {
  # Create a list to store individual worm observations
  data <- list()

  # Loop through each observation in the dataset
  for (i in 1:nrow(df)) {
    row <- df[i, ]

    # Extract original data except the title of the column, in this case "Exopher" and
"No_Exopher"
    original_data <- row[!(names(row) %in% c("Exopher", "No_Exopher"))]

    # Add rows for instances where the data is positive for "Exopher" with original data
    if (row$Exopher > 0) {
      for (j in 1:row$Exopher) {
        data[[length(data) + 1]] <- c(original_data, Exopher = 1, No_Exopher = 0)
      }
    }

    # Add rows for "No_Exophers" events with original data
    if (row$No_Exopher > 0) {
      for (j in 1:row$No_Exopher) {
        data[[length(data) + 1]] <- c(original_data, Exopher = 0, No_Exopher = 1)
      }
    }
  }

  # Combine worm observations into a data frame
  do.call(rbind, data)
}

# Reshape the data frame- this will require tidyr later to format it as a table
df_expanded <- reshape_data(df)
# Print the expanded data frame
print(df_expanded)

data_expanded <- as.data.frame(df_expanded)
str(data_expanded) #checks the structure of the data- if the  result is a "list" then you
need to use tidyr to clean it up

#use tidyr to make the lists individual columns, this is formatting the data back into a
table
data_trial_unnested <- unnest(data_expanded, Trial)
data_treatment_unnested <- unnest(data_trial_unnested, Treatment)
data_exopher_unnested <- unnest(data_treatment_unnested, Exopher)
data_final <- unnest(data_exopher_unnested, No_Exopher)

print(data_final) #check your work
write_xlsx(data_final, "input/aged_exopher_final.xlsx") #write to an excel file
```
