# Supplementary material for: Analysis of categorical data from biological experiments with logistic regression and CMH tests
Source: PLoS One. 2025 Nov 17;20(11):e0335143. doi: 10.1371/journal.pone.0335143 (PMC12622779; doi:10.1371/journal.pone.0335143)
Supplement: S2 File — (ZIP) [file pone.0335143.s003.zip › Logistic-Regression-for-Biologists-main/Logistic_Regression_Aging/Logistic_Regression_Aging_Code_Annotated.pdf]

Highlighted text are parts of this code that may need to be optimized to match your dataset

```
################################################
# Regression models on exopher frequency data #
# September 12 2025                           #
################################################

#text like this with a hashtag before it is called a comment and is not executable code.
#to execute a line of code on a mac, go to the end of the line and hit command+shift+enter
####<---you can use the arrows next to the lines numbers to collapse or fold in a section####



####section 1:setup####

#First! install the following packages using install.packages("name of package") in the CONSOLE of
RStudio:
#dplyr
#readxl
#writexl
#ggplot2
#ggthemes
#ggthemeAssist
#stats
#ggsignif
#ggpubr
#ggprism

#next, load the libraries by executing code in the upper part of the screen where all this code is.
Again, to execute a line of code on a mac, go to the end of the line and hit command+shift+enter
library(dplyr) #datamanipulation
library(readxl) #read excel spreadsheets into R
library(writexl) #convert R data frames into excel spreadsheet
library(ggplot2) #visualizations besides basics plots
library(ggthemes) #adds more themes to the ggplot2 package
library(ggThemeAssist)
library(stats) #basic statistical package
library(ggsignif) #provides a graphical user interface (GUI) for customizing ggplot2 themes
library(ggpubr) #To add significance stars to your ggplot2 graph
library(ggprism) #To generate the plots in a minimalist theme

#import your excel spreadsheet; n.b. this will only read the first sheet in the workbook
data <- read_excel("input/aged_exopher_final.xlsx")
```

Change this to match the name of your raw dataset in the input folder

```
####section 2:see what your data looks like####

##generate plot of your dataframe. This section produces a graph of the data but
#this code is not a prerequisite for performing the statistical analysis and can
#be skipped if you prefer to generate a graph through another program.

#summarizing data as percentages to plot
data_percent <- data %>%
  group_by(Treatment, Trial) %>%
  summarize(
    data_percent = (100 * ((sum(Exopher, na.rm = TRUE)) / n()))
  )
```

Change these variables to match the column headers in your dataset
**Capitalization matters

```
data_summary <- data %>%
  group_by(Treatment, Trial) %>%
  summarize(
    data_percent = (100 * ((sum(Exopher, na.rm = TRUE)) / n())),
    # Calculate standard error of the percentage (SEP) using the formula sqrt((p * (1 - p)) / n)
    data_sep = sqrt((data_percent / 100) * (1 - (data_percent / 100)) / n()) * 100
```

```
  ) %>%
  group_by(Treatment) %>%
  summarize(
    data_percent_mean = mean(data_percent),
    data_sep_mean = mean(data_sep)  # Average SEP across trials
  )

#plot exopher percentages and SEP

# Merge the datasets by a common column (assuming 'Treatment' is common to both)
merged_data <- merge(data_summary, data_percent, by = "Treatment", all.x = TRUE)

# Create the plot
# Plot the data with individual points from the merged dataset
# Assuming you have merged `data_summary` and `data_percent` into a dataframe
# called `merged_data`:

# Create a bar plot with significance brackets and a scatterplot overlay
Plotted_data <- ggplot(data_summary, aes(x = Treatment, y = data_percent_mean, fill = Treatment)) +

  # Add bars with black borders
  geom_bar(stat = "identity", width = 0.7, color = "black", size = 1.5) +

  # Overlay a scatterplot to show individual trial averages
  geom_point(data = data_percent,  # Use the trial_means data
             aes(x = Treatment, y = data_percent, group = Trial),  # Map trial means
             position = position_dodge(width = 0.5),
             alpha = 0.5,
             size= 2.5)  +

  # Add error bars
  geom_errorbar(aes(ymin = data_percent_mean - data_sep_mean, ymax = data_percent_mean +
data_sep_mean),
                width = 0.2, linetype = "solid", linewidth = 1.5) +

  # Add significance annotations with a thicker bracket line
  geom_signif(comparisons = list(c("AD2", "AD5")),
              annotations = c("***"),
              y_position = 30,  # Place the bracket above the bars
              textsize = 8,
              tip_length = 0.05,
              vjust = 0.5,
              size = 1.5,  # Increase bracket line thickness
              linetype = "solid", color = "black") +  # Solid line and black color

  # Set y-axis limits and ensure there's space for the significance annotation
  coord_cartesian(ylim = c(0, 33)) +  # Shortened y-limit to 30

  # Adjust the x-axis horizontal line to intersect with 0 (using expand)
  scale_y_continuous(expand = c(0, 0)) +  # Removes the padding around the plot

  # Use a simple theme
  theme_prism() +
  theme(
    legend.position = "none",  # Remove the legend
    axis.text.x = element_text(size = 20, angle = 45, hjust = 1.1, vjust = 1, face = "bold", color
= "black"),  # x-axis labels left-aligned
    axis.text.y = element_text(size = 20, face = "bold", color = "black"),
    axis.title.x = element_text(size = 22, face = "bold", color = "black", hjust = 1),  # Adjust
hjust for x-axis title alignment
    axis.title.y = element_text(size = 22, face = "bold", color = "black"),
```

Indicate the variables within the Treatment group

```
    plot.title = element_text(size = 24, face = "bold", color = "black", hjust = 0.5)
  ) +
  # Add the title to the plot
  labs(
    title = "Exopher Frequency",  # Plot title
    x = "Days of Adulthood",     # X-axis title
    y = "Percent Exophers"      # Y-axis title
  )
```

Specify the titles for your graph and the axes

```
# Print the plot
print(Plotted_data)


# Save the plot as an SVG file (vector format)
ggsave("exopher_frequency_plot.svg",
       plot = last_plot(),
       width = 4,
       height = 6,
       units = "in")

####section 3:statistical analysis####


###Cochran-Mantel-Haenszel (CMH) test of Exopher frequency

# Convert to data frame
data_df <- as.data.frame(data)
```

Change these variables to match the column headers in your dataset

```
# Convert columns to factors with two levels
data_df$Trial <- factor(data_df$Trial, levels = unique(data_df$Trial))
data_df$Treatment <- factor(data_df$Treatment, levels = unique(data_df$Treatment))
data_df$Exopher <- factor(data_df$Exopher, levels = unique(data_df$Exopher))

# Perform the Mantel-Haenszel test

# Note: The order of the factors matters in running this analysis.
# mantelhaen.test(x,y,z) where x is the row variable, y is the column variable,
# and z is the stratifying factor. Generally, x refers to the treatment groups
# (i.e. control vs. experimental group). y refers to the outcome (is there an exopher?)
# and z refers to the replicates or trials.
#
# Arranging the factors in this order tests for the association of treatment and
# exopher while controlling for differences between trials. Essentially the Mantel-Haenszel
# test examines each trial separately before aggregating the the results to conclude
# if there is a overall association between the treatment and exophers across all
# trials.

data_CMH <- mantelhaen.test(data_df$Treatment, data_df$Exopher, data_df$Trial)

# Print the result
print(data_CMH)

## Calculating the signed z-value from the CMH statistic

chisq_val <- unname(data_CMH$statistic)  # this is Z^2

# The CMH Z should be positive if the second level of your Treatment factor is
# associated with a higher probability of “Yes” in Exopher, and negative otherwise.

# You can use the Mantel-Haenszel odds ratio from the test:
```

```
mh_or <- unname(data_CMH$estimate)       # Mantel-Haenszel pooled odds ratio
sign_val <- sign(log(mh_or))             # +1 or -1 depending on direction

# Combine into signed Z

Z_val <- sign_val * sqrt(chisq_val)
Z_val


##Reformatting the data for logistic regression
# Define Treatment as a factor and make AD2 (Adult Day 2) your reference dataset
Treatment_unordered <- factor(data$Treatment, ordered = FALSE) #makes Treatment a factor
Treatment_unordered <- relevel(Treatment_unordered, ref = "AD2") #makes AD2 within Treatment the
reference condition

##simple logistic regression
data_glm <- glm(Exopher ~ Treatment_unordered+factor(Trial), family = binomial(link = "logit"), #
the default is logit
                data = data)
summary(data_glm)
plot(data_glm)

# Calculate odds ratios
odds_ratios <- exp(coef(data_glm))
print(odds_ratios)

# Calculate 95% confidence intervals for the odds ratios
conf_intervals <- exp(confint(data_glm))
print(conf_intervals)
```
