# Supplementary material for: Analysis of categorical data from biological experiments with logistic regression and CMH tests
Source: PLoS One. 2025 Nov 17;20(11):e0335143. doi: 10.1371/journal.pone.0335143 (PMC12622779; doi:10.1371/journal.pone.0335143)
Supplement: S2 File — (ZIP) [file pone.0335143.s003.zip › Logistic-Regression-for-Biologists-main/Logistic_Regression_Aging/Logistic_Regression_Aging_Output_Annotated.pdf]

```
> ####section 3:statistical analysis####
> 
> 
> ###Cochran-Mantel-Haenszel (CMH) test of Exopher frequency
> 
> # Convert to data frame
> data_df <- as.data.frame(data)
> 
> # Convert columns to factors with two levels
> data_df$Trial <- factor(data_df$Trial, levels = unique(data_df$Trial))
> data_df$Treatment <- factor(data_df$Treatment, levels = unique(data_df$Trea
tment))
> data_df$Exopher <- factor(data_df$Exopher, levels = unique(data_df$Exopher)
)
> 
> # Perform the Mantel-Haenszel test
> data_CMH <- mantelhaen.test(data_df$Treatment, data_df$Exopher, data_df$Tri
al)
> 
> # Print the result
> print(data_CMH)

        Mantel-Haenszel chi-squared test with continuity correction

data:  data_df$Treatment and data_df$Exopher and data_df$Trial
Mantel-Haenszel X-squared = 16.026, df = 1, p-value = 6.246e-05
alternative hypothesis: true common odds ratio is not equal to 1
95 percent confidence interval:
  2.882464 36.514733
sample estimates:
common odds ratio
         10.25926

> 
> 
> 
> ##Reformatting the data for logistic regression
> # Define Treatment as a factor and make AD2 (Adult Day 2) your reference da
taset
> Treatment_unordered <- factor(data$Treatment, ordered = FALSE) #makes Treat
ment a factor
> Treatment_unordered <- relevel(Treatment_unordered, ref = "AD2") #makes AD2
within Treatment the reference condition
> 
> ##simple logistic regression
> data_glm <- glm(Exopher ~ Treatment_unordered+factor(Trial), family = binom
ial(link = "logit"), # the default is logit
+                 data = data)
> summary(data_glm)

Call:
glm(formula = Exopher ~ Treatment_unordered + factor(Trial),
    family = binomial(link = "logit"), data = data)

Coefficients:
                       Estimate Std. Error z value Pr(>|z|)
(Intercept)             -1.6502     0.3688  -4.474 7.66e-06 ***
Treatment_unorderedAD5  -2.2980     0.6358  -3.614 0.000301 ***
factor(Trial)2           0.7978     0.4594   1.737 0.082433 .
---
Signif. codes:  0 ‘***’ 0.001 ‘**’ 0.01 ‘*’ 0.05 ‘.’ 0.1 ‘ ’ 1

(Dispersion parameter for binomial family taken to be 1)
```

CMH p-value

Odds ratio from the CMH test, there is a 10.2 times greater chance of producing an exopher in the control group compared to the treatment group.

These are the results for the comparison between AD2 (control treatment) and AD5.

Logistic regression results. The estimate is used to calculate the odds ratio. The z-value indicates standard deviations away from the mean and a negative z-value means that the AD5 treatment produces fewer exophers. Pr(>|z|) is the p-value for the logistic.

```
    Null deviance: 154.55  on 199  degrees of freedom
Residual deviance: 131.66  on 197  degrees of freedom
AIC: 137.66

Number of Fisher Scoring iterations: 6

> plot(data_glm)
Hit <Return> to see next plot:
Hit <Return> to see next plot:
Hit <Return> to see next plot:
Hit <Return> to see next plot:
> # Calculate odds ratios
> odds_ratios <- exp(coef(data_glm))
> print(odds_ratios)
           (Intercept) Treatment_unorderedAD5         factor(Trial)2
             0.1920029              0.1004630              2.2206918
>
> # Calculate 95% confidence intervals for the odds ratios
> conf_intervals <- exp(confint(data_glm))
Waiting for profiling to be done...
> print(conf_intervals)
                            2.5 %    97.5 %
(Intercept)            0.08724927 0.3768432
Treatment_unorderedAD5 0.02310860 0.3043337
factor(Trial)2         0.92085171 5.6748830
```

Odds ratio for the logistic regression. There is only a 10% chance for an exopher to occur on AD5 as compared to AD2.

95% confidence interval for the logistic regression. This range does not include 1 indicating that the odds ratio is statistically significant.
