# Supplementary material for: Analysis of categorical data from biological experiments with logistic regression and CMH tests
Source: PLoS One. 2025 Nov 17;20(11):e0335143. doi: 10.1371/journal.pone.0335143 (PMC12622779; doi:10.1371/journal.pone.0335143)
Supplement: S2 File — (ZIP) [file pone.0335143.s003.zip › Logistic-Regression-for-Biologists-main/Logistic_Regression_Anoxia 2/Logistic_Regression_Anoxia/20241217_tutorial.pptx]

## Slide 1
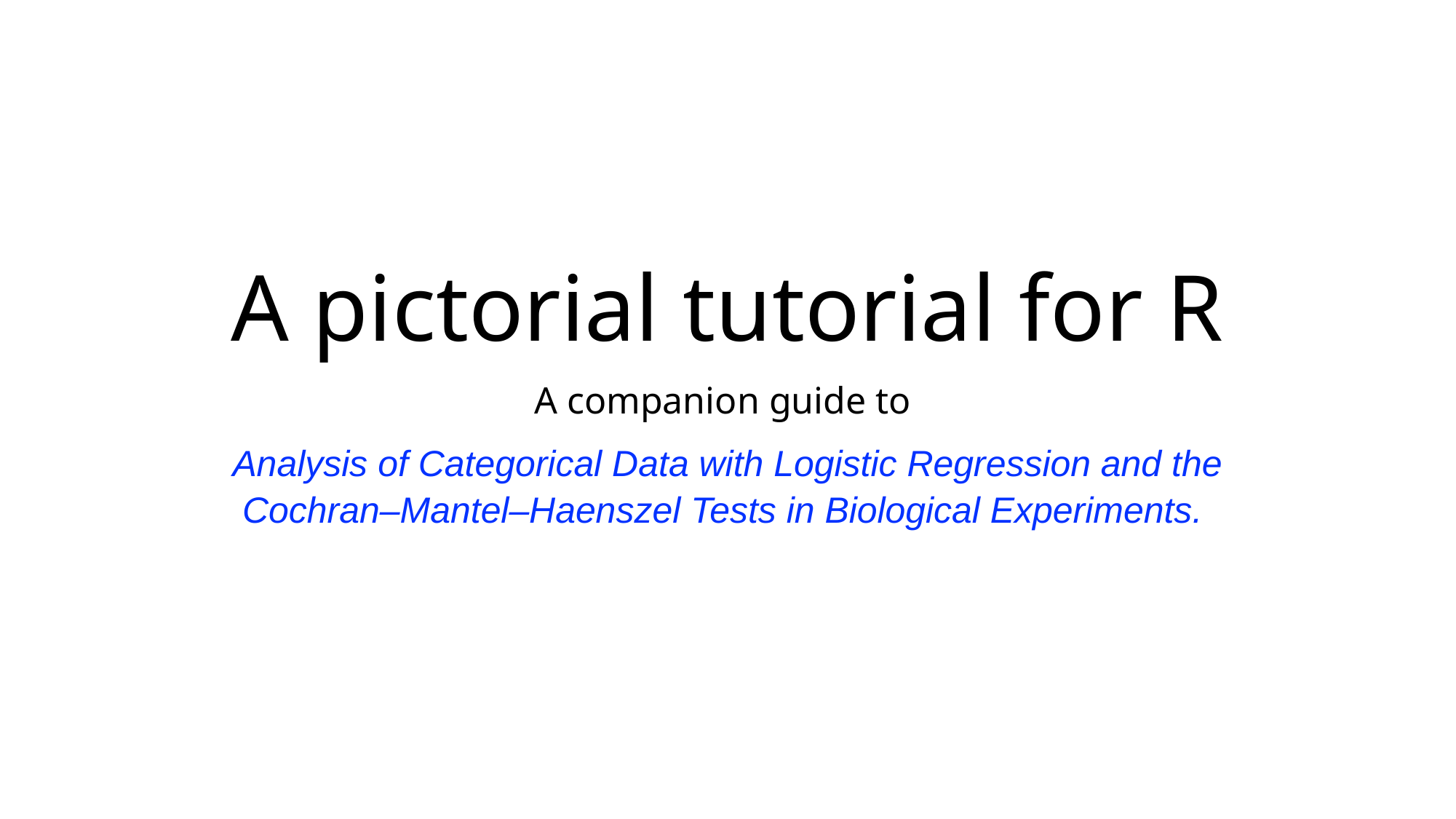

# A pictorial tutorial for R
A companion guide to
Analysis of Categorical Data with Logistic Regression and the Cochran–Mantel–Haenszel Tests in Biological Experiments.

## Slide 2
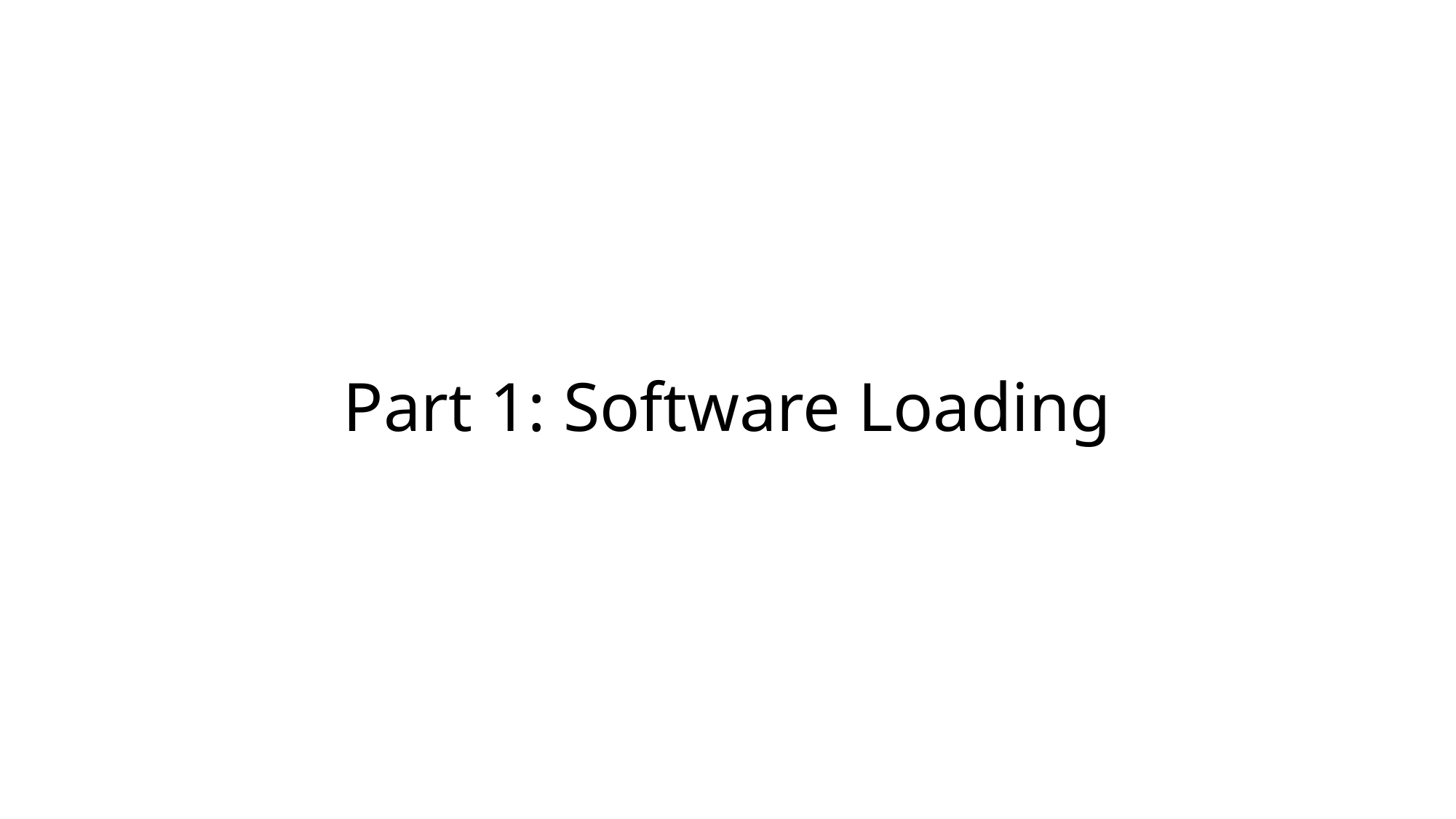

# Part 1: Software Loading

## Slide 3
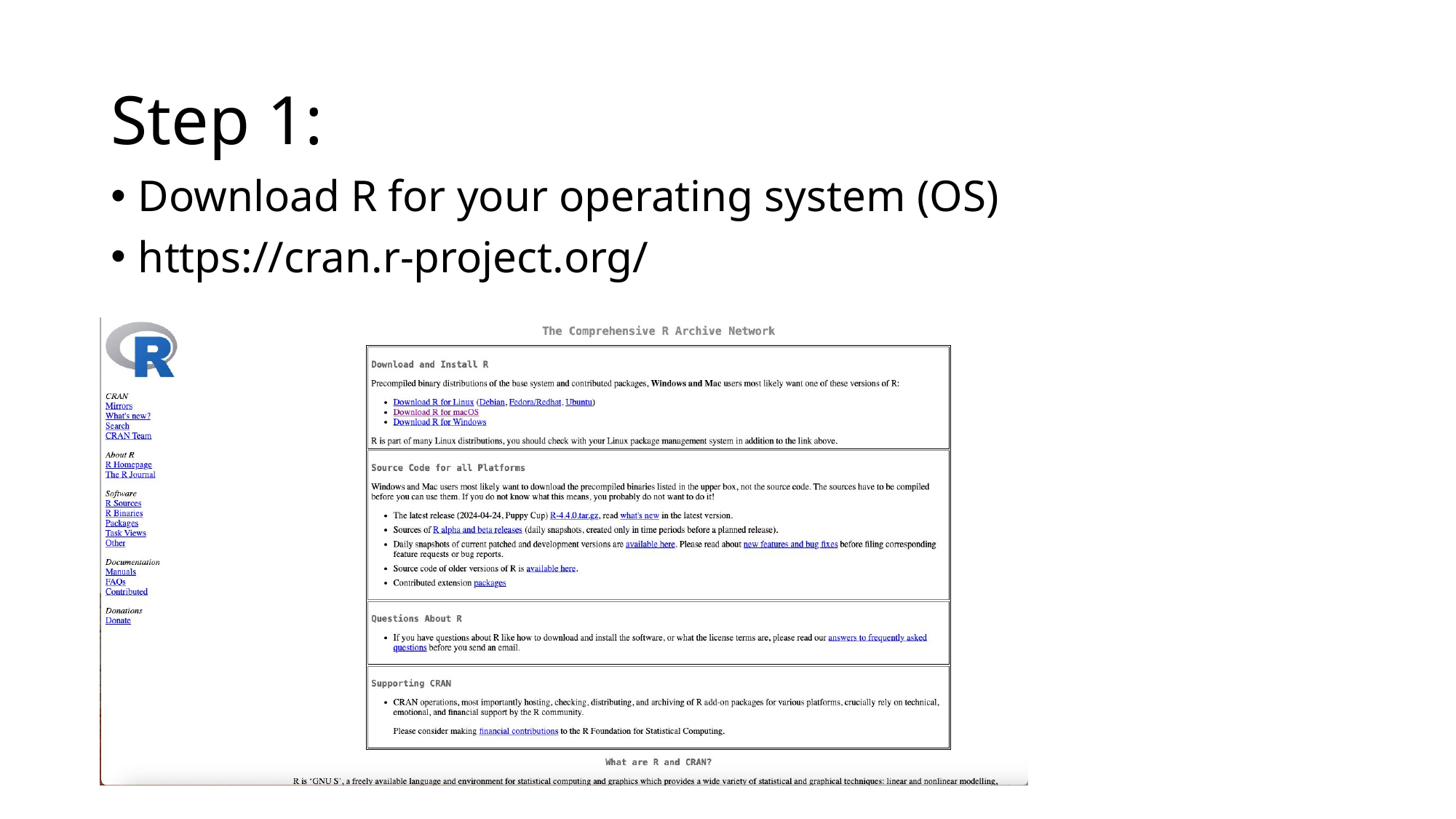

# Step 1:
Download R for your operating system (OS)
https://cran.r-project.org/

## Slide 4
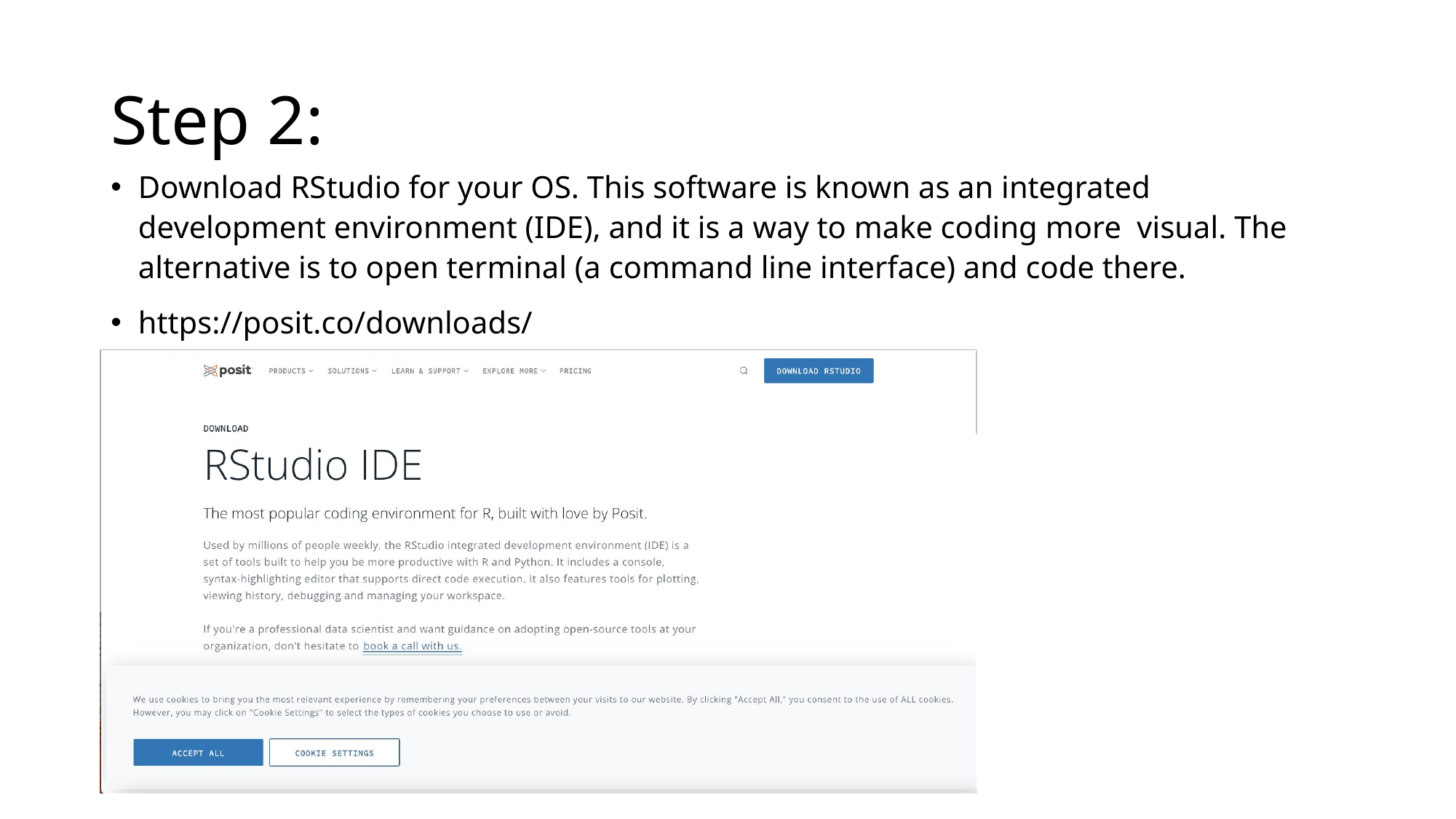

Step 2:
Download RStudio for your OS. This software is known as an integrated development environment (IDE), and it is a way to make coding more visual. The alternative is to open terminal (a command line interface) and code there.
https://posit.co/downloads/

## Slide 5
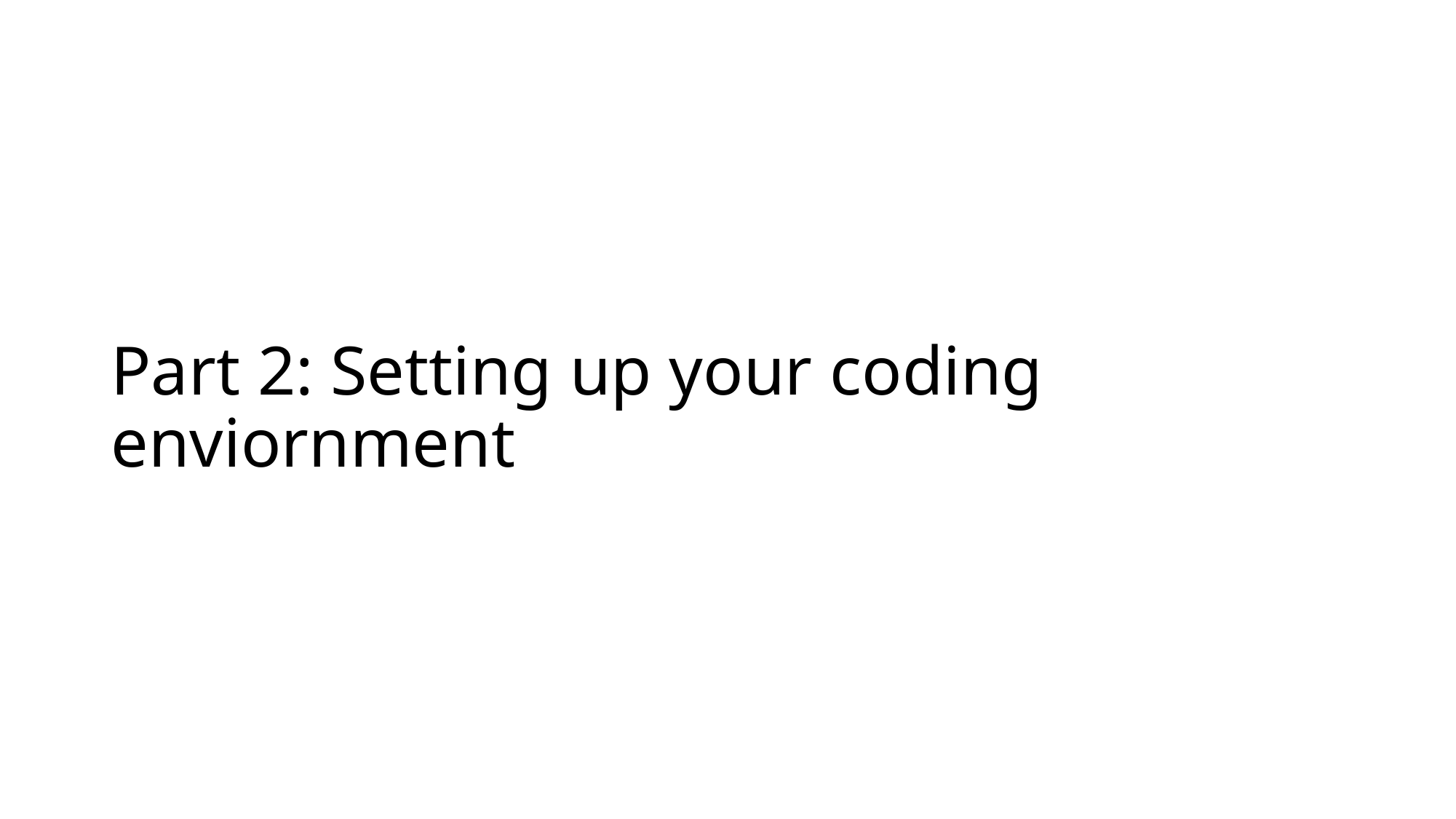

# Part 2: Setting up your coding enviornment

## Slide 6
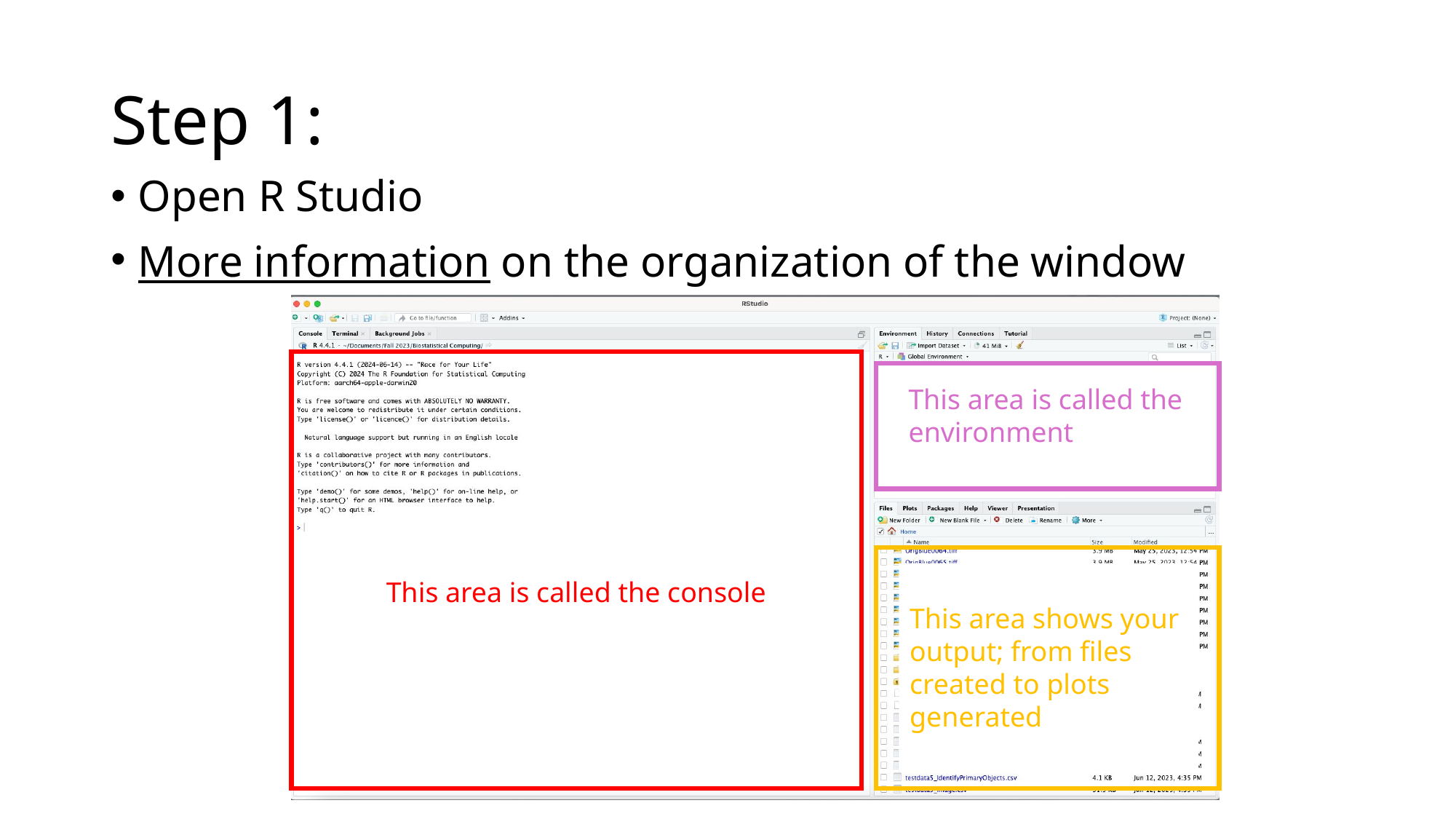

Step 1:
Open R Studio
More information on the organization of the window
This area is called the environment
This area shows your output; from files created to plots generated
This area is called the console

## Slide 7
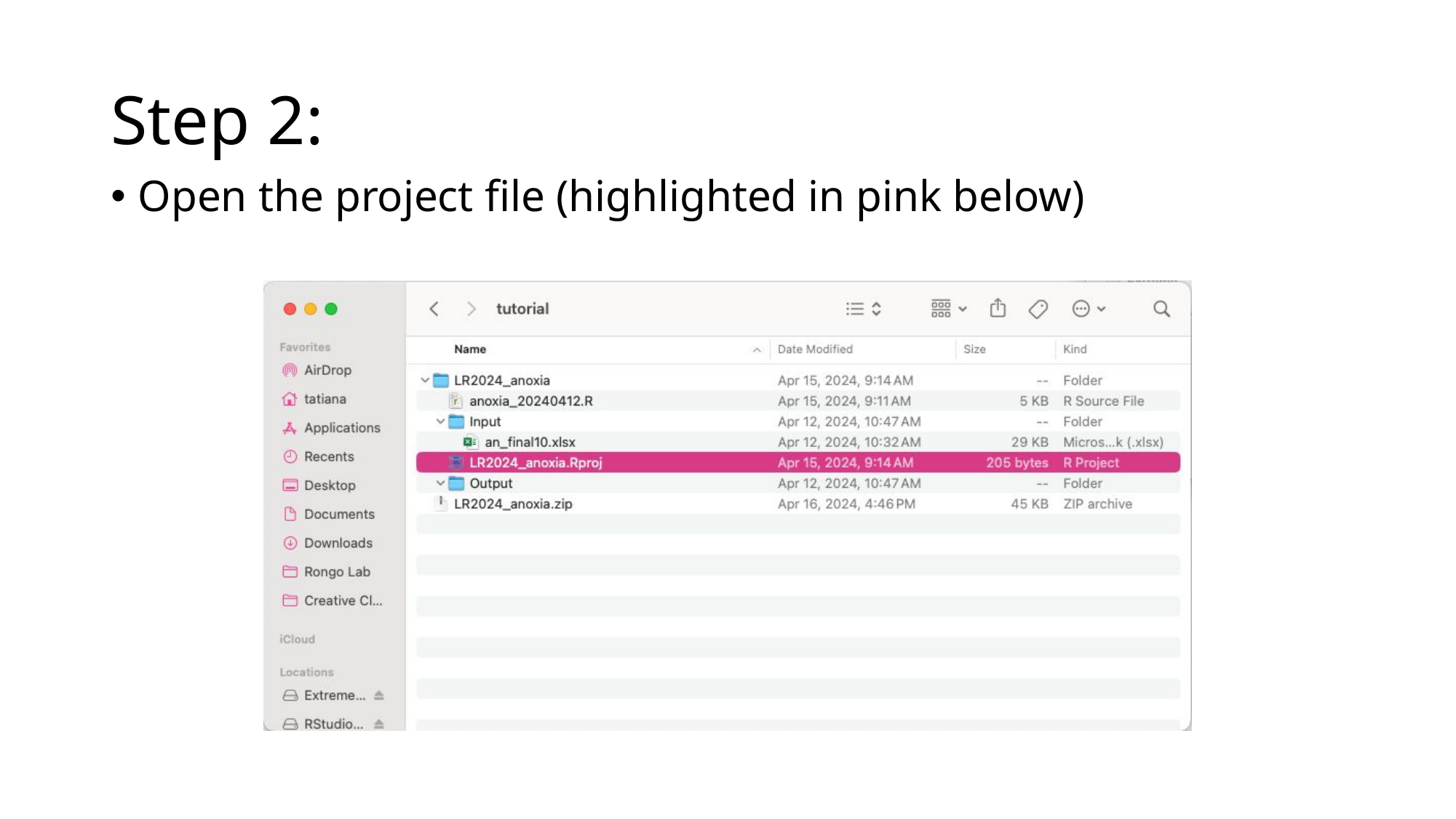

Step 2:
Open the project file (highlighted in pink below)

## Slide 8
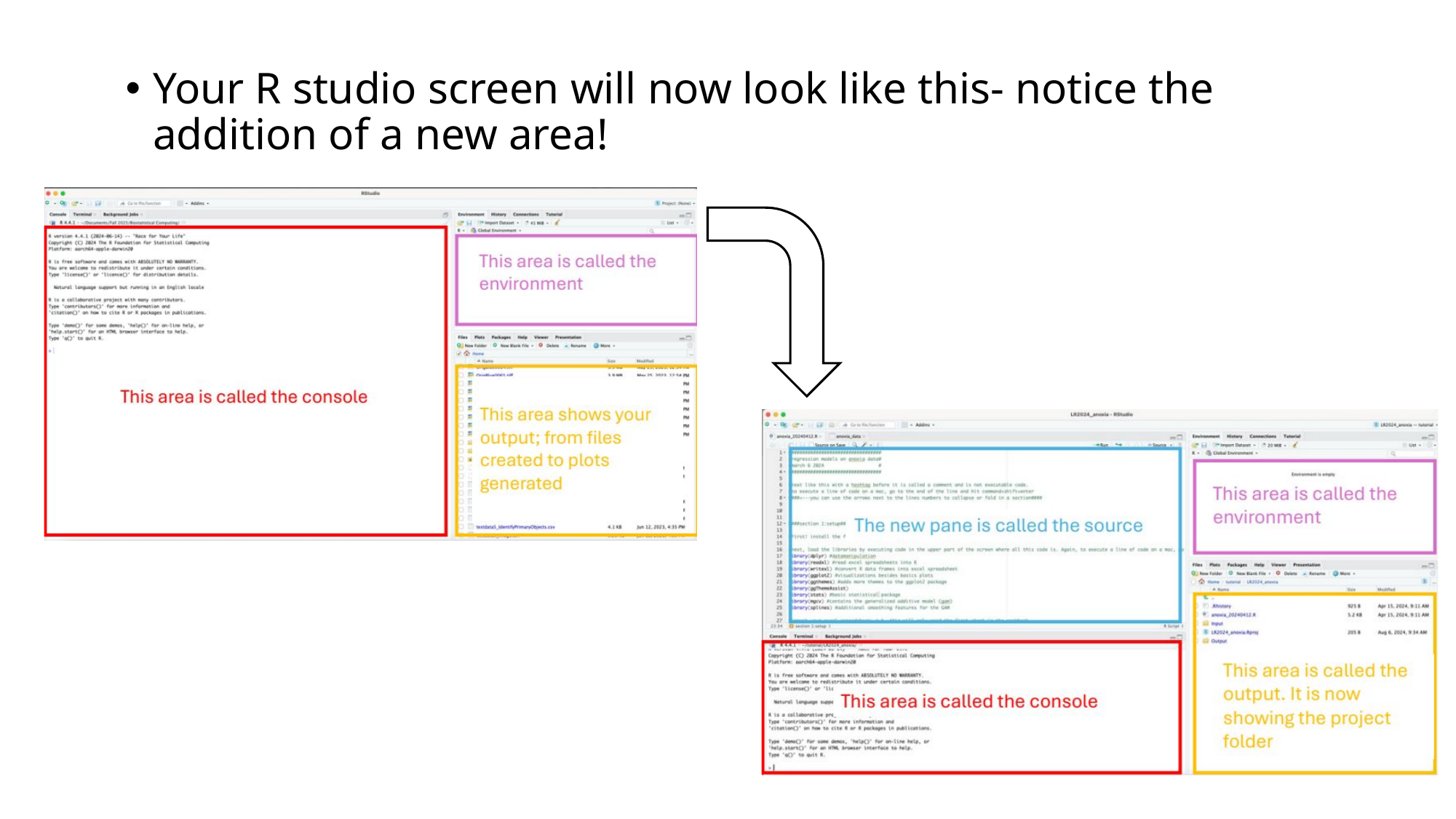

Your R studio screen will now look like this- notice the addition of a new area!

## Slide 9
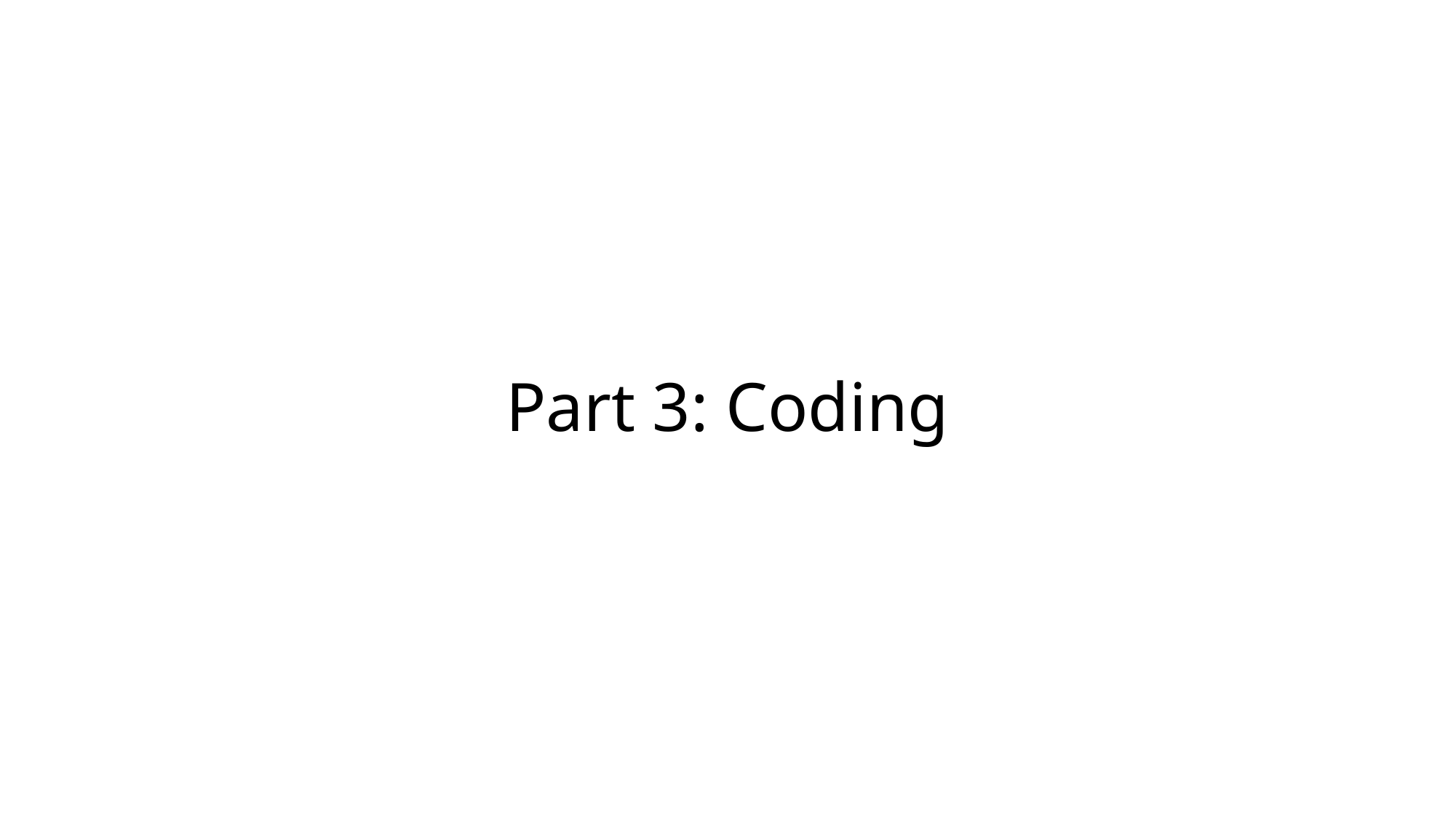

# Part 3: Coding

## Slide 10
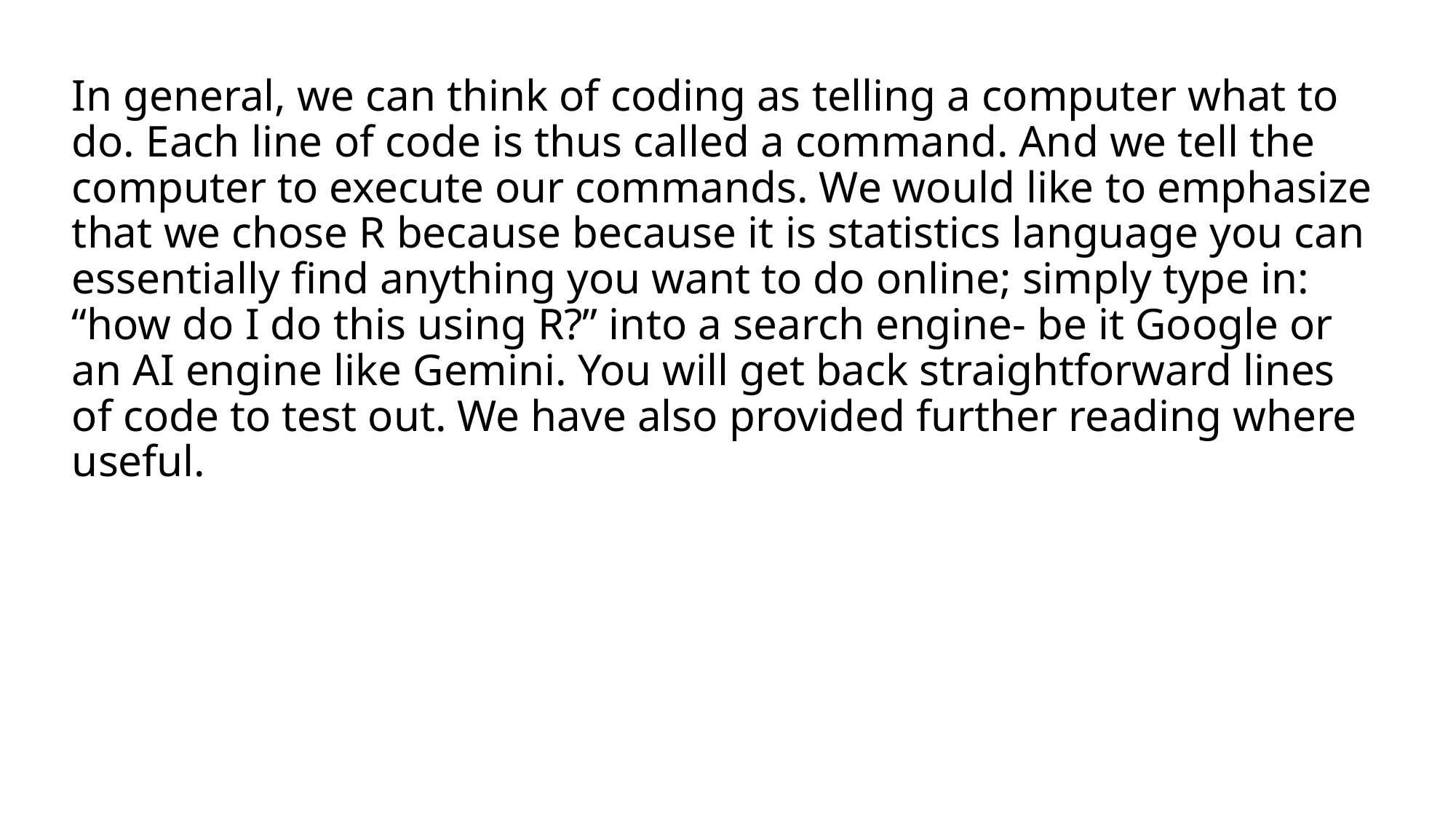

In general, we can think of coding as telling a computer what to do. Each line of code is thus called a command. And we tell the computer to execute our commands. We would like to emphasize that we chose R because because it is statistics language you can essentially find anything you want to do online; simply type in: “how do I do this using R?” into a search engine- be it Google or an AI engine like Gemini. You will get back straightforward lines of code to test out. We have also provided further reading where useful.

## Slide 11
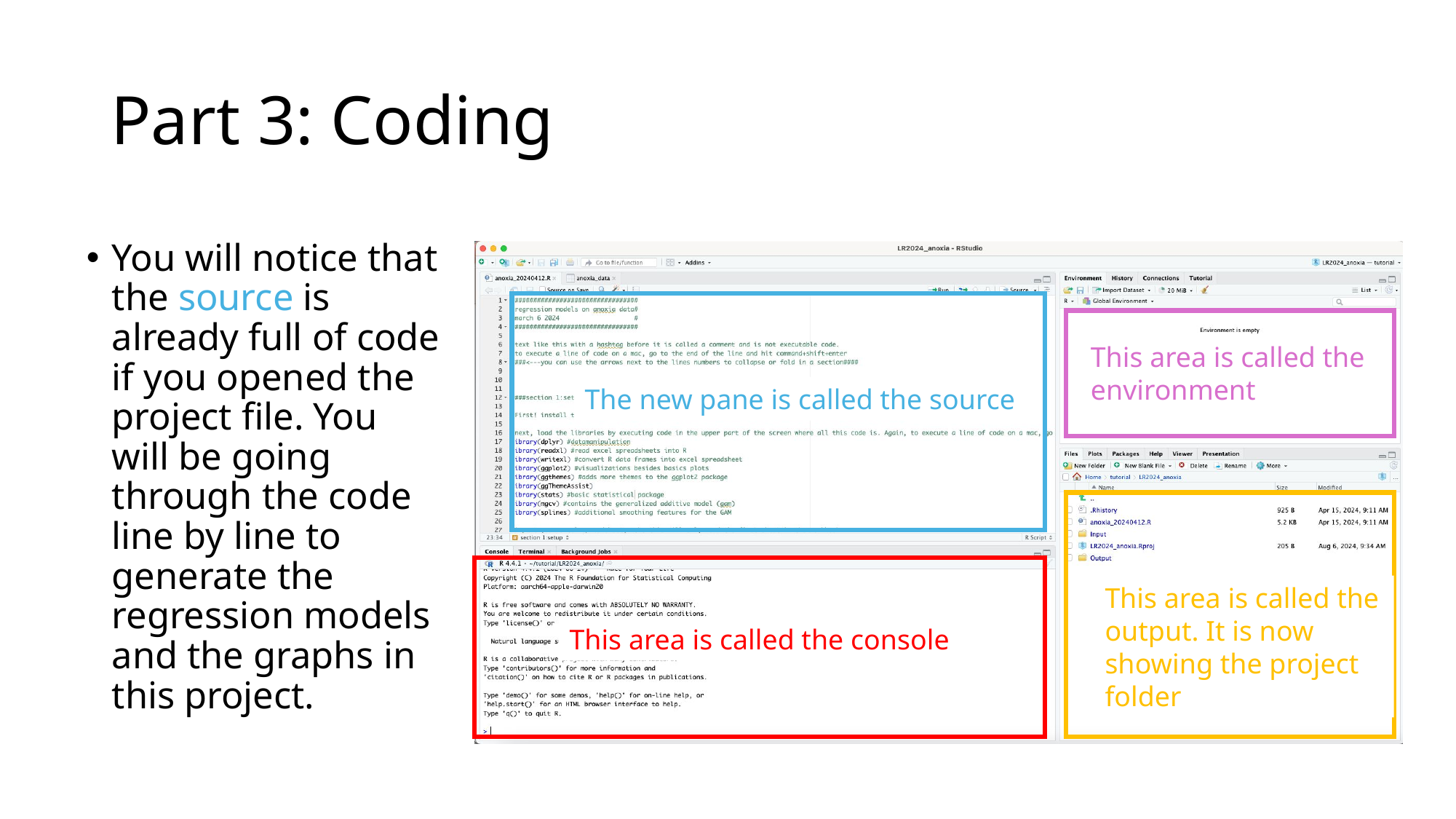

# Part 3: Coding
You will notice that the source is already full of code if you opened the project file. You will be going through the code line by line to generate the regression models and the graphs in this project.
This area is called the environment
The new pane is called the source
This area is called the output. It is now showing the project folder
This area is called the console

## Slide 12
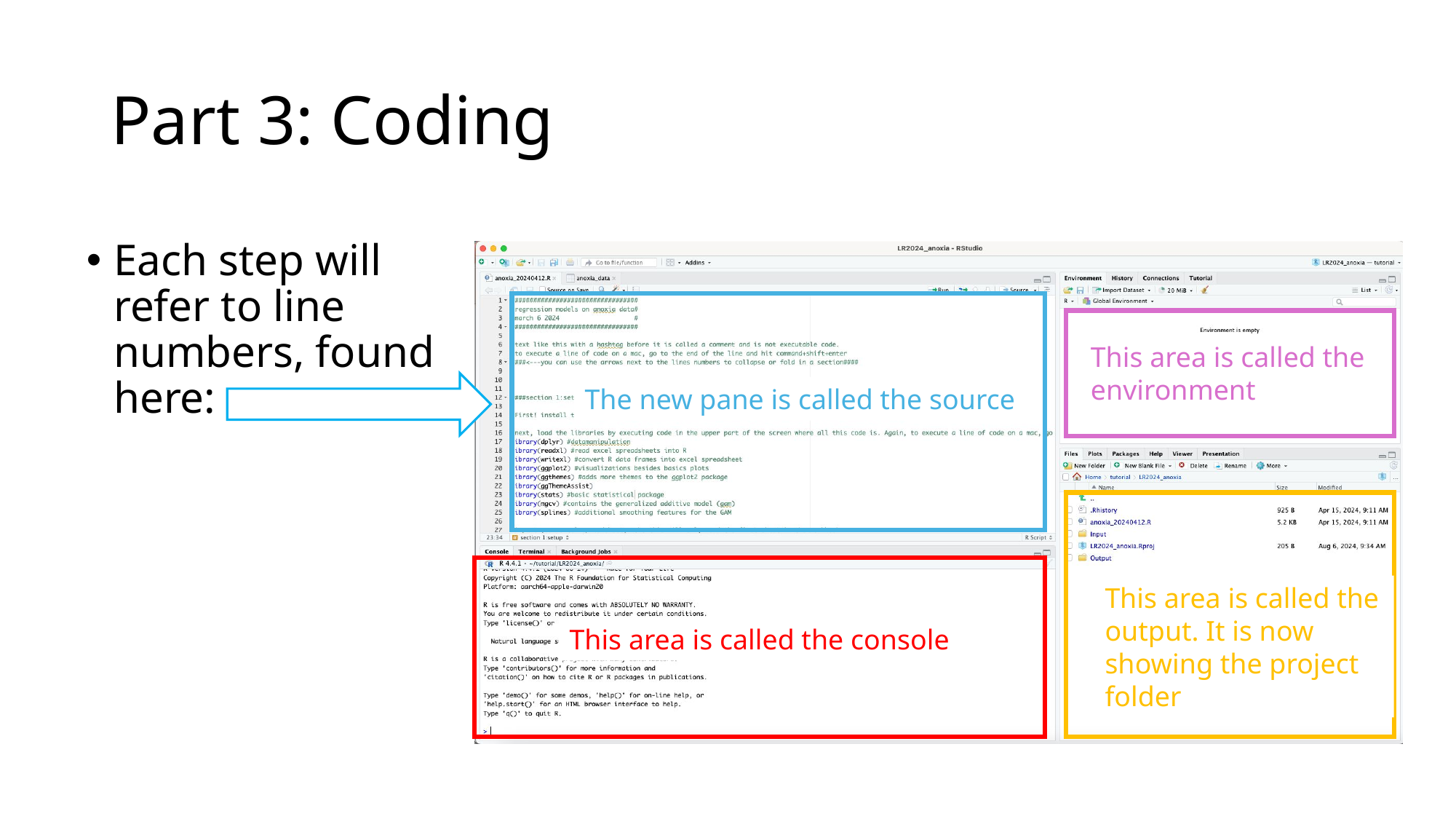

# Part 3: Coding
Each step will refer to line numbers, found here:
This area is called the environment
The new pane is called the source
This area is called the output. It is now showing the project folder
This area is called the console

## Slide 13
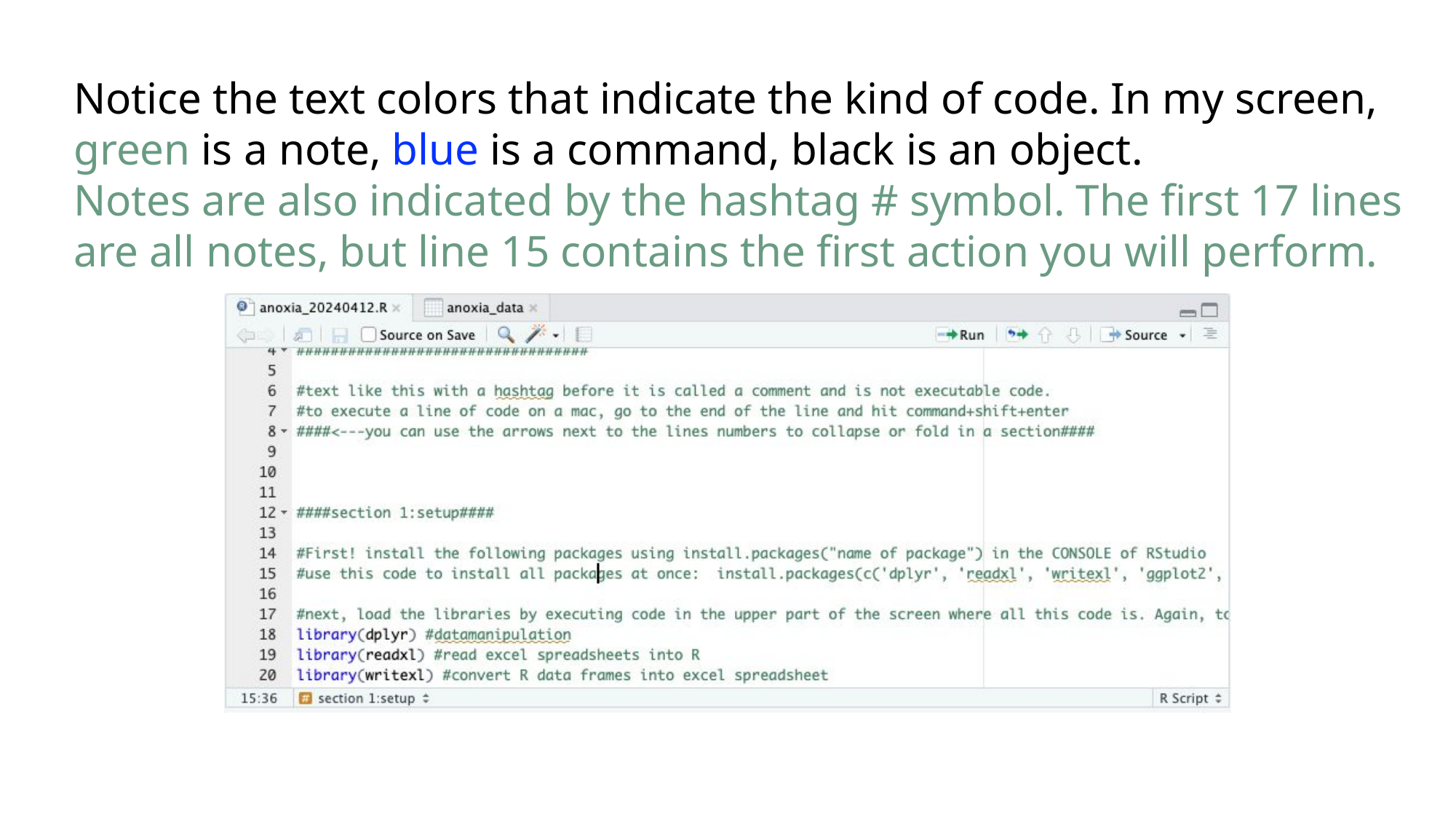

Notice the text colors that indicate the kind of code. In my screen, green is a note, blue is a command, black is an object.
Notes are also indicated by the hashtag # symbol. The first 17 lines are all notes, but line 15 contains the first action you will perform.

## Slide 14
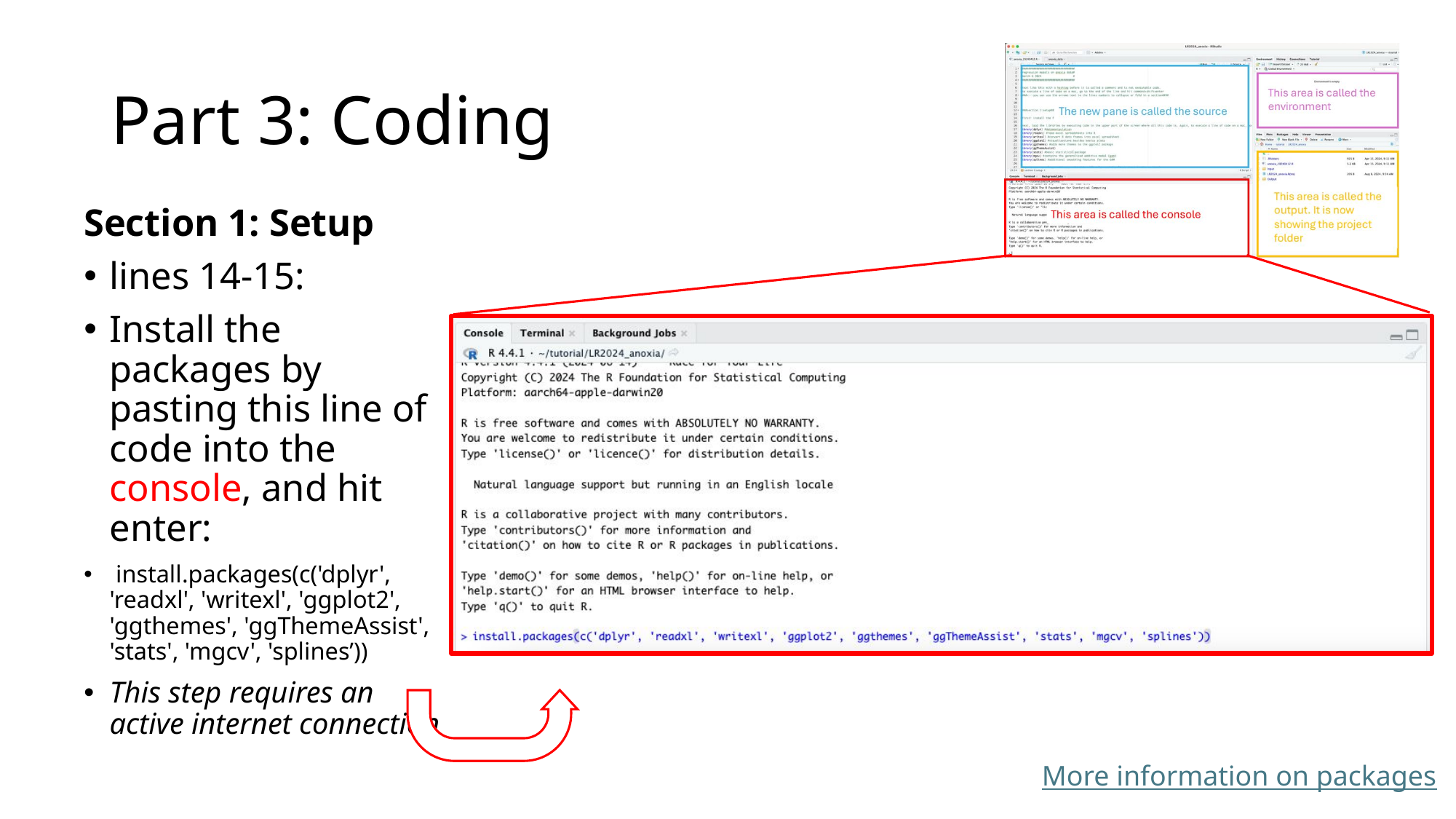

# Part 3: Coding
Section 1: Setup
lines 14-15:
Install the packages by pasting this line of code into the console, and hit enter:
 install.packages(c('dplyr', 'readxl', 'writexl', 'ggplot2', 'ggthemes', 'ggThemeAssist', 'stats', 'mgcv', 'splines’))
This step requires an active internet connection
More information on packages

## Slide 15
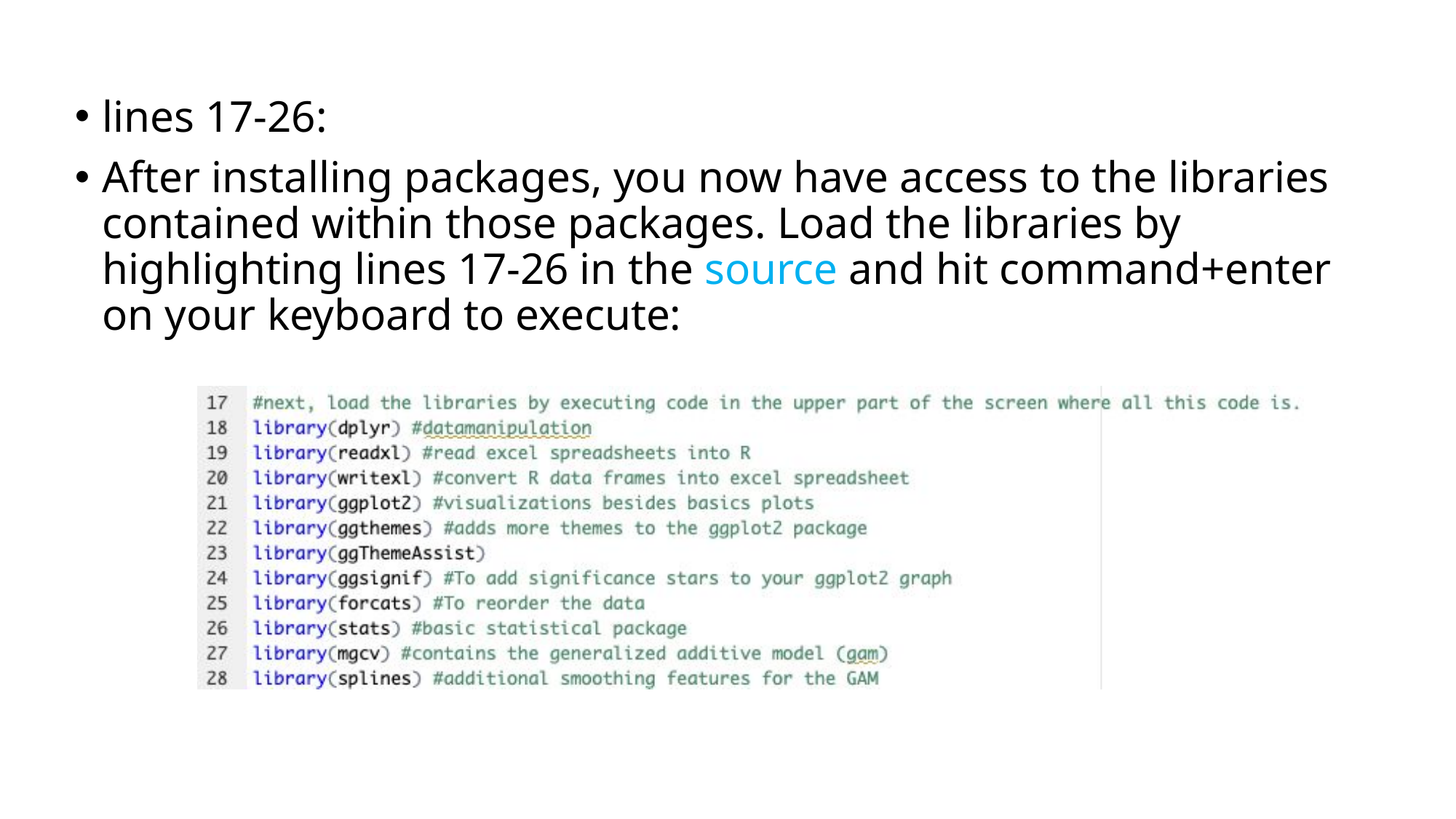

lines 17-26:
After installing packages, you now have access to the libraries contained within those packages. Load the libraries by highlighting lines 17-26 in the source and hit command+enter on your keyboard to execute:

## Slide 16
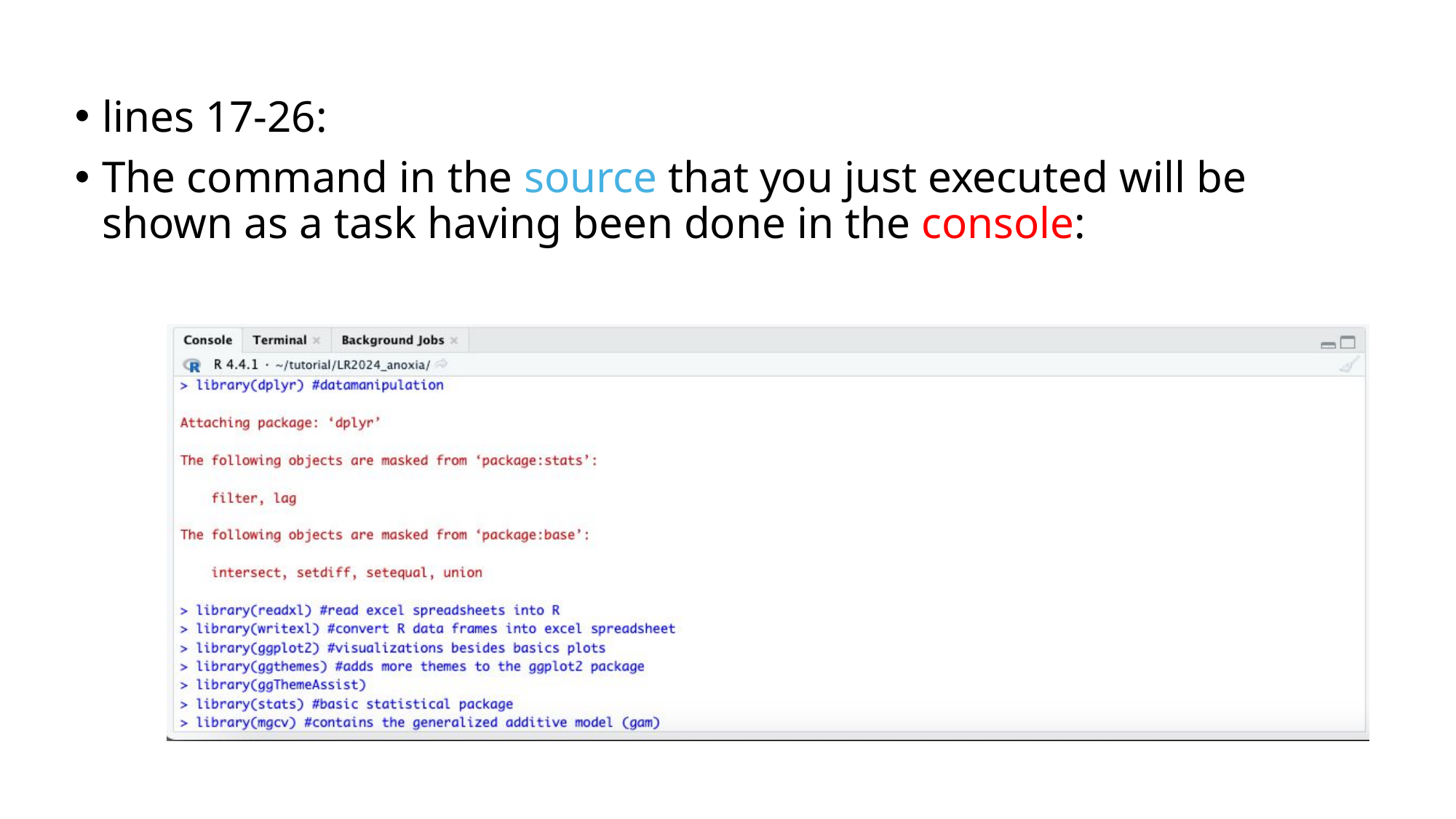

lines 17-26:
The command in the source that you just executed will be shown as a task having been done in the console:

## Slide 17
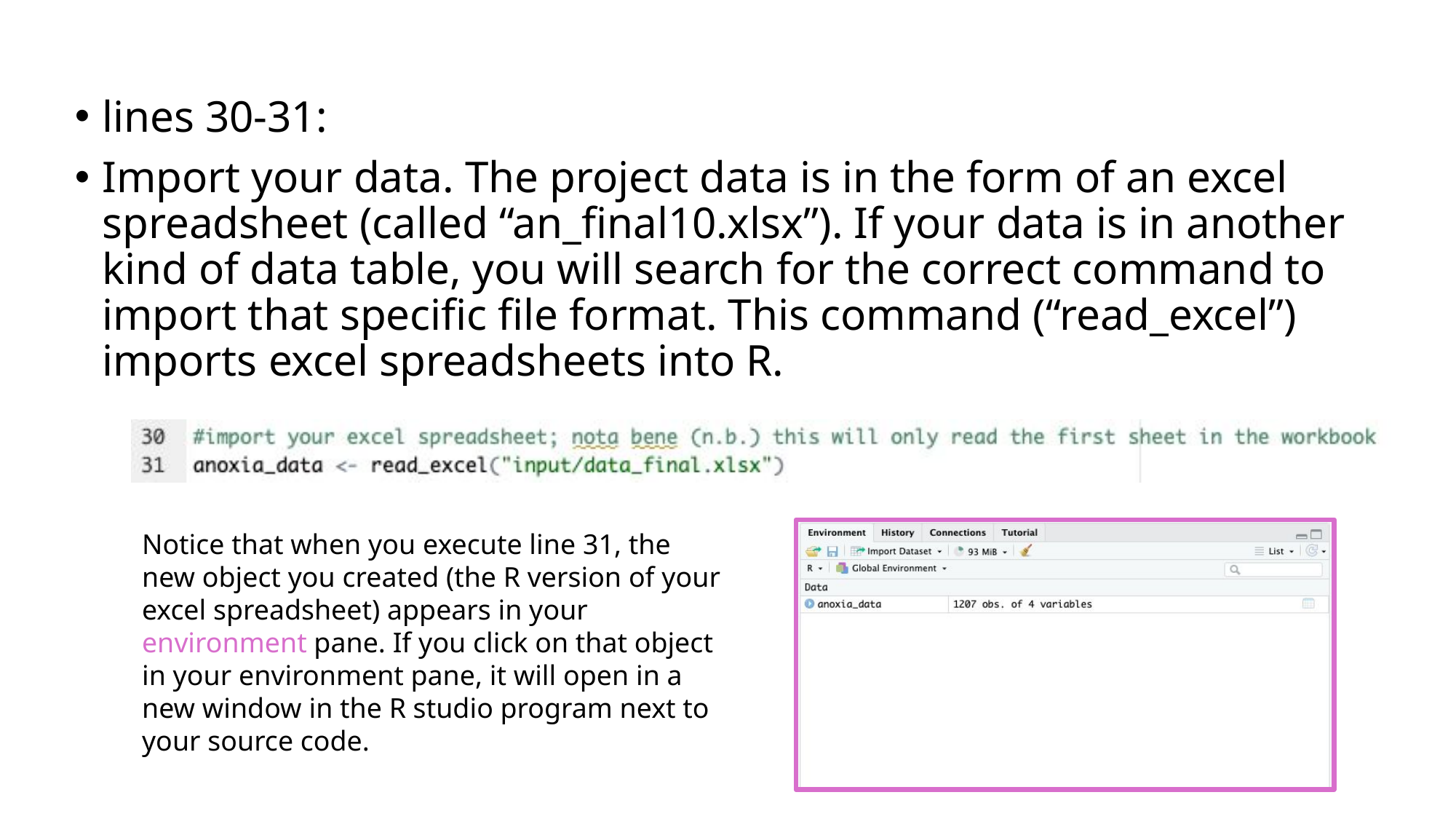

lines 30-31:
Import your data. The project data is in the form of an excel spreadsheet (called “an_final10.xlsx”). If your data is in another kind of data table, you will search for the correct command to import that specific file format. This command (“read_excel”) imports excel spreadsheets into R.
Notice that when you execute line 31, the new object you created (the R version of your excel spreadsheet) appears in your environment pane. If you click on that object in your environment pane, it will open in a new window in the R studio program next to your source code.

## Slide 18
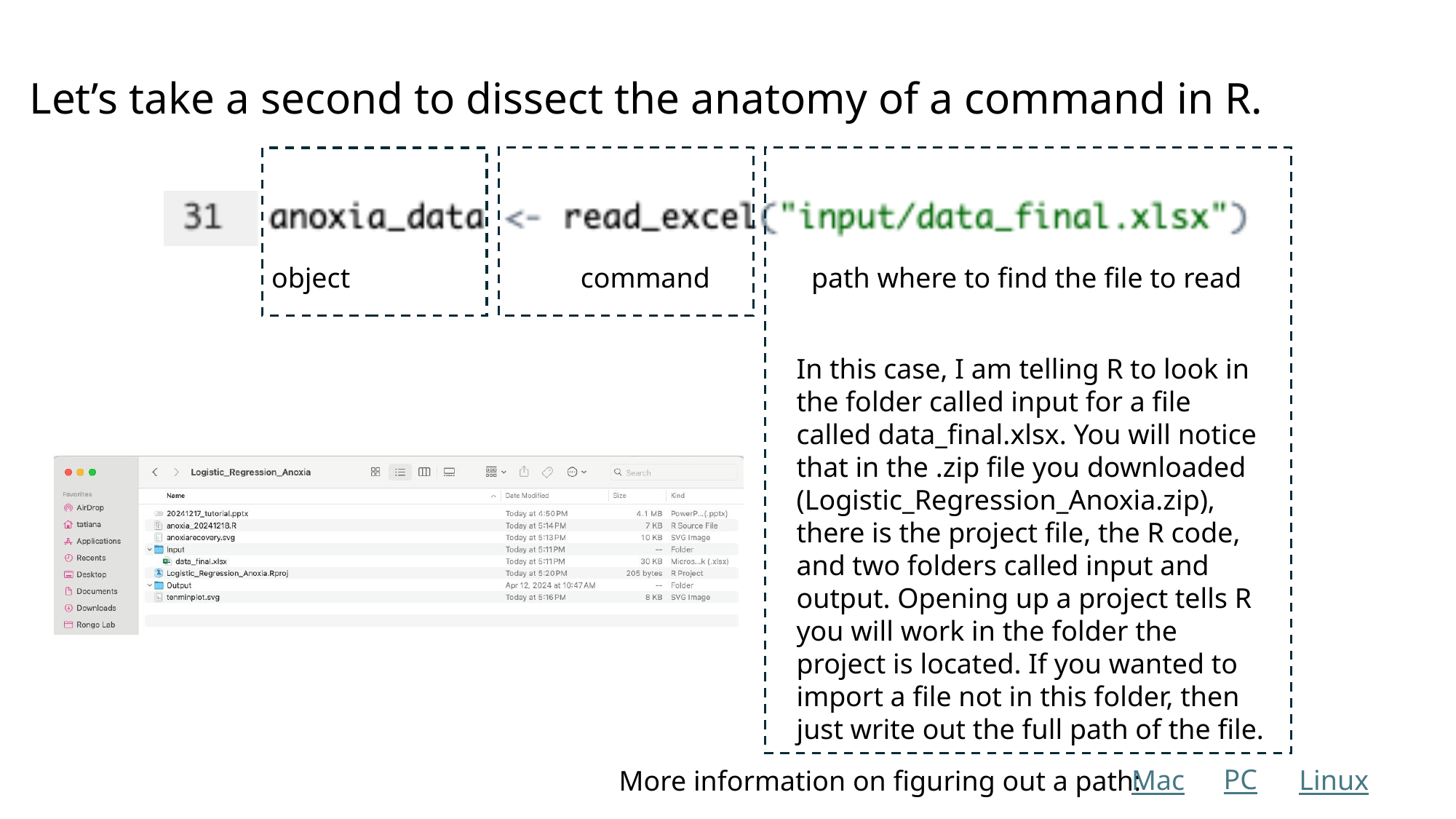

Let’s take a second to dissect the anatomy of a command in R.
object
command
path where to find the file to read
In this case, I am telling R to look in the folder called input for a file called data_final.xlsx. You will notice that in the .zip file you downloaded (Logistic_Regression_Anoxia.zip), there is the project file, the R code, and two folders called input and output. Opening up a project tells R you will work in the folder the project is located. If you wanted to import a file not in this folder, then just write out the full path of the file.
PC
Mac
Linux
More information on figuring out a path:

## Slide 19
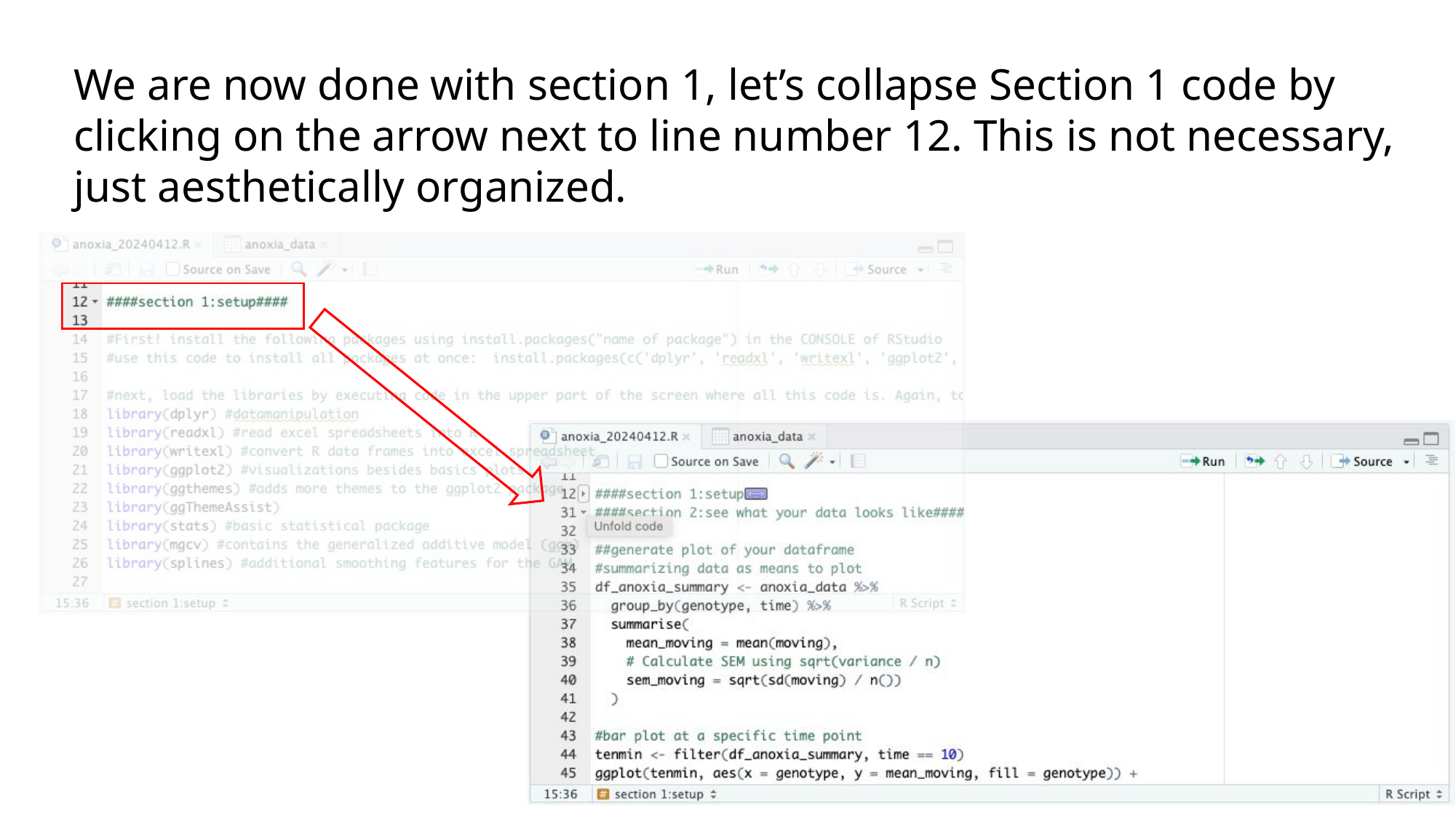

We are now done with section 1, let’s collapse Section 1 code by clicking on the arrow next to line number 12. This is not necessary, just aesthetically organized.

## Slide 20
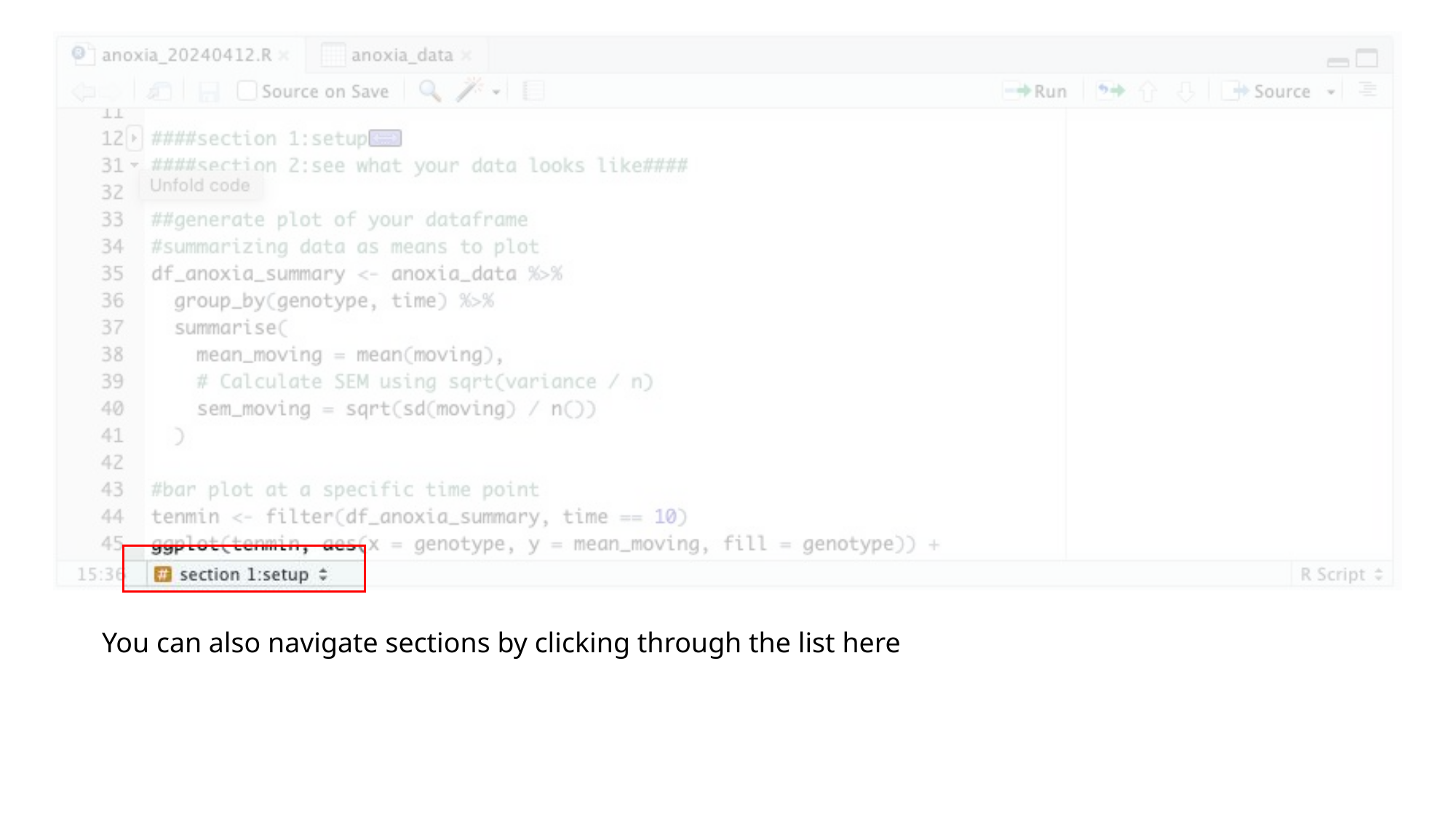

You can also navigate sections by clicking through the list here

## Slide 21
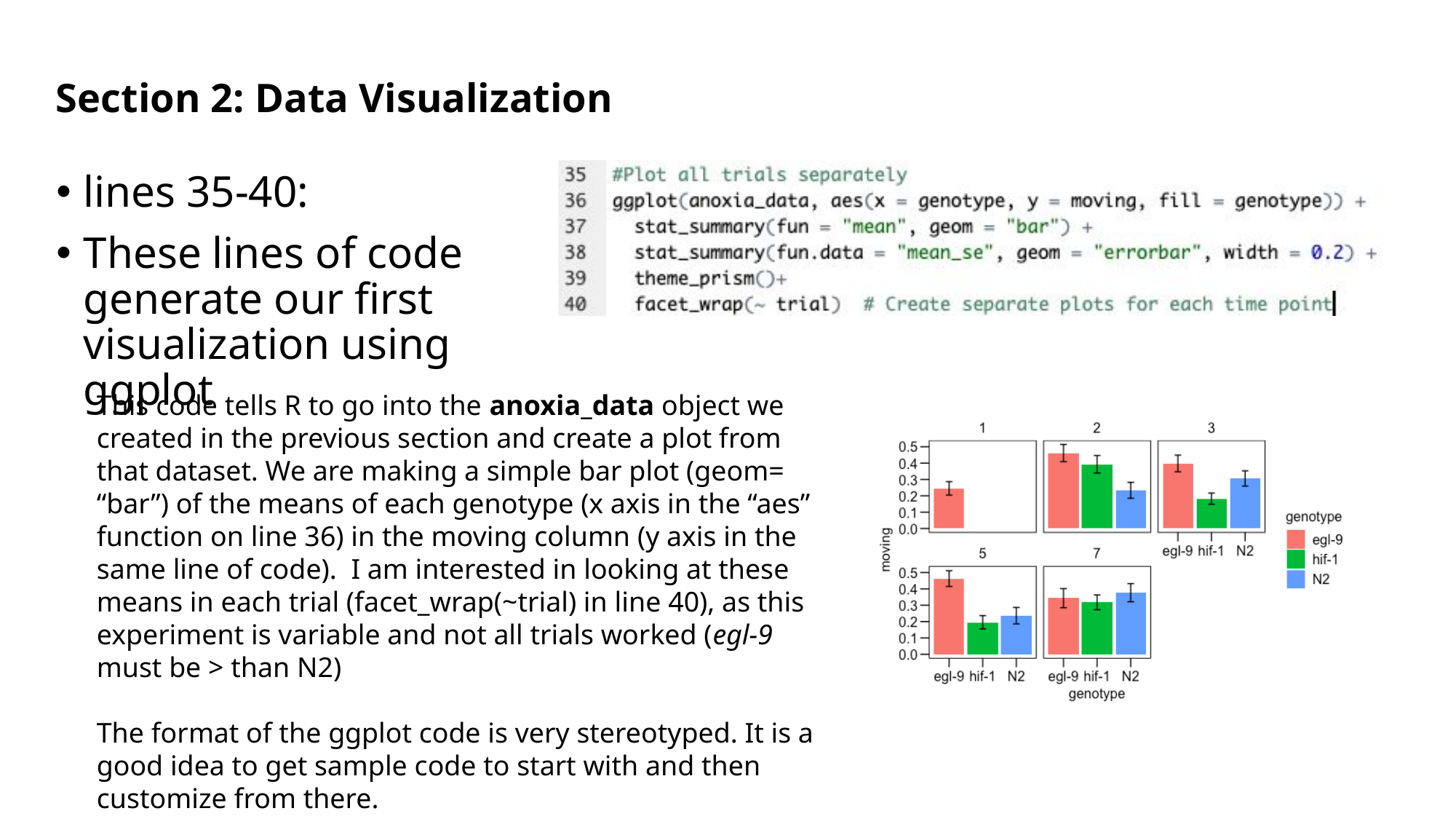

Section 2: Data Visualization
lines 35-40:
These lines of code generate our first visualization using ggplot
This code tells R to go into the anoxia_data object we created in the previous section and create a plot from that dataset. We are making a simple bar plot (geom= “bar”) of the means of each genotype (x axis in the “aes” function on line 36) in the moving column (y axis in the same line of code). I am interested in looking at these means in each trial (facet_wrap(~trial) in line 40), as this experiment is variable and not all trials worked (egl-9 must be > than N2)
The format of the ggplot code is very stereotyped. It is a good idea to get sample code to start with and then customize from there.

## Slide 22
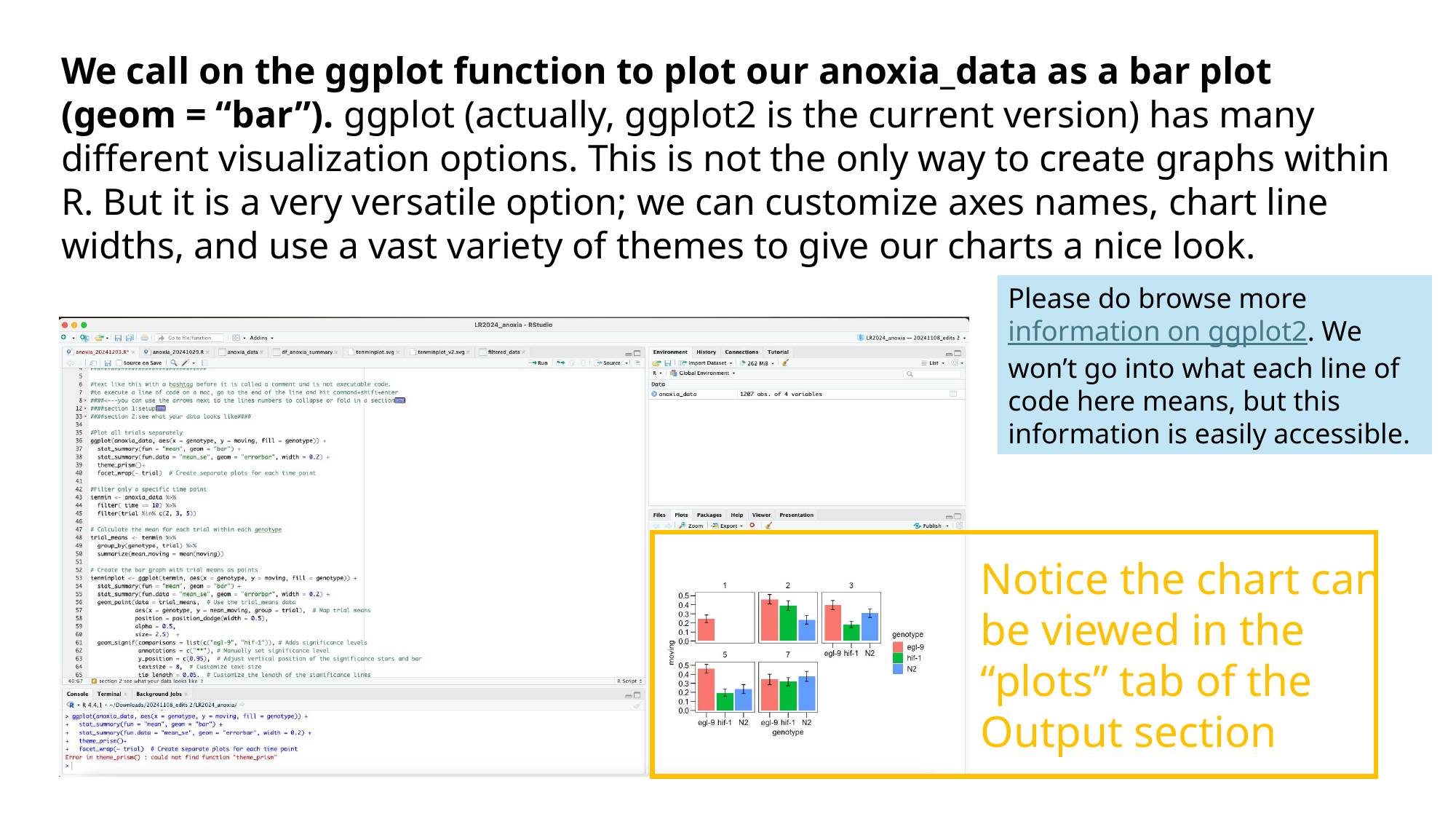

We call on the ggplot function to plot our anoxia_data as a bar plot (geom = “bar”). ggplot (actually, ggplot2 is the current version) has many different visualization options. This is not the only way to create graphs within R. But it is a very versatile option; we can customize axes names, chart line widths, and use a vast variety of themes to give our charts a nice look.
Please do browse more information on ggplot2. We won’t go into what each line of code here means, but this information is easily accessible.
Notice the chart can be viewed in the “plots” tab of the Output section

## Slide 23
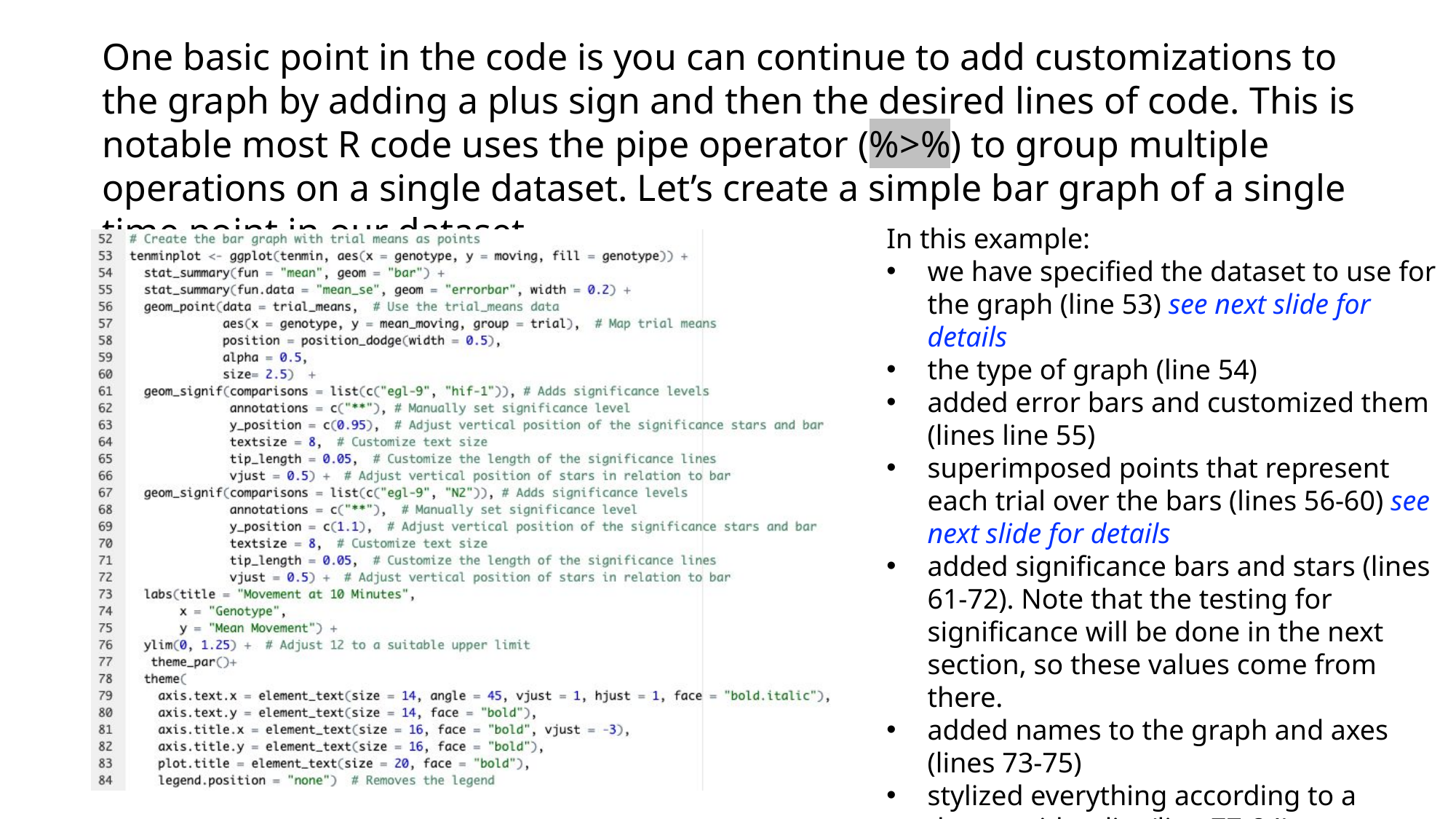

One basic point in the code is you can continue to add customizations to the graph by adding a plus sign and then the desired lines of code. This is notable most R code uses the pipe operator (%>%) to group multiple operations on a single dataset. Let’s create a simple bar graph of a single time point in our dataset.
In this example:
we have specified the dataset to use for the graph (line 53) see next slide for details
the type of graph (line 54)
added error bars and customized them (lines line 55)
superimposed points that represent each trial over the bars (lines 56-60) see next slide for details
added significance bars and stars (lines 61-72). Note that the testing for significance will be done in the next section, so these values come from there.
added names to the graph and axes (lines 73-75)
stylized everything according to a theme with edits (line 77-84).

## Slide 24
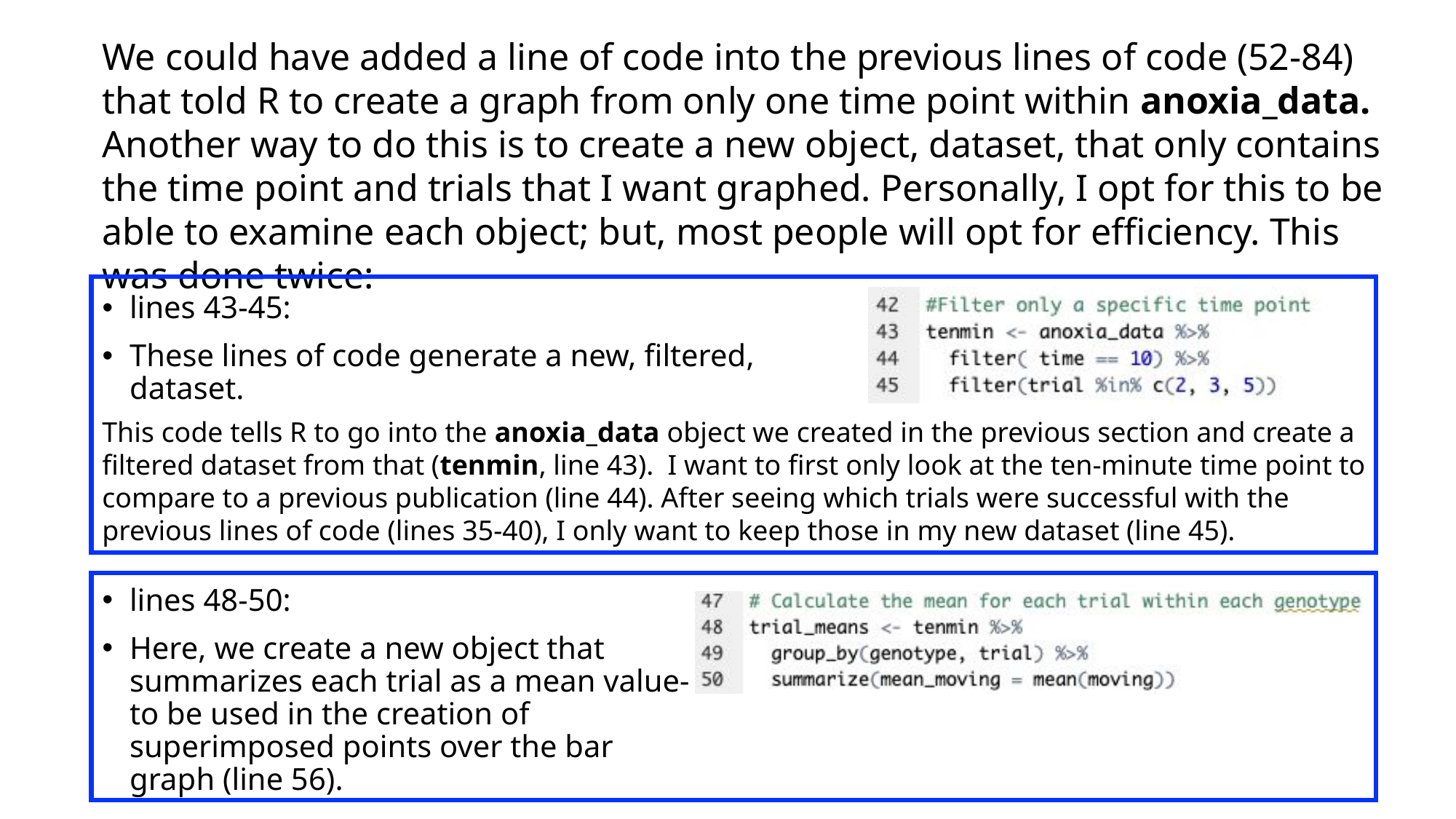

We could have added a line of code into the previous lines of code (52-84) that told R to create a graph from only one time point within anoxia_data. Another way to do this is to create a new object, dataset, that only contains the time point and trials that I want graphed. Personally, I opt for this to be able to examine each object; but, most people will opt for efficiency. This was done twice:
lines 43-45:
These lines of code generate a new, filtered, dataset.
This code tells R to go into the anoxia_data object we created in the previous section and create a filtered dataset from that (tenmin, line 43). I want to first only look at the ten-minute time point to compare to a previous publication (line 44). After seeing which trials were successful with the previous lines of code (lines 35-40), I only want to keep those in my new dataset (line 45).
lines 48-50:
Here, we create a new object that summarizes each trial as a mean value- to be used in the creation of superimposed points over the bar graph (line 56).

## Slide 25
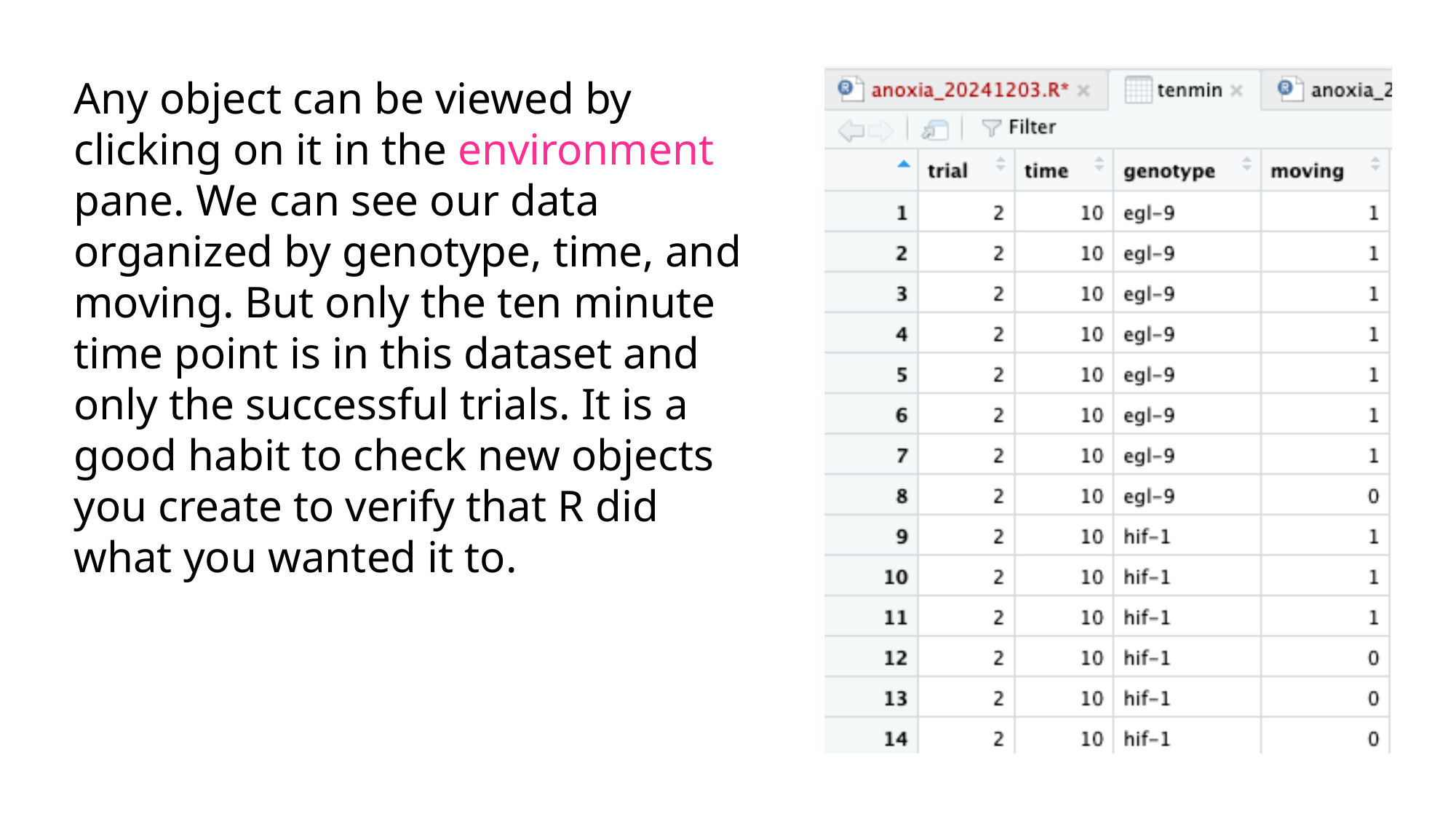

Any object can be viewed by clicking on it in the environment pane. We can see our data organized by genotype, time, and moving. But only the ten minute time point is in this dataset and only the successful trials. It is a good habit to check new objects you create to verify that R did what you wanted it to.

## Slide 26
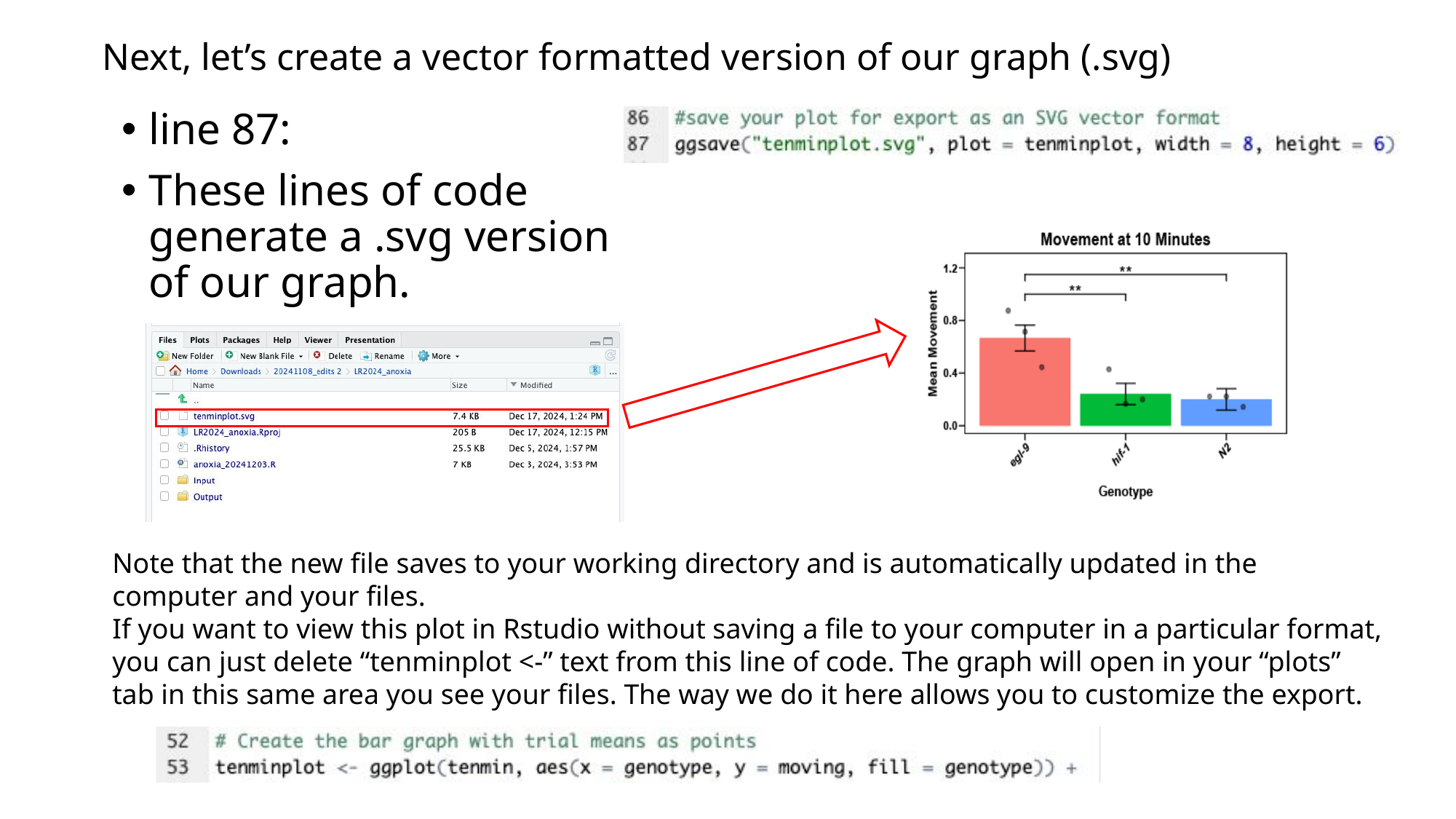

Next, let’s create a vector formatted version of our graph (.svg)
line 87:
These lines of code generate a .svg version of our graph.
Note that the new file saves to your working directory and is automatically updated in the computer and your files.
If you want to view this plot in Rstudio without saving a file to your computer in a particular format, you can just delete “tenminplot <-” text from this line of code. The graph will open in your “plots” tab in this same area you see your files. The way we do it here allows you to customize the export.

## Slide 27
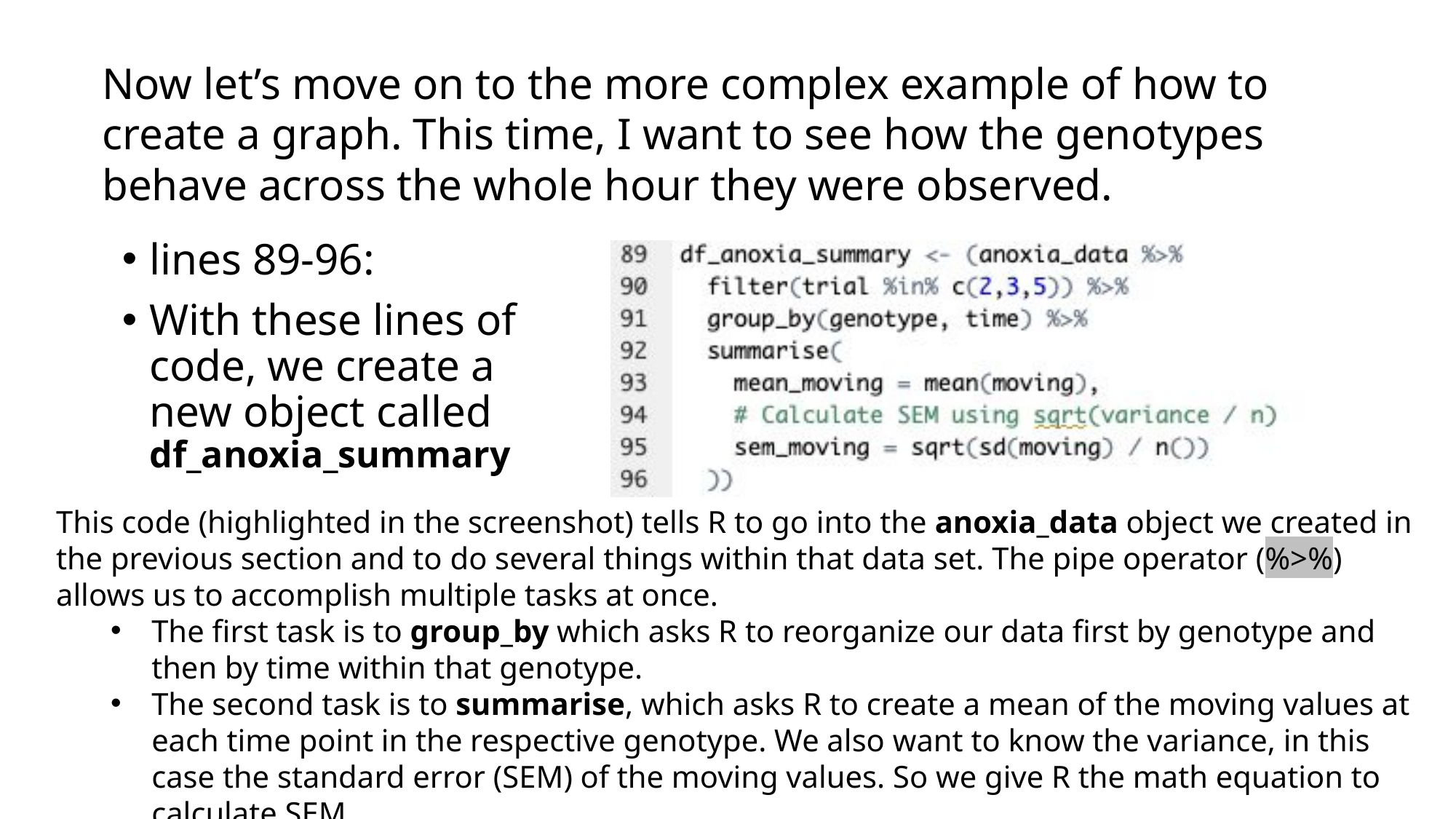

Now let’s move on to the more complex example of how to create a graph. This time, I want to see how the genotypes behave across the whole hour they were observed.
lines 89-96:
With these lines of code, we create a new object called df_anoxia_summary
This code (highlighted in the screenshot) tells R to go into the anoxia_data object we created in the previous section and to do several things within that data set. The pipe operator (%>%) allows us to accomplish multiple tasks at once.
The first task is to group_by which asks R to reorganize our data first by genotype and then by time within that genotype.
The second task is to summarise, which asks R to create a mean of the moving values at each time point in the respective genotype. We also want to know the variance, in this case the standard error (SEM) of the moving values. So we give R the math equation to calculate SEM.

## Slide 28
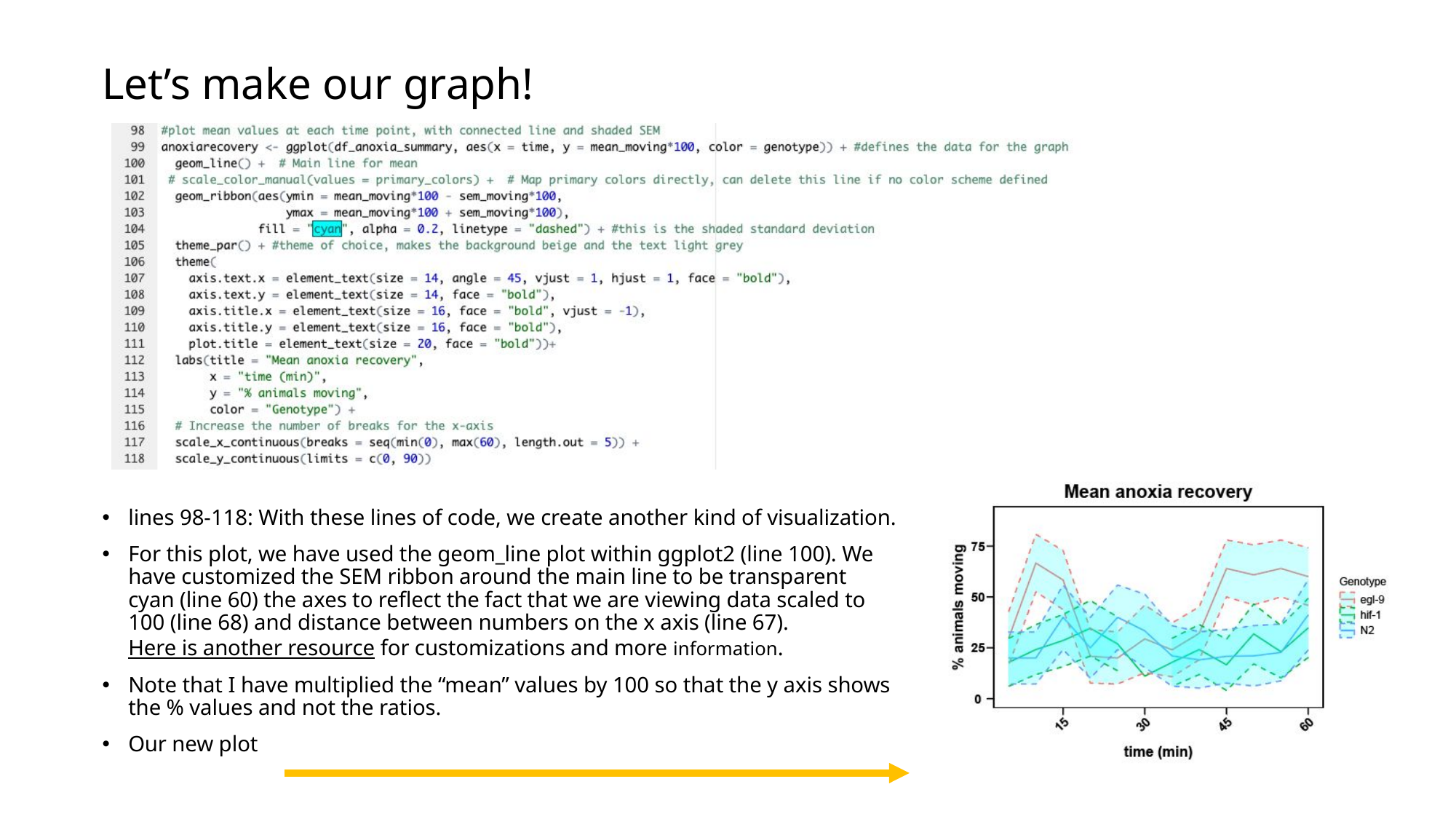

Let’s make our graph!
lines 98-118: With these lines of code, we create another kind of visualization.
For this plot, we have used the geom_line plot within ggplot2 (line 100). We have customized the SEM ribbon around the main line to be transparent cyan (line 60) the axes to reflect the fact that we are viewing data scaled to 100 (line 68) and distance between numbers on the x axis (line 67). Here is another resource for customizations and more information.
Note that I have multiplied the “mean” values by 100 so that the y axis shows the % values and not the ratios.
Our new plot

## Slide 29
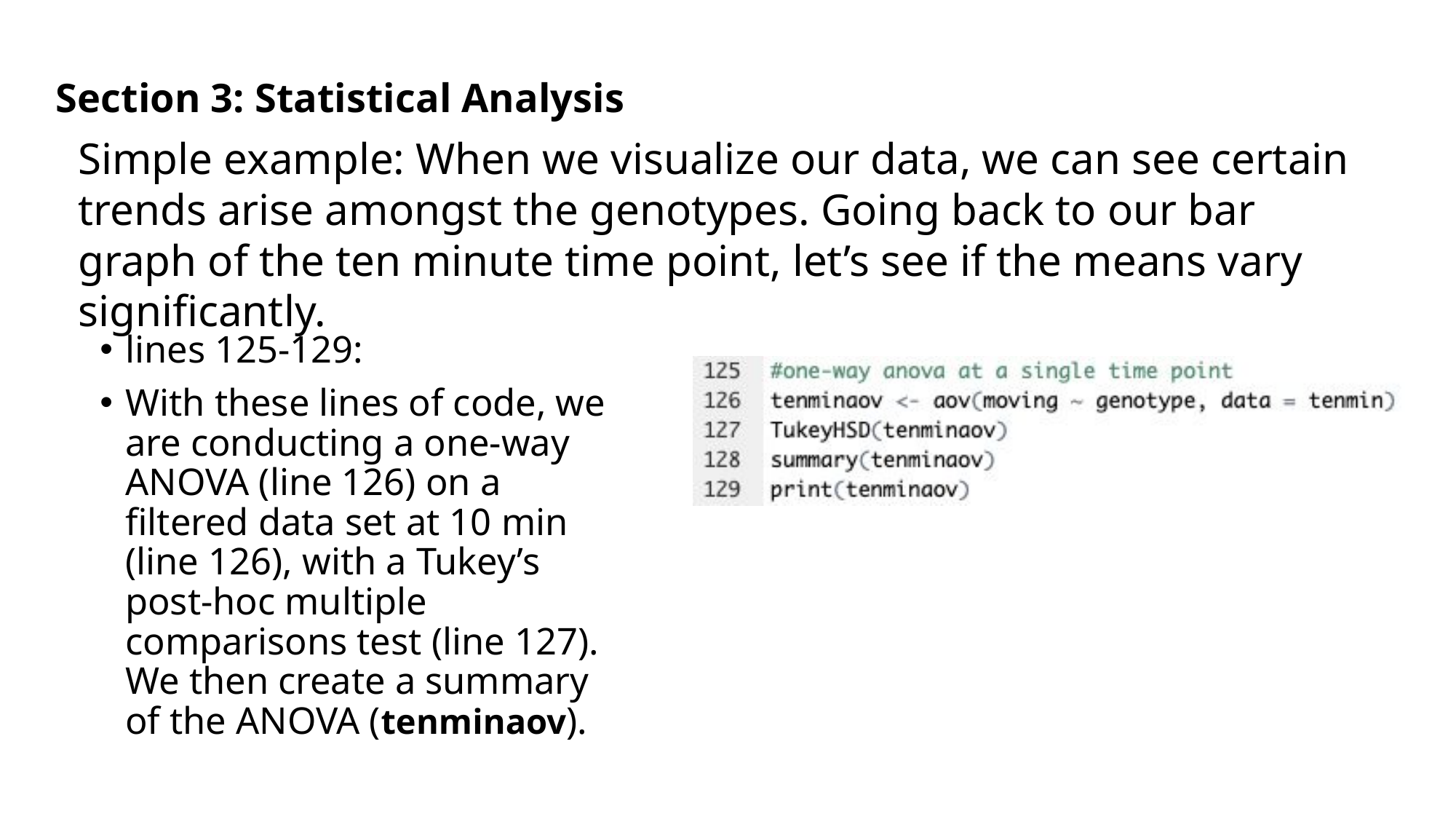

Section 3: Statistical Analysis
Simple example: When we visualize our data, we can see certain trends arise amongst the genotypes. Going back to our bar graph of the ten minute time point, let’s see if the means vary significantly.
lines 125-129:
With these lines of code, we are conducting a one-way ANOVA (line 126) on a filtered data set at 10 min (line 126), with a Tukey’s post-hoc multiple comparisons test (line 127). We then create a summary of the ANOVA (tenminaov).

## Slide 30
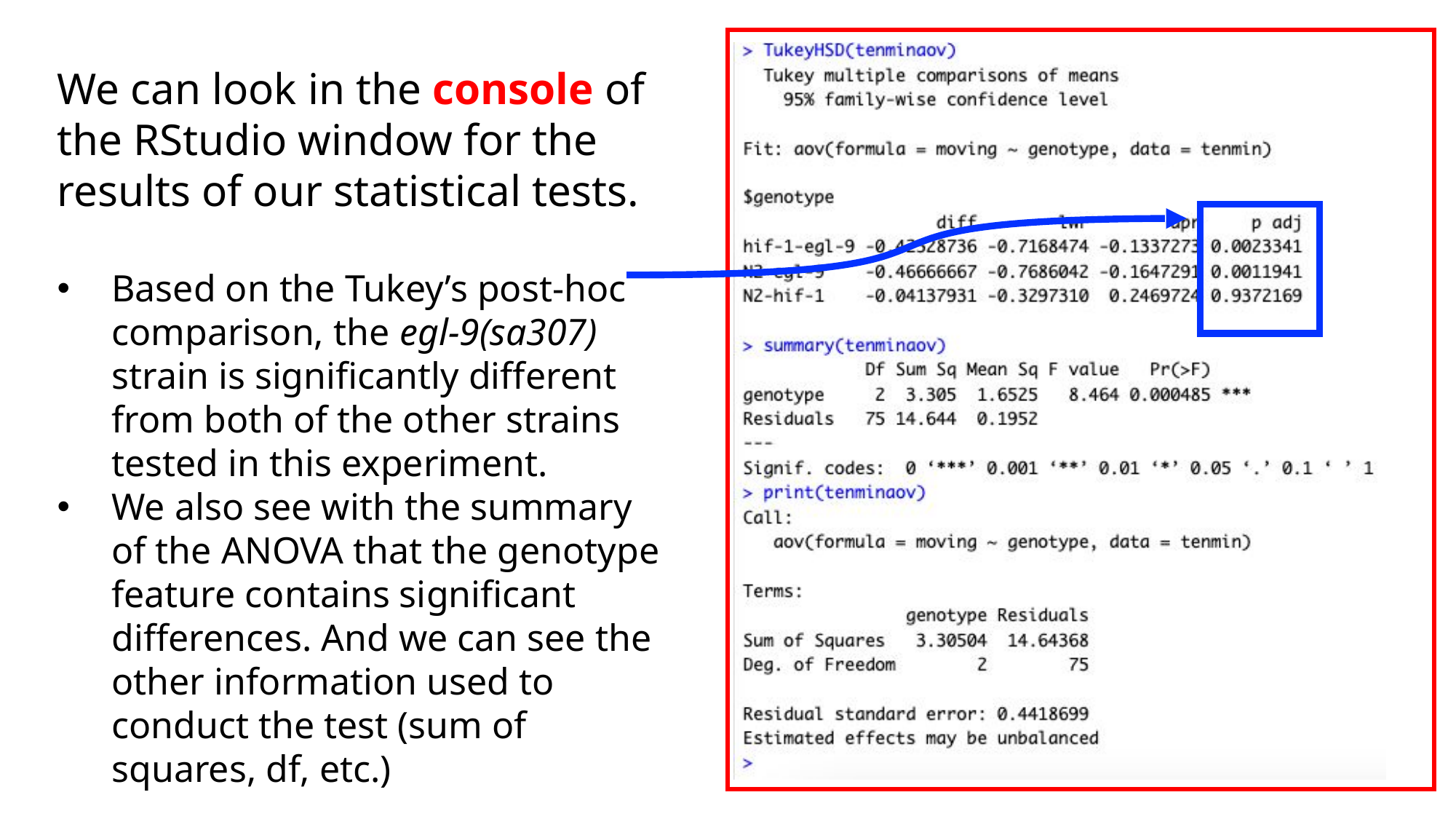

We can look in the console of the RStudio window for the results of our statistical tests.
Based on the Tukey’s post-hoc comparison, the egl-9(sa307) strain is significantly different from both of the other strains tested in this experiment.
We also see with the summary of the ANOVA that the genotype feature contains significant differences. And we can see the other information used to conduct the test (sum of squares, df, etc.)

## Slide 31
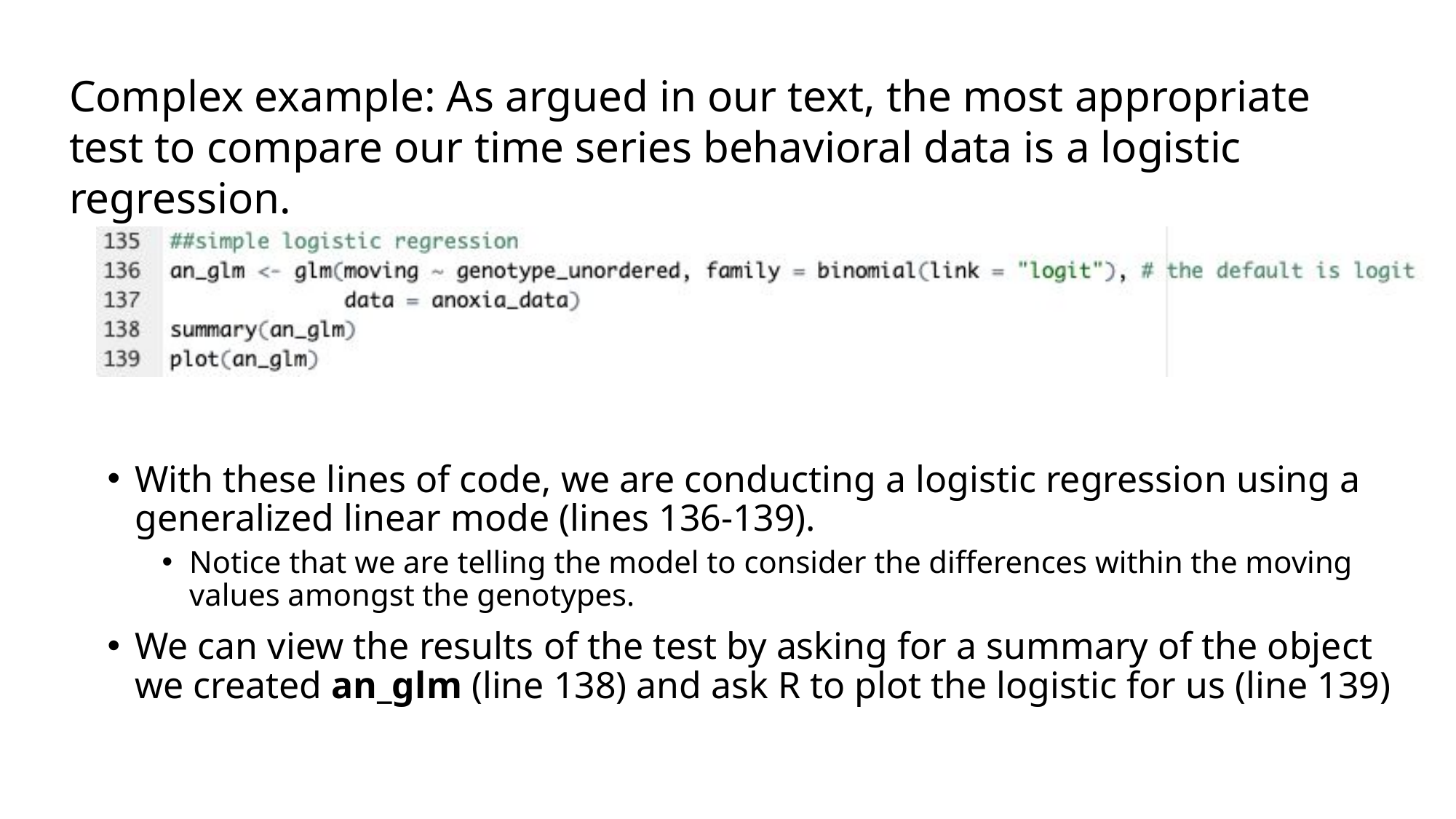

Complex example: As argued in our text, the most appropriate test to compare our time series behavioral data is a logistic regression.
With these lines of code, we are conducting a logistic regression using a generalized linear mode (lines 136-139).
Notice that we are telling the model to consider the differences within the moving values amongst the genotypes.
We can view the results of the test by asking for a summary of the object we created an_glm (line 138) and ask R to plot the logistic for us (line 139)

## Slide 32
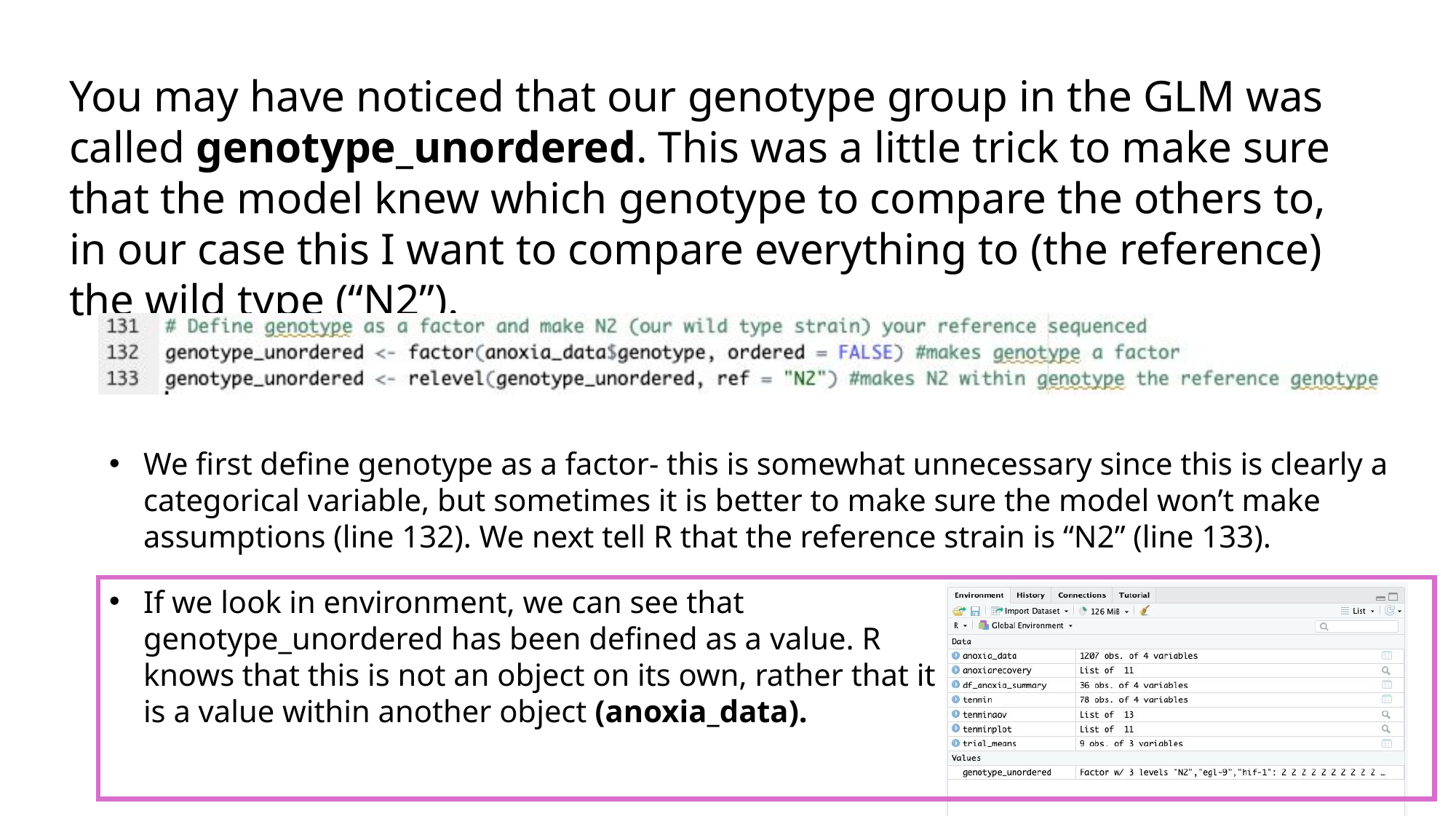

You may have noticed that our genotype group in the GLM was called genotype_unordered. This was a little trick to make sure that the model knew which genotype to compare the others to, in our case this I want to compare everything to (the reference) the wild type (“N2”).
We first define genotype as a factor- this is somewhat unnecessary since this is clearly a categorical variable, but sometimes it is better to make sure the model won’t make assumptions (line 132). We next tell R that the reference strain is “N2” (line 133).
If we look in environment, we can see that genotype_unordered has been defined as a value. R knows that this is not an object on its own, rather that it is a value within another object (anoxia_data).

## Slide 33
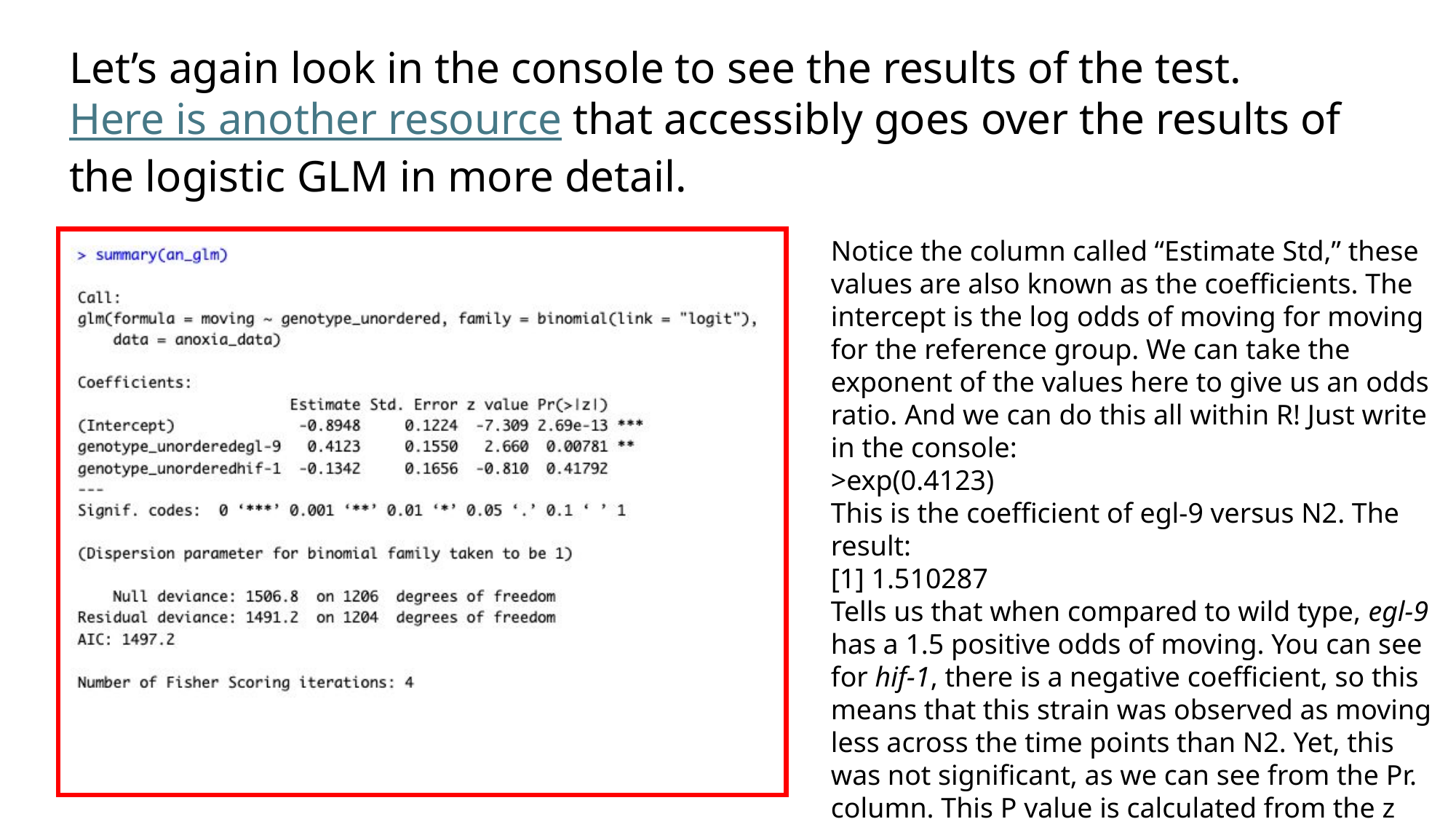

Let’s again look in the console to see the results of the test. Here is another resource that accessibly goes over the results of the logistic GLM in more detail.
Notice the column called “Estimate Std,” these values are also known as the coefficients. The intercept is the log odds of moving for moving for the reference group. We can take the exponent of the values here to give us an odds ratio. And we can do this all within R! Just write in the console:
>exp(0.4123)
This is the coefficient of egl-9 versus N2. The result:
[1] 1.510287
Tells us that when compared to wild type, egl-9 has a 1.5 positive odds of moving. You can see for hif-1, there is a negative coefficient, so this means that this strain was observed as moving less across the time points than N2. Yet, this was not significant, as we can see from the Pr. column. This P value is calculated from the z value and the ratio of the Std. Error.

## Slide 34
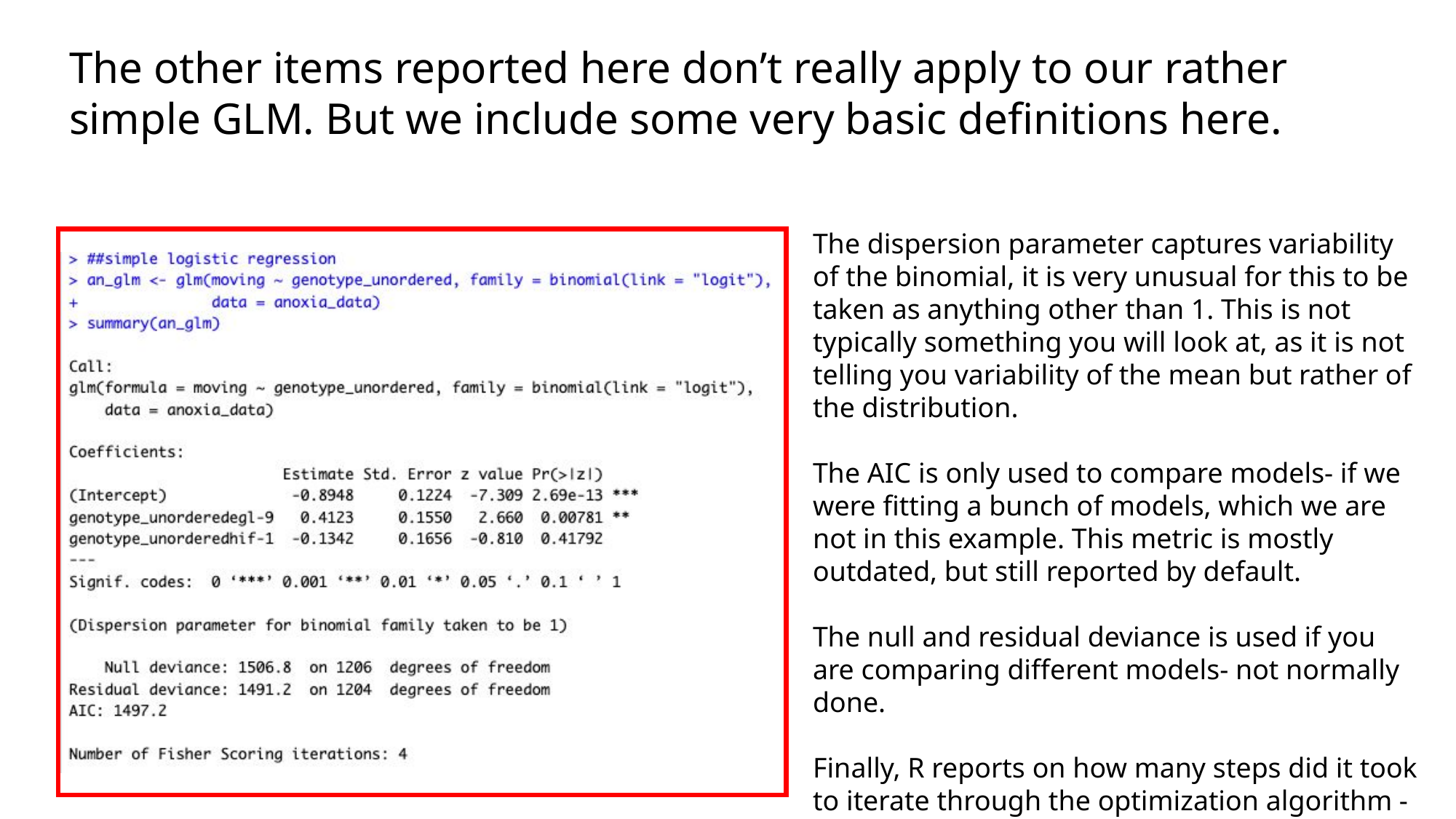

The other items reported here don’t really apply to our rather simple GLM. But we include some very basic definitions here.
The dispersion parameter captures variability of the binomial, it is very unusual for this to be taken as anything other than 1. This is not typically something you will look at, as it is not telling you variability of the mean but rather of the distribution.
The AIC is only used to compare models- if we were fitting a bunch of models, which we are not in this example. This metric is mostly outdated, but still reported by default.
The null and residual deviance is used if you are comparing different models- not normally done.
Finally, R reports on how many steps did it took to iterate through the optimization algorithm - if it didn’t converge it would tell you.

## Slide 35
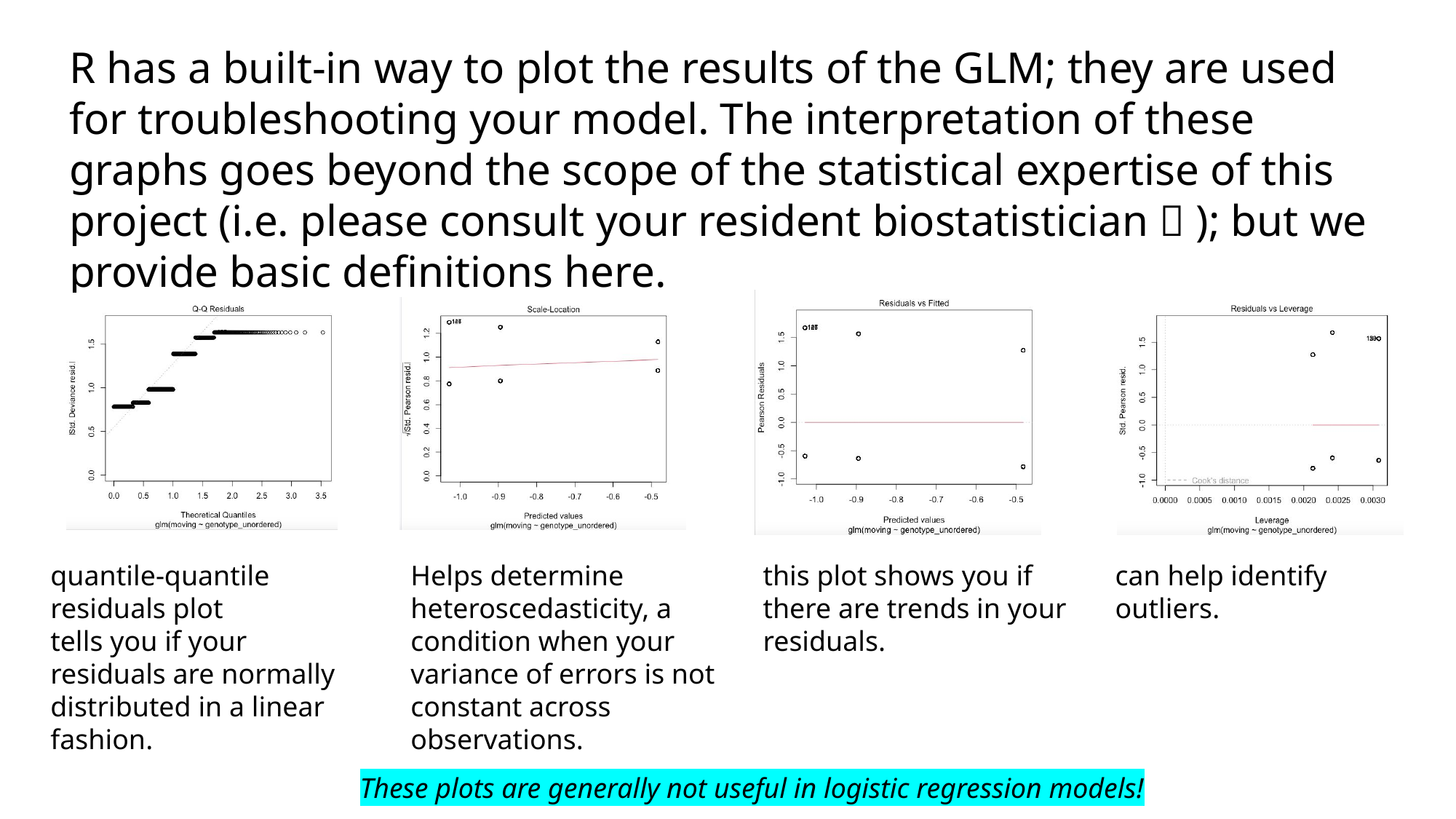

R has a built-in way to plot the results of the GLM; they are used for troubleshooting your model. The interpretation of these graphs goes beyond the scope of the statistical expertise of this project (i.e. please consult your resident biostatistician  ); but we provide basic definitions here.
quantile-quantile residuals plot
tells you if your residuals are normally distributed in a linear fashion.
Helps determine heteroscedasticity, a condition when your variance of errors is not constant across observations.
this plot shows you if there are trends in your residuals.
can help identify outliers.
These plots are generally not useful in logistic regression models!

## Slide 36
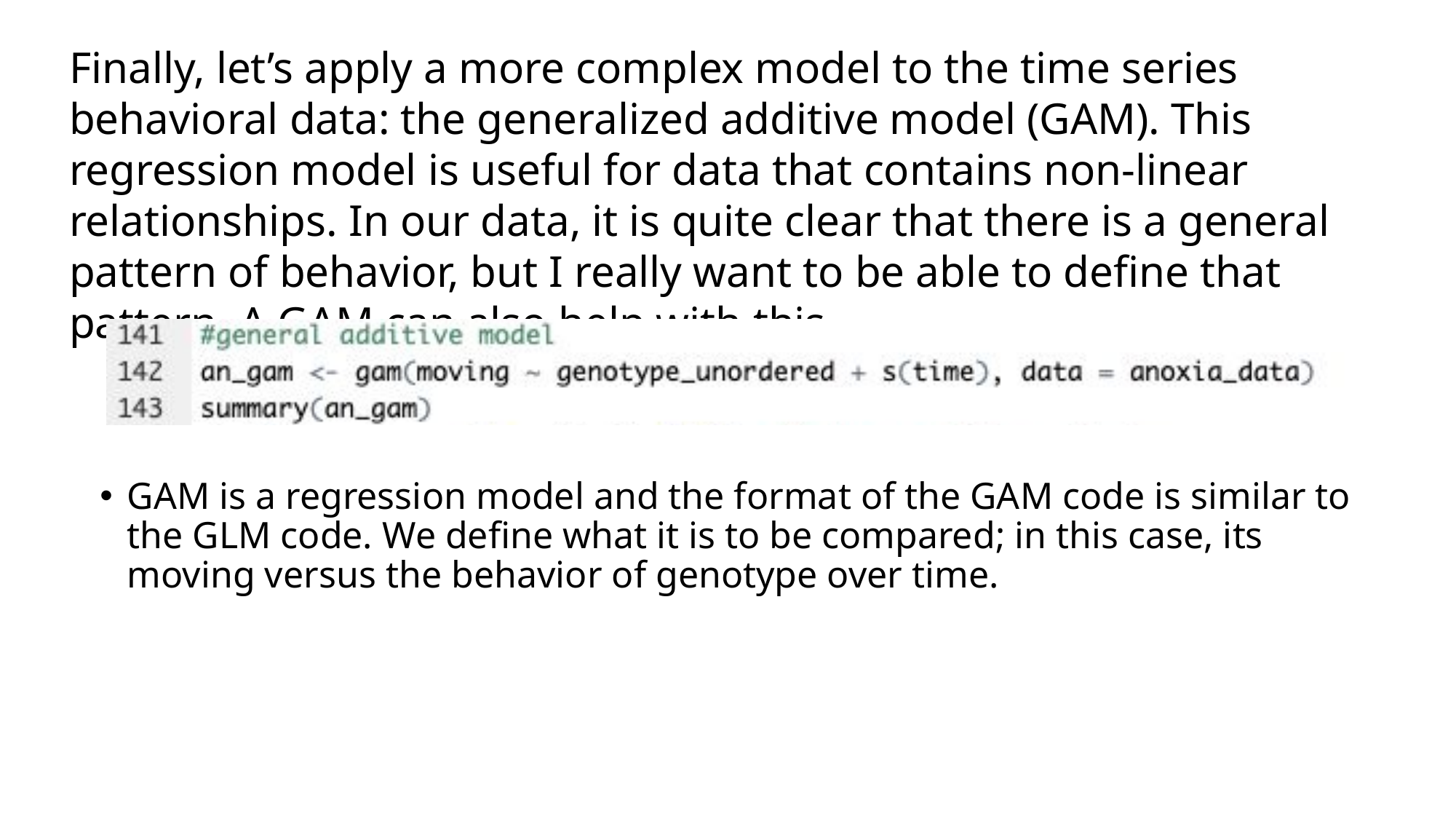

Finally, let’s apply a more complex model to the time series behavioral data: the generalized additive model (GAM). This regression model is useful for data that contains non-linear relationships. In our data, it is quite clear that there is a general pattern of behavior, but I really want to be able to define that pattern. A GAM can also help with this.
GAM is a regression model and the format of the GAM code is similar to the GLM code. We define what it is to be compared; in this case, its moving versus the behavior of genotype over time.

## Slide 37
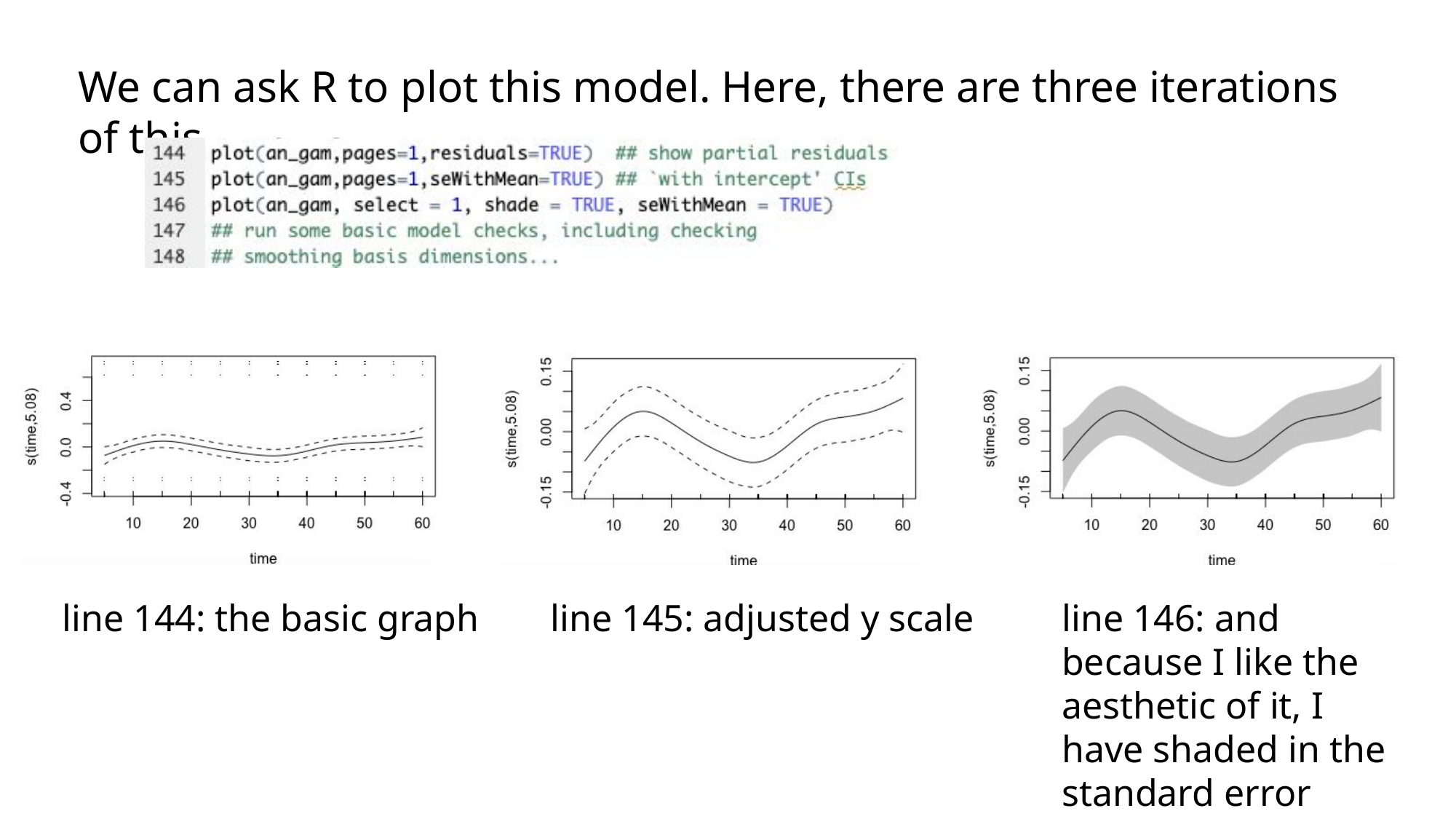

We can ask R to plot this model. Here, there are three iterations of this.
line 145: adjusted y scale
line 146: and because I like the aesthetic of it, I have shaded in the standard error
line 144: the basic graph

## Slide 38
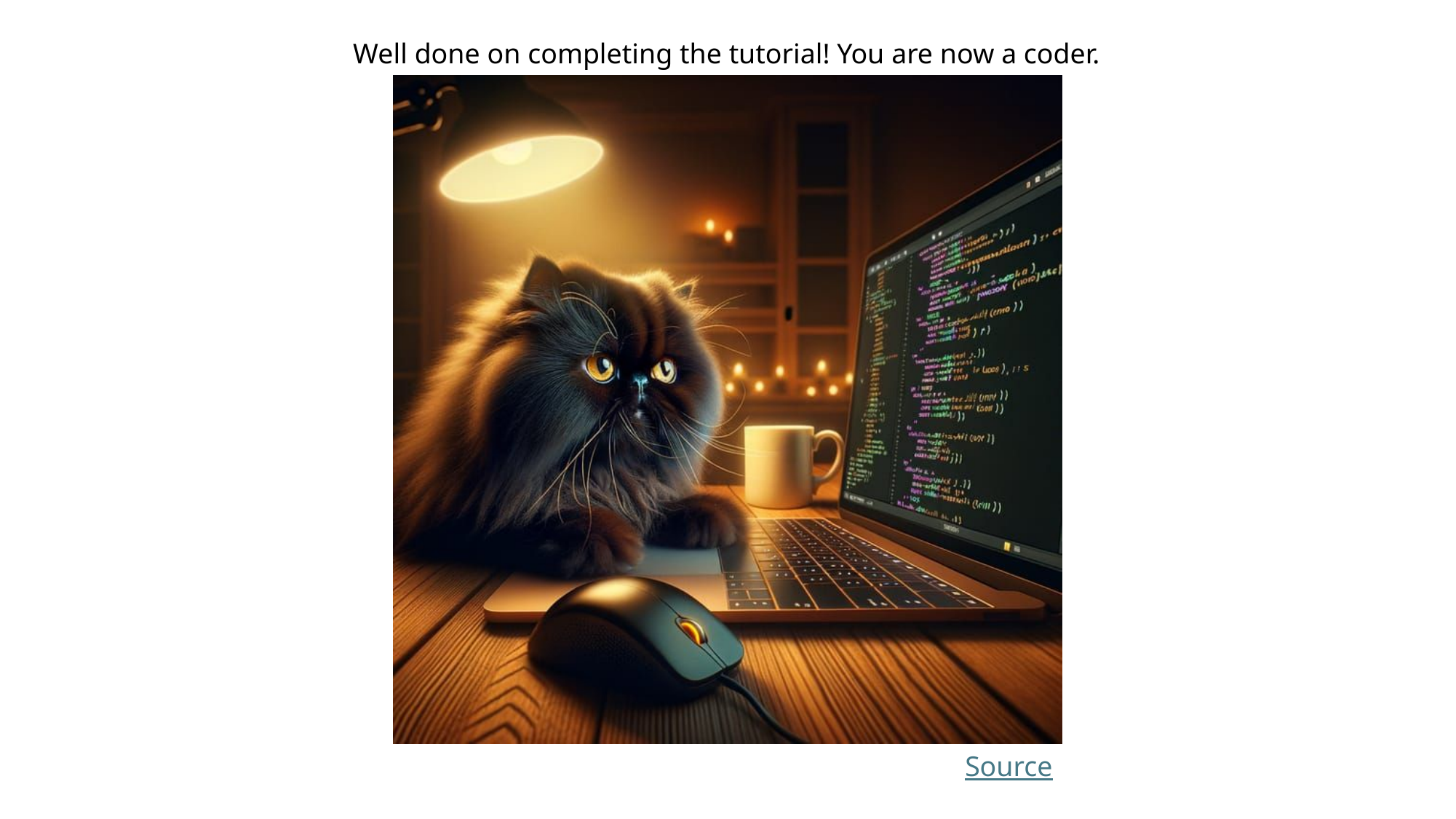

Well done on completing the tutorial! You are now a coder.
Source

## Slide 39
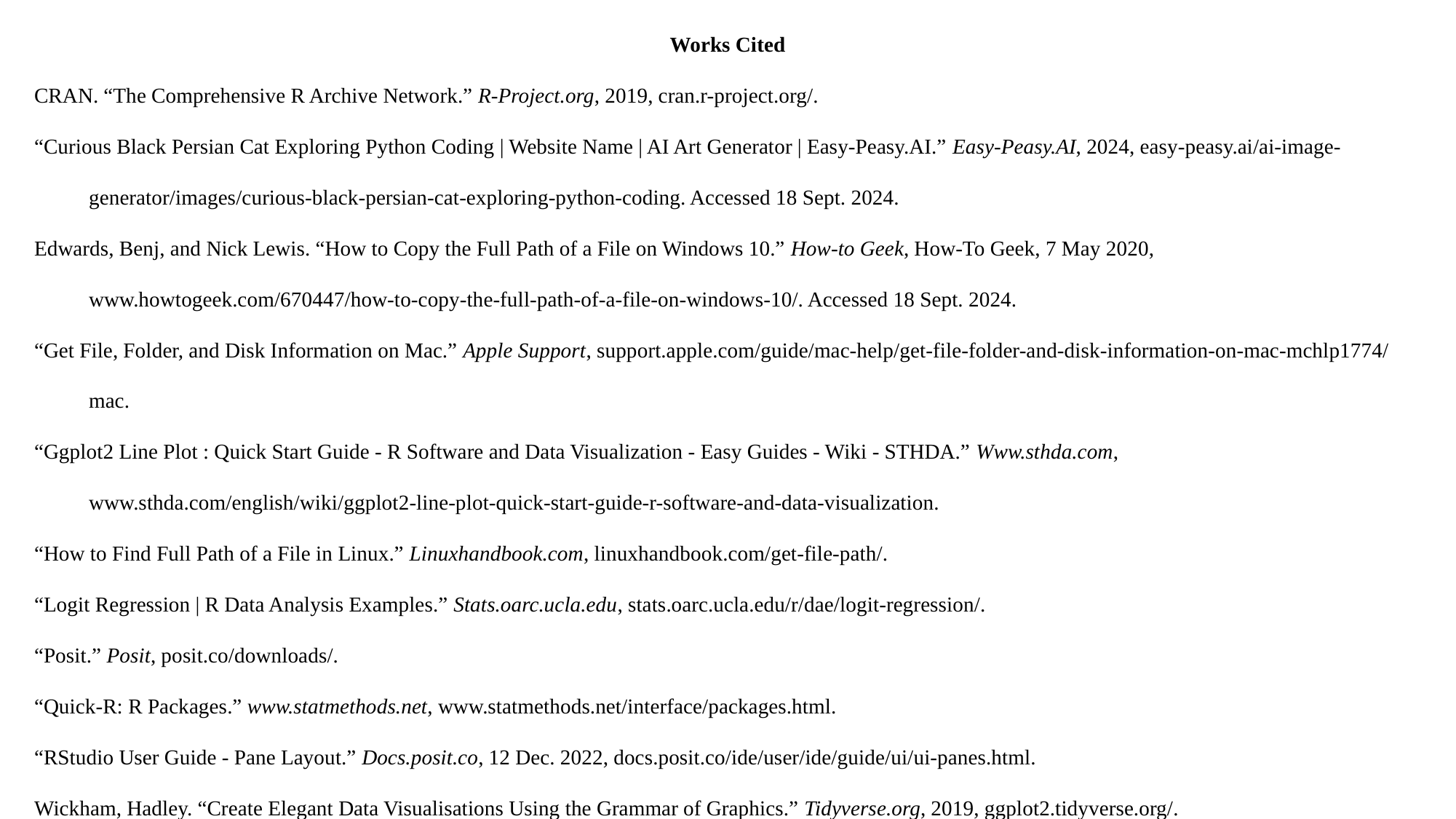

Works Cited
CRAN. “The Comprehensive R Archive Network.” R-Project.org, 2019, cran.r-project.org/.
“Curious Black Persian Cat Exploring Python Coding | Website Name | AI Art Generator | Easy-Peasy.AI.” Easy-Peasy.AI, 2024, easy-peasy.ai/ai-image-generator/images/curious-black-persian-cat-exploring-python-coding. Accessed 18 Sept. 2024.
Edwards, Benj, and Nick Lewis. “How to Copy the Full Path of a File on Windows 10.” How-to Geek, How-To Geek, 7 May 2020, www.howtogeek.com/670447/how-to-copy-the-full-path-of-a-file-on-windows-10/. Accessed 18 Sept. 2024.
“Get File, Folder, and Disk Information on Mac.” Apple Support, support.apple.com/guide/mac-help/get-file-folder-and-disk-information-on-mac-mchlp1774/mac.
“Ggplot2 Line Plot : Quick Start Guide - R Software and Data Visualization - Easy Guides - Wiki - STHDA.” Www.sthda.com, www.sthda.com/english/wiki/ggplot2-line-plot-quick-start-guide-r-software-and-data-visualization.
“How to Find Full Path of a File in Linux.” Linuxhandbook.com, linuxhandbook.com/get-file-path/.
“Logit Regression | R Data Analysis Examples.” Stats.oarc.ucla.edu, stats.oarc.ucla.edu/r/dae/logit-regression/.
“Posit.” Posit, posit.co/downloads/.
“Quick-R: R Packages.” www.statmethods.net, www.statmethods.net/interface/packages.html.
“RStudio User Guide - Pane Layout.” Docs.posit.co, 12 Dec. 2022, docs.posit.co/ide/user/ide/guide/ui/ui-panes.html.
Wickham, Hadley. “Create Elegant Data Visualisations Using the Grammar of Graphics.” Tidyverse.org, 2019, ggplot2.tidyverse.org/.
