# Supplementary material for: Analysis of categorical data from biological experiments with logistic regression and CMH tests
Source: PLoS One. 2025 Nov 17;20(11):e0335143. doi: 10.1371/journal.pone.0335143 (PMC12622779; doi:10.1371/journal.pone.0335143)
Supplement: S2 File — (ZIP) [file pone.0335143.s003.zip › Logistic-Regression-for-Biologists-main/Logistic_Regression_Fertility/Formatting_Input_Fertility_Code_Annotated.pdf]

Highlighted text are parts of this code that may need to be optimized to match your dataset

```
# Use this code to reformat your raw data from total instances into an excel
# spreadsheet listing each observation as a separate row.

library(readxl) #read excel spreadsheets into R
library(writexl) #write R data frames to excel
library(tidyr)  # Load the tidyr package

df <- read_excel("input/Male_Supplement_AD5.xlsx")
```

Change this to match the name of your raw dataset in the input folder

```
# Function to reshape the data
reshape_data <- function(df) {
  # Create a list to store individual worm observations
  data <- list()

  # Loop through each observation in the dataset
  for (i in 1:nrow(df)) {
    row <- df[i, ]

    # Extract original data except the title of the column, in this case "Exopher" and
"No_Exopher"
    original_data <- row[!(names(row) %in% c("Exopher", "No_Exopher"))]

    # Add rows for instances where the data is positive for "Exopher" with original data
    if (row$Exopher > 0) {
      for (j in 1:row$Exopher) {
        data[[length(data) + 1]] <- c(original_data, Exopher = 1, No_Exopher = 0)
      }
    }

    # Add rows for "No_Exophers" events with original data
    if (row$No_Exopher > 0) {
      for (j in 1:row$No_Exopher) {
        data[[length(data) + 1]] <- c(original_data, Exopher = 0, No_Exopher = 1)
      }
    }
  }

  # Combine worm observations into a data frame
  do.call(rbind, data)
}
```

Change these variables to match the column headers in your dataset

```
# Reshape the data frame- this will require tidyr later to format it as a table
df_expanded <- reshape_data(df)
# Print the expanded data frame
print(df_expanded)

data_expanded <- as.data.frame(df_expanded)
str(data_expanded) #checks the structure of the data- if the  result is a "list" then you
need to use tidyr to clean it up

#use tidyr to make the lists individual columns, this is formatting the data back into a
table
data_trial_unnested <- unnest(data_expanded, Trial)
data_treatment_unnested <- unnest(data_trial_unnested, Treatment)
data_exopher_unnested <- unnest(data_treatment_unnested, Exopher)
data_final <- unnest(data_exopher_unnested, No_Exopher)
```

These variables reference the column titles in your dataset

```
print(data_final) #check your work
write_xlsx(data_final, "input/fertility_exopher_final.xlsx") #write to an excel file
```

Indicate where you want to save the reformatted data and what you want to name the final spreadsheet
