# Supplementary material for: Analysis of categorical data from biological experiments with logistic regression and CMH tests
Source: PLoS One. 2025 Nov 17;20(11):e0335143. doi: 10.1371/journal.pone.0335143 (PMC12622779; doi:10.1371/journal.pone.0335143)
Supplement: S2 File — (ZIP) [file pone.0335143.s003.zip › Logistic-Regression-for-Biologists-main/Logistic_Regression_Fertility/Logistic Regression Fertility Annotated Output.pdf]

```
> ####section 3:statistical analysis####
>
>
> ##Reformatting the data for logistic regression
> # Define Treatment as a factor and make Umated your reference dataset
> Treatment_unordered <- factor(data$Treatment, ordered = FALSE) #makes Treat
ment a factor
> Treatment_unordered <- relevel(Treatment_unordered, ref = "Unmated") #makes
Unmated within Treatment the reference condition
>
> ##simple logistic regression
> data_glm <- glm(Exopher ~ Treatment_unordered+factor(Trial), family = binom
ial(link = "logit"), # the default is logit
+                 data = data)
> summary(data_glm)

Call:
glm(formula = Exopher ~ Treatment_unordered + factor(Trial),
    family = binomial(link = "logit"), data = data)

Coefficients:
                                Estimate Std. Error z value Pr(>|z|)
(Intercept)                      -3.8140     0.6375  -5.983 2.19e-09 ***
Treatment_unorderedFertile Male   2.6031     0.6283   4.143 3.43e-05 ***
Treatment_unorderedSterile Male   0.2989     0.7783   0.384    0.701
factor(Trial)2                    0.5940     0.3911   1.519    0.129
---
Signif. codes:  0 ‘***’ 0.001 ‘**’ 0.01 ‘*’ 0.05 ‘.’ 0.1 ‘ ’ 1

(Dispersion parameter for binomial family taken to be 1)

    Null deviance: 220.15  on 299  degrees of freedom
Residual deviance: 178.61  on 296  degrees of freedom
AIC: 186.61

Number of Fisher Scoring iterations: 6

> plot(data_glm)
Hit <Return> to see next plot:
Hit <Return> to see next plot:
Hit <Return> to see next plot:
Hit <Return> to see next plot:
> # Calculate odds ratios
> odds_ratios <- exp(coef(data_glm))
> print(odds_ratios)

(Intercept)                          0.02205892
Treatment_unorderedFertile Male     13.50538752
Treatment_unorderedSterile Male      1.34835394
factor(Trial)2                       1.81115063

>
> # Calculate 95% confidence intervals for the odds ratios
> conf_intervals <- exp(confint(data_glm))
Waiting for profiling to be done...
> print(conf_intervals)
                                      2.5 %      97.5 %
(Intercept)                     0.005021454  0.06572311
Treatment_unorderedFertile Male 4.548799663 58.13122488
Treatment_unorderedSterile Male 0.289360748  7.00801585
factor(Trial)2                  0.849779032  3.97578891
```

These are the results for the comparison between unmated (control treatment) and mating with a fertile male.

This is the comparison between the unmated control and mating with a sterile male.

These are the p-values for each comparison. Mating with the fertile male results in significantly different rates of exopher production. While mating with the sterile male does not change exopher rates from the unmated control.

The z-value indicates that mating with the fertile male increases exopher rates by more than four standard deviations above the mean of the control.

Odds Ratio for the logistic regression. The animals that are mated with fertile males are 13 times more likely to have an exopher.

95% confidence interval of the odds ratio. Note that the confidence interval range for animals that were mated with a sterile male is 0.2-7 and includes 1, thus it's likely that there is no difference between the control and this treatment.
