# Supplementary material for: Analysis of categorical data from biological experiments with logistic regression and CMH tests
Source: PLoS One. 2025 Nov 17;20(11):e0335143. doi: 10.1371/journal.pone.0335143 (PMC12622779; doi:10.1371/journal.pone.0335143)
Supplement: S2 File — (ZIP) [file pone.0335143.s003.zip › Logistic-Regression-for-Biologists-main/Logistic_Regression_Fertility/Logistic_Regression_Fertility_Code_Annotated.pdf]

Highlighted text are parts of this code that may need to be optimized to match your dataset

```
################################################
# Regression models on exopher frequency data #
# January 21 2025                              #
################################################

#text like this with a hashtag before it is called a comment and is not executable code.
#to execute a line of code on a mac, go to the end of the line and hit command+shift+enter
####<---you can use the arrows next to the lines numbers to collapse or fold in a section####



####section 1:setup####

#First! install the following packages using install.packages("name of package") in the CONSOLE of RStudio:
#ggsignif
#dplyr
#readxl
#writexl
#ggplot2
#ggthemes
#ggthemeAssist
#stats
#ggsignif
#ggpubr
#forcats
#ggprism

#next, load the libraries by executing code in the upper part of the screen where all this code is. Again, to execute a line of code on a mac, go to the end of the line and hit command+shift+enter
library(dplyr) #datamanipulation
library(readxl) #read excel spreadsheets into R
library(writexl) #convert R data frames into excel spreadsheet
library(ggplot2) #visualizations besides basics plots
library(ggthemes) #adds more themes to the ggplot2 package
library(ggThemeAssist) #provides a graphical user interface (GUI) for customizing ggplot2 themes
library(stats) #basic statistical package
library(ggsignif) #To add significance stars to your ggplot2 graph
library(forcats) #To reorder the data
library(ggprism) #To generate the plots in a minimalist theme

#import your excel spreadsheet; n.b. this will only read the first sheet in the workbook
data <- read_excel("input/fertility_exopher_final.xlsx")
```

Change this to match the name of your raw dataset in the input folder

```
####section 2:see what your data looks like####

##generate plot of your dataframe
#summarizing data as percentages to plot
data_percent <- data %>%
  group_by(Treatment, Trial) %>%
  summarize(
    data_percent = (100 * ((sum(Exopher, na.rm = TRUE)) / n()))
  )
```

Change these variables to match the column headers in your dataset
**Capitalization matters

```
data_summary <- data %>%
  group_by(Treatment, Trial) %>%
  summarize(
    data_percent = (100 * ((sum(Exopher, na.rm = TRUE)) / n())),
    # Calculate standard error of the percentage (SEP) using the formula sqrt((p * (1 - p)) / n)
    data_sep = sqrt((data_percent / 100) * (1 - (data_percent / 100)) / n()) * 100
```

```
  ) %>%
  group_by(Treatment) %>%
  summarize(
    data_percent_mean = mean(data_percent),
    data_sep_mean = mean(data_sep)  # Average SEP across trials
  )

#plot exopher percentages and SEP

# Reorder the Treatment factor so that "unmated" is first
data_summary$Treatment <- fct_relevel(data_summary$Treatment, "Unmated", "Sterile Male",
"Fertile Male")

# Check the levels of the Treatment factor
levels(data_summary$Treatment)

Plotted_data <- ggplot(data_summary, aes(x = Treatment, y = data_percent_mean, fill =
Treatment)) +
  # Add bars with black borders
  geom_bar(stat = "identity", width = 0.7, color = "black", size = 1.5) +

  # Overlay a scatterplot to show individual trial averages
  geom_point(data = data_percent,  # Use the trial_means data
             aes(x = Treatment, y = data_percent, group = Trial),  # Map trial means
             position = position_dodge(width = 0.5),
             alpha = 0.5,
             size= 2.5)  +

  # Step 2: Add error bars using the summarized data (using ymin and ymax correctly)
  geom_errorbar(aes(ymin = data_percent_mean - data_sep_mean, ymax = data_percent_mean +
data_sep_mean),
                width = 0.2, linetype = "solid", linewidth = 1.5) +

  # Step 3: Add significance annotations with a thicker bracket line
  geom_signif(comparisons = list(c("Unmated", "Fertile Male")),
              annotations = c("***"),
              y_position = 36.5,  # Increased y_position to make space
              textsize = 8,
              tip_length = 0.05,
              vjust = 0.5,
              size = 1.5,  # Increase bracket line thickness
              linetype = "solid", color = "black") +  # Solid line and black color

  geom_signif(comparisons = list(c("Unmated", "Sterile Male")),
              annotations = c("ns"),
              y_position = 8,  # Adjusted y_position for visibility
              textsize = 8,
              tip_length = 0.05,
              vjust = -0.5,  # Move the "ns" text above the bracket
              size = 1.5,  # Increase bracket line thickness
              linetype = "solid", color = "black") +  # Solid line and black color
```

Indicate the variables for comparison within the Treatment group

```
  # Step 4: Add labels and titles
  labs(title = "Exopher Frequency",  # Title updated
       x = "Fertility Status",  # X-axis title updated
       y = "Percent Exophers") +  # Y-axis title updated
```

Specify the titles for your graph and the axes

```
  # Step 5: Set y-axis limits and ensure there's space for the significance
  coord_cartesian(ylim = c(0, 40)) +  # Adjust y-limit for enough space for annotations

  # Adjust the x-axis horizontal line to intersect with 0 (using expand)
  scale_y_continuous(expand = c(0, 0)) +  # Removes the padding around the plot

  # Step 6: Use minimal theme and increase text sizes
  theme_prism() +
```

```
  theme(
    legend.position = "none",  # Remove the legend
    axis.text.x = element_text(size = 20, angle = 45, hjust = 1, vjust = 1,face = "bold",
color = "black"),  # x-axis labels left-aligned
    axis.text.y = element_text(size = 20, face = "bold", color = "black"),
    axis.title.x = element_text(size = 22, face = "bold", color = "black", hjust = 0.5),
# Adjust hjust for x-axis title alignment
    axis.title.y = element_text(size = 22, face = "bold", color = "black"),
    plot.title = element_text(size = 24, face = "bold", color = "black", hjust = 0.5)
  )

# Print the plot
print(Plotted_data)

#save your plot for export as an SVG vector format
ggsave("exophermatingeffect.svg", plot = Plotted_data, width = 6, height = 6)

####section 3:statistical analysis####


##Reformatting the data for logistic regression
# Define Treatment as a factor and make Umated your reference dataset
Treatment_unordered <- factor(data$Treatment, ordered = FALSE) #makes Treatment a factor
Treatment_unordered <- relevel(Treatment_unordered, ref = "Unmated") #makes Unmated within
Treatment the reference condition

##simple logistic regression
data_glm <- glm(Exopher ~ Treatment_unordered+factor(Trial), family = binomial(link =
"logit"), # the default is logit
                   data = data)
summary(data_glm)
plot(data_glm)

# Calculate odds ratios
odds_ratios <- exp(coef(data_glm))
print(odds_ratios)

# Calculate 95% confidence intervals for the odds ratios
conf_intervals <- exp(confint(data_glm))
print(conf_intervals)
```
