# Supplementary material for: Analysis of categorical data from biological experiments with logistic regression and CMH tests
Source: PLoS One. 2025 Nov 17;20(11):e0335143. doi: 10.1371/journal.pone.0335143 (PMC12622779; doi:10.1371/journal.pone.0335143)
Supplement: S2 File — (ZIP) [file pone.0335143.s003.zip › Logistic-Regression-for-Biologists-main/anoxiacode_tutorial.pdf]

A pictorial tutorial for R

A companion guide to

[Paper Citation]

Part 1: Software Loading

Step 1:

- Download R for your operating system (OS)
- https://cran.r-project.org/

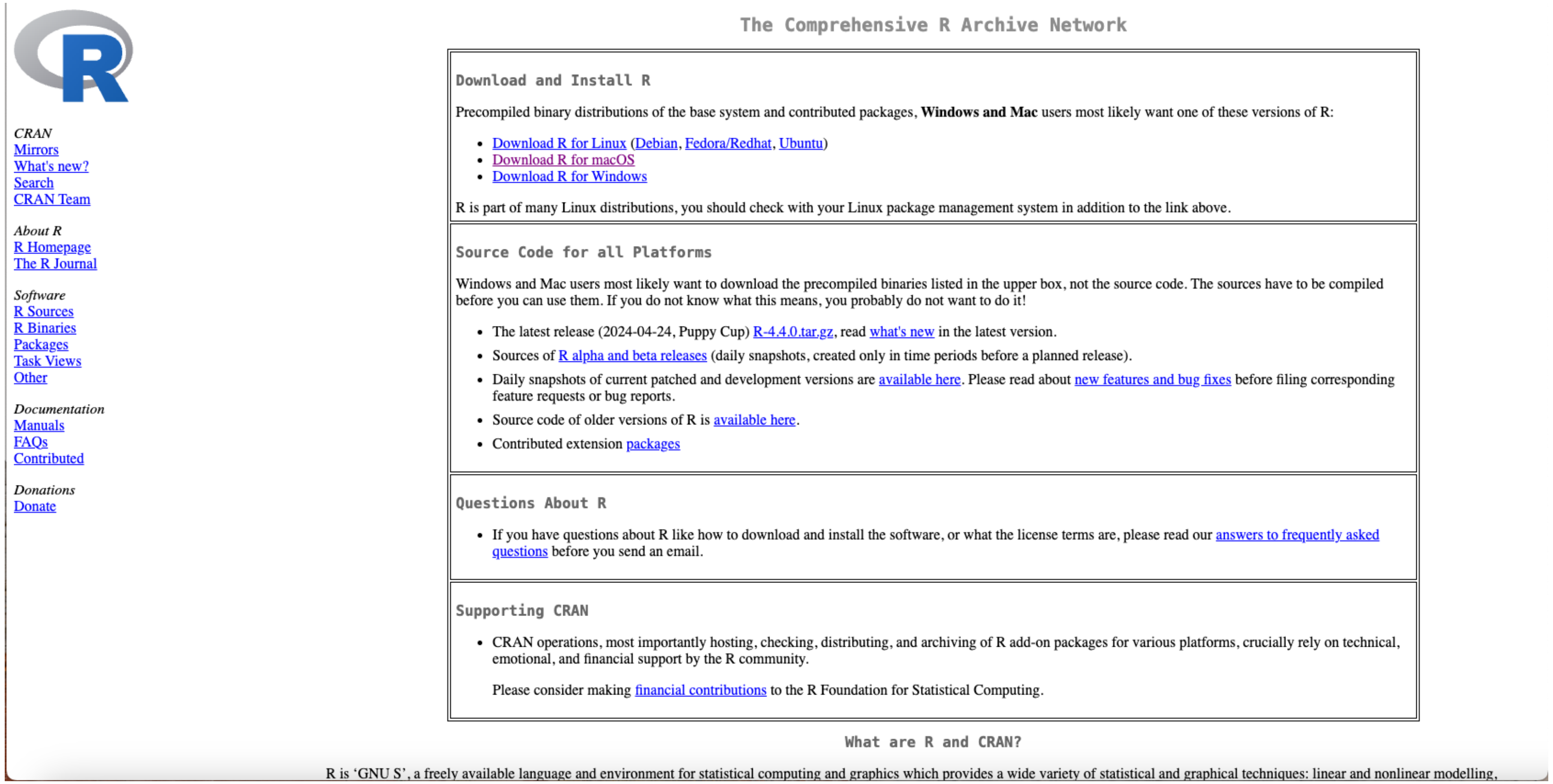

CRAN
Mirrors
What's new?
Search
CRAN Team

About R
R Homepage
The R Journal

Software
R Sources
R Binaries
Packages
Task Views
Other

Documentation
Manuals
FAQs
Contributed

Donations
Donate

The Comprehensive R Archive Network

Download and Install R

Precompiled binary distributions of the base system and contributed packages, **Windows and Mac** users most likely want one of these versions of R:

- Download R for Linux (Debian, Fedora/Redhat, Ubuntu)
- Download R for macOS
- Download R for Windows

R is part of many Linux distributions, you should check with your Linux package management system in addition to the link above.

Source Code for all Platforms

Windows and Mac users most likely want to download the precompiled binaries listed in the upper box, not the source code. The sources have to be compiled before you can use them. If you do not know what this means, you probably do not want to do it!

- The latest release (2024-04-24, Puppy Cup) R-4.4.0.tar.gz, read what's new in the latest version.
- Sources of R alpha and beta releases (daily snapshots, created only in time periods before a planned release).
- Daily snapshots of current patched and development versions are available here. Please read about new features and bug fixes before filing corresponding feature requests or bug reports.
- Source code of older versions of R is available here.
- Contributed extension packages

Questions About R

- If you have questions about R like how to download and install the software, or what the license terms are, please read our answers to frequently asked questions before you send an email.

Supporting CRAN

- CRAN operations, most importantly hosting, checking, distributing, and archiving of R add-on packages for various platforms, crucially rely on technical, emotional, and financial support by the R community.

  Please consider making financial contributions to the R Foundation for Statistical Computing.

What are R and CRAN?

R is 'GNU S', a freely available language and environment for statistical computing and graphics which provides a wide variety of statistical and graphical techniques: linear and nonlinear modelling,

Step 2:

- Download RStudio for your OS
- https://posit.co/downloads/

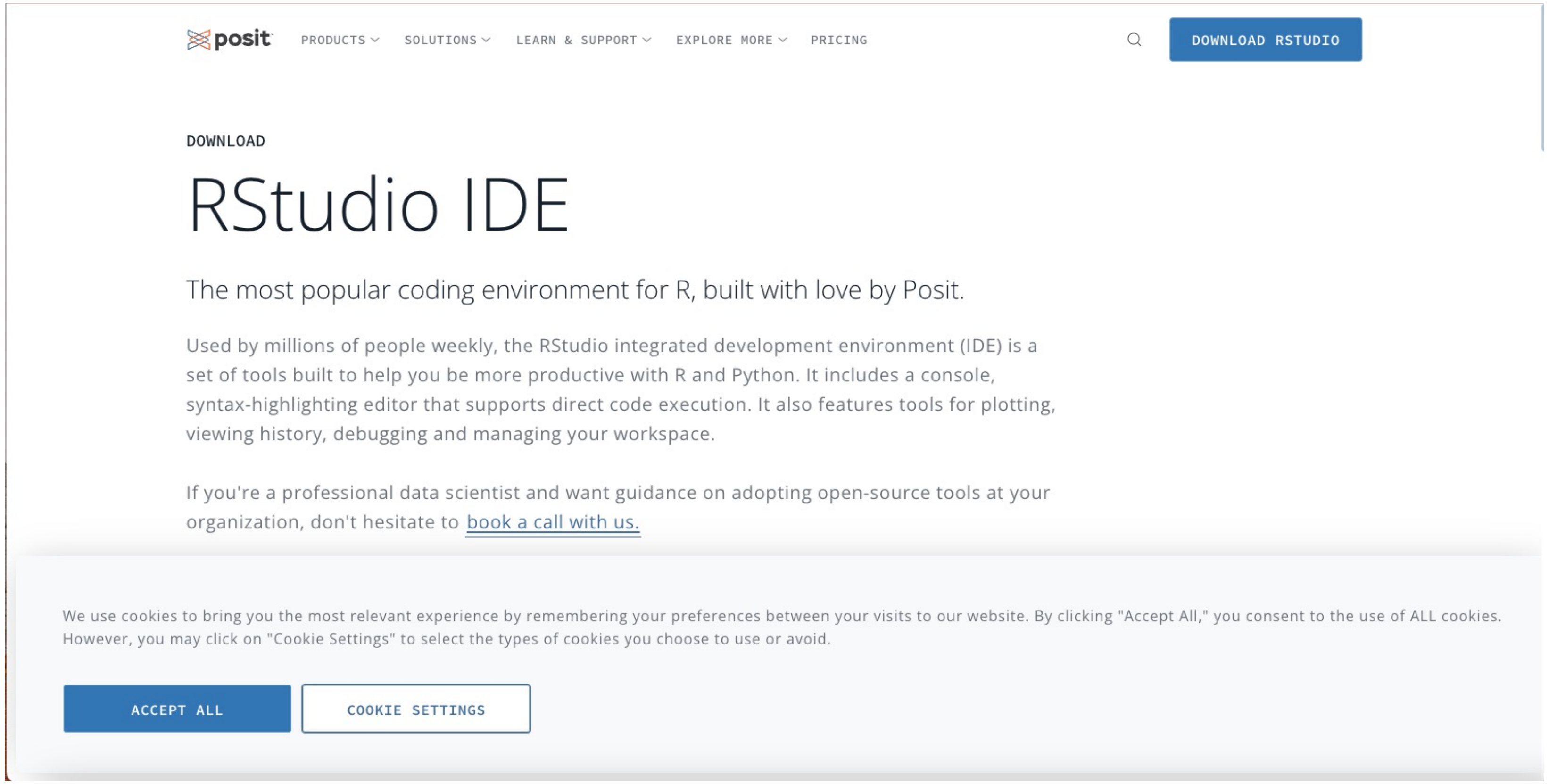

Part 2: Setting up your coding enviornment

Step 1:

- Open R Studio
- More information on the organization of the window: https://docs.posit.co/ide/user/ide/guide/ui/ui-panes.html

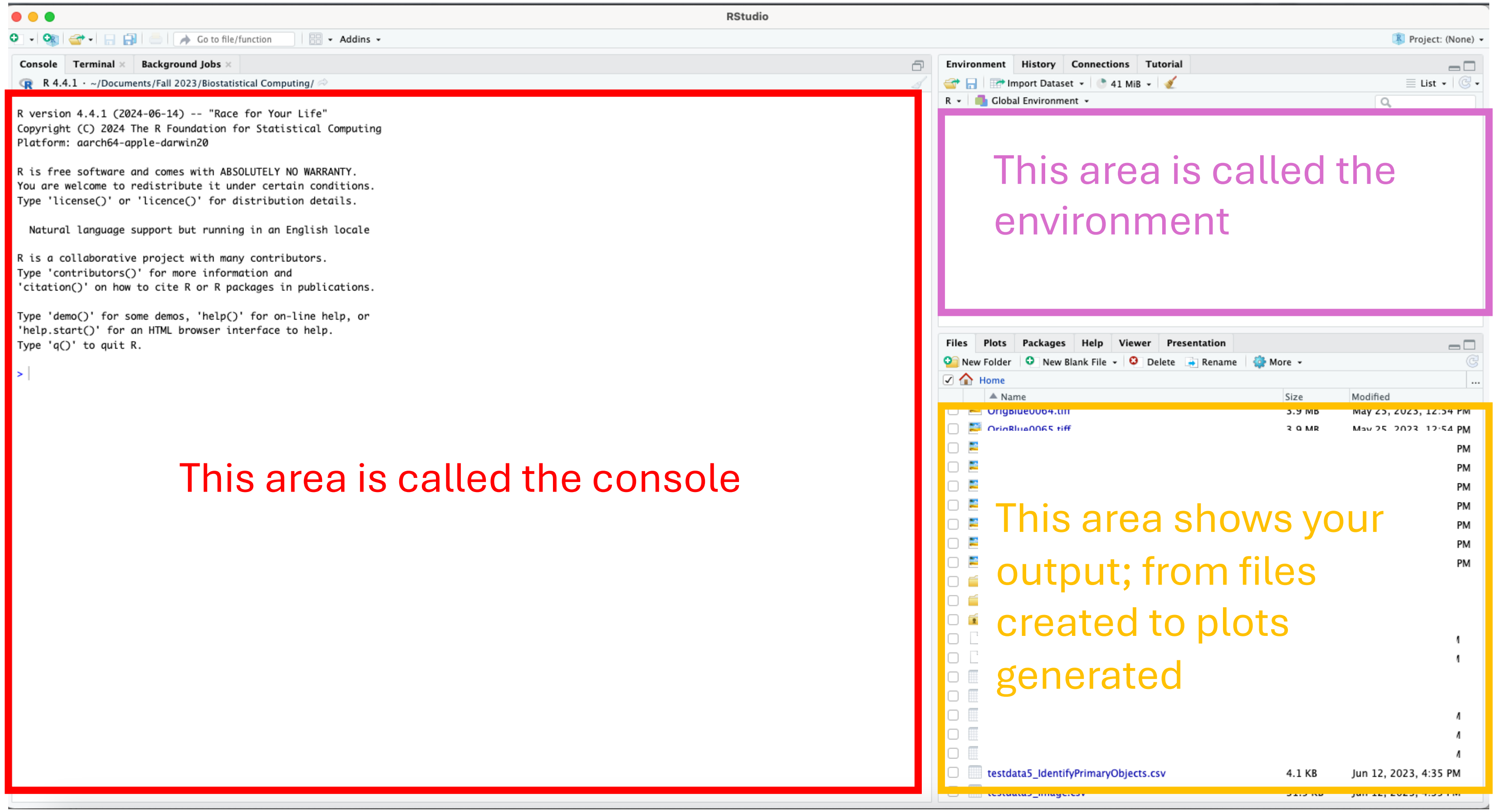

Step 2:

- Open the project file (highlighted in pink below)

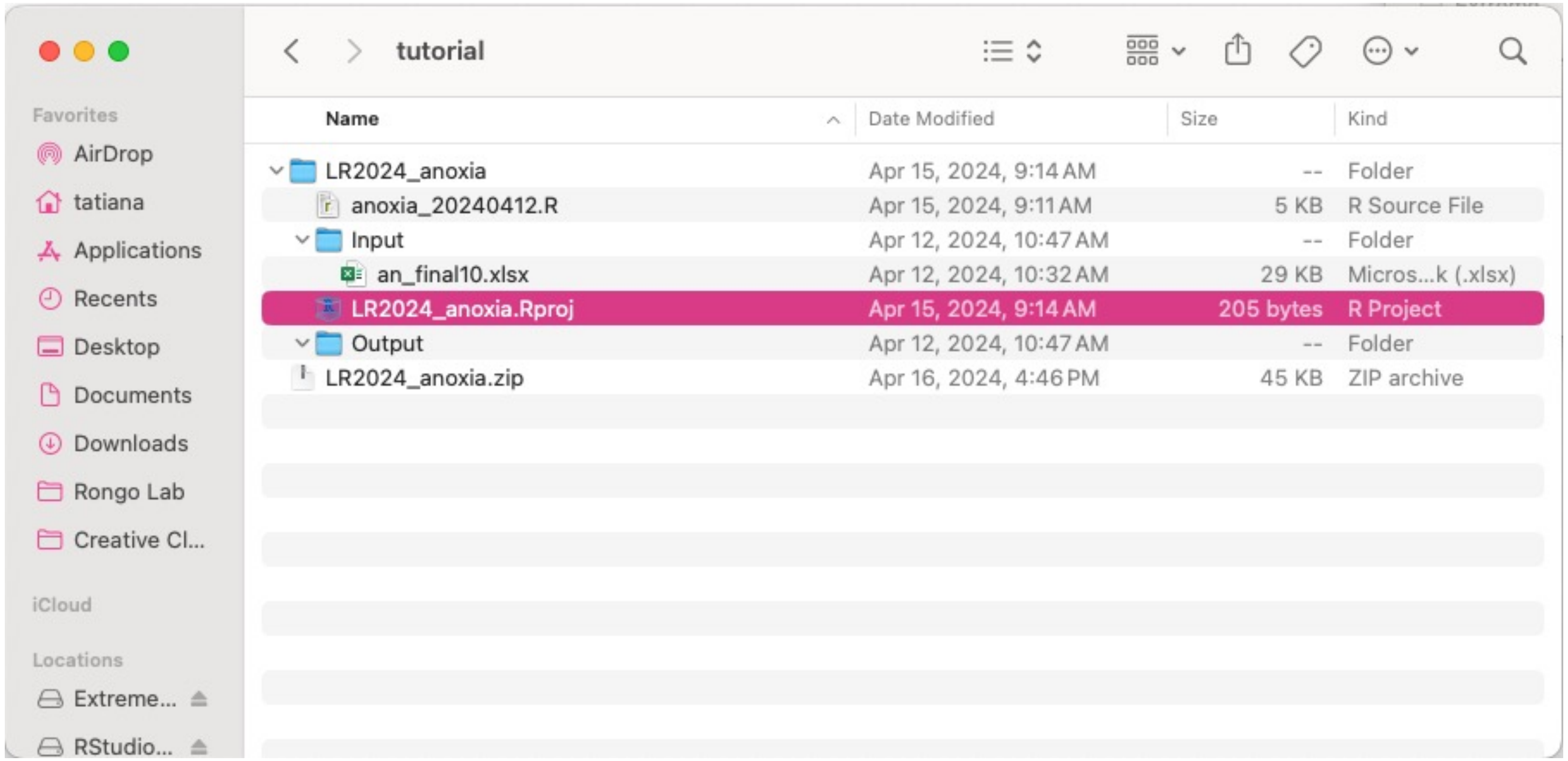

- Your R studio screen will now look like this- notice the addition of a new area!

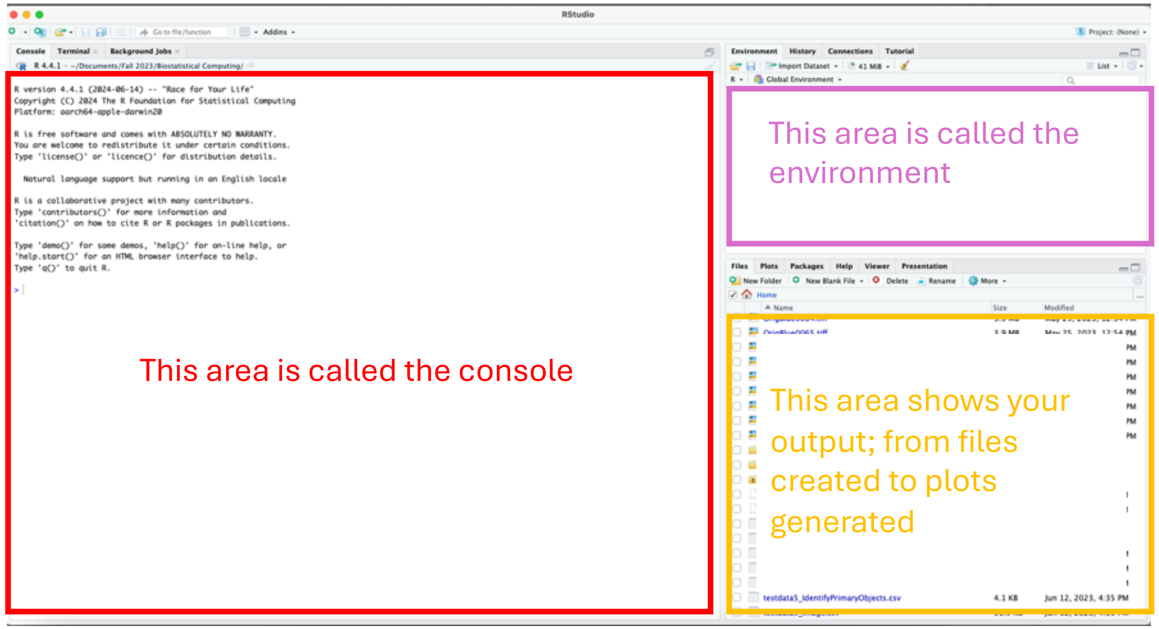

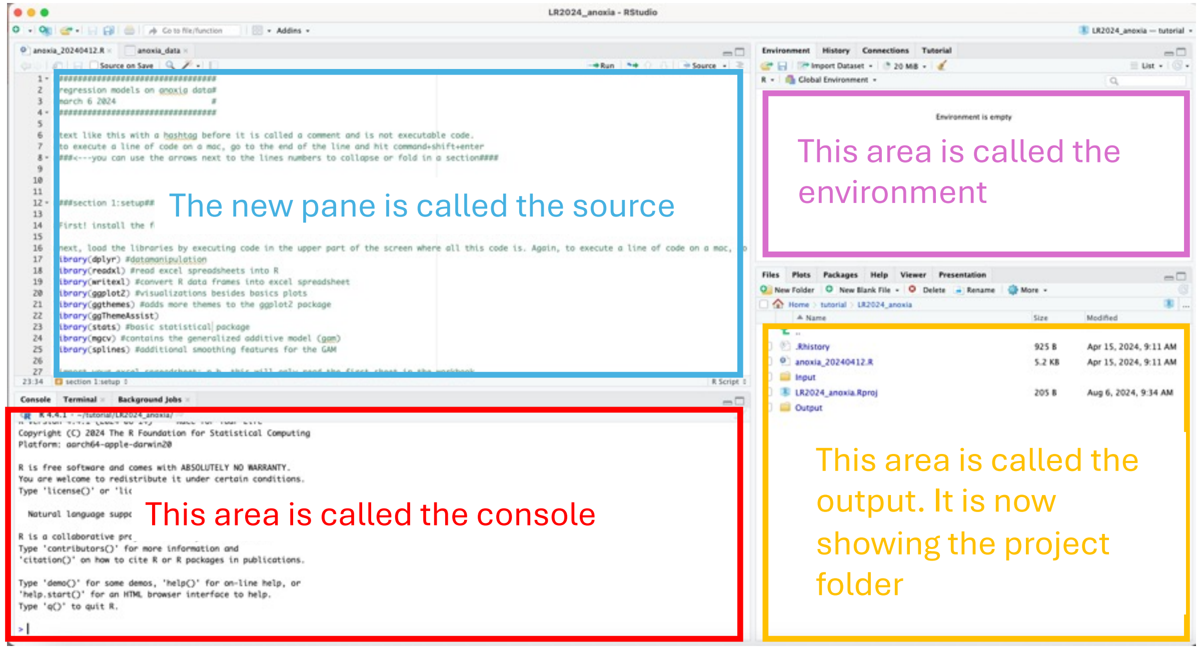

Part 3: Coding

Part 3: Coding

- You will notice that the source is already full of code if you opened the project file. You will be going through the code line by line to generate the regression models and the graphs in this project.

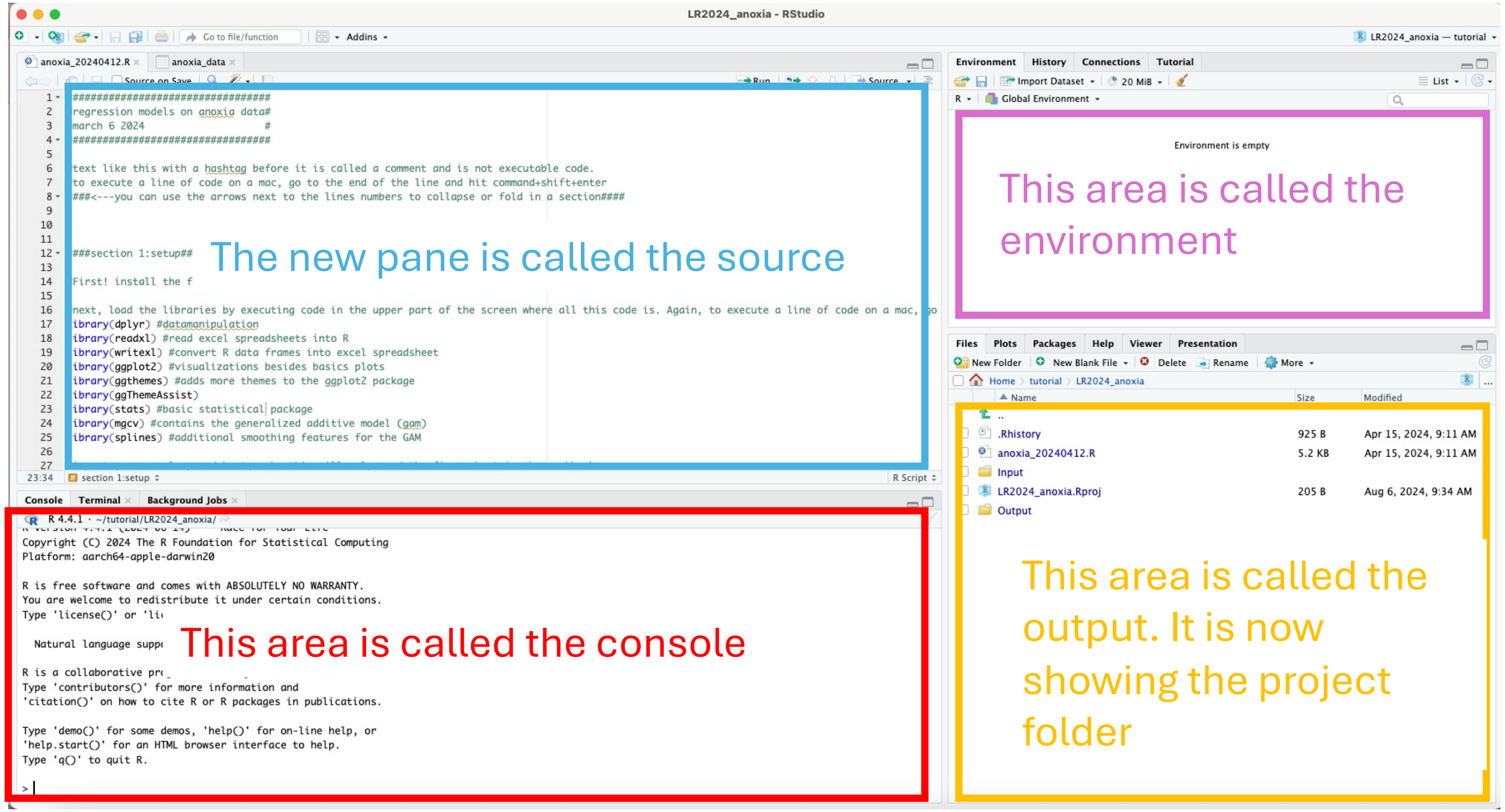

Part 3: Coding

- Each step will refer to line numbers, found here:

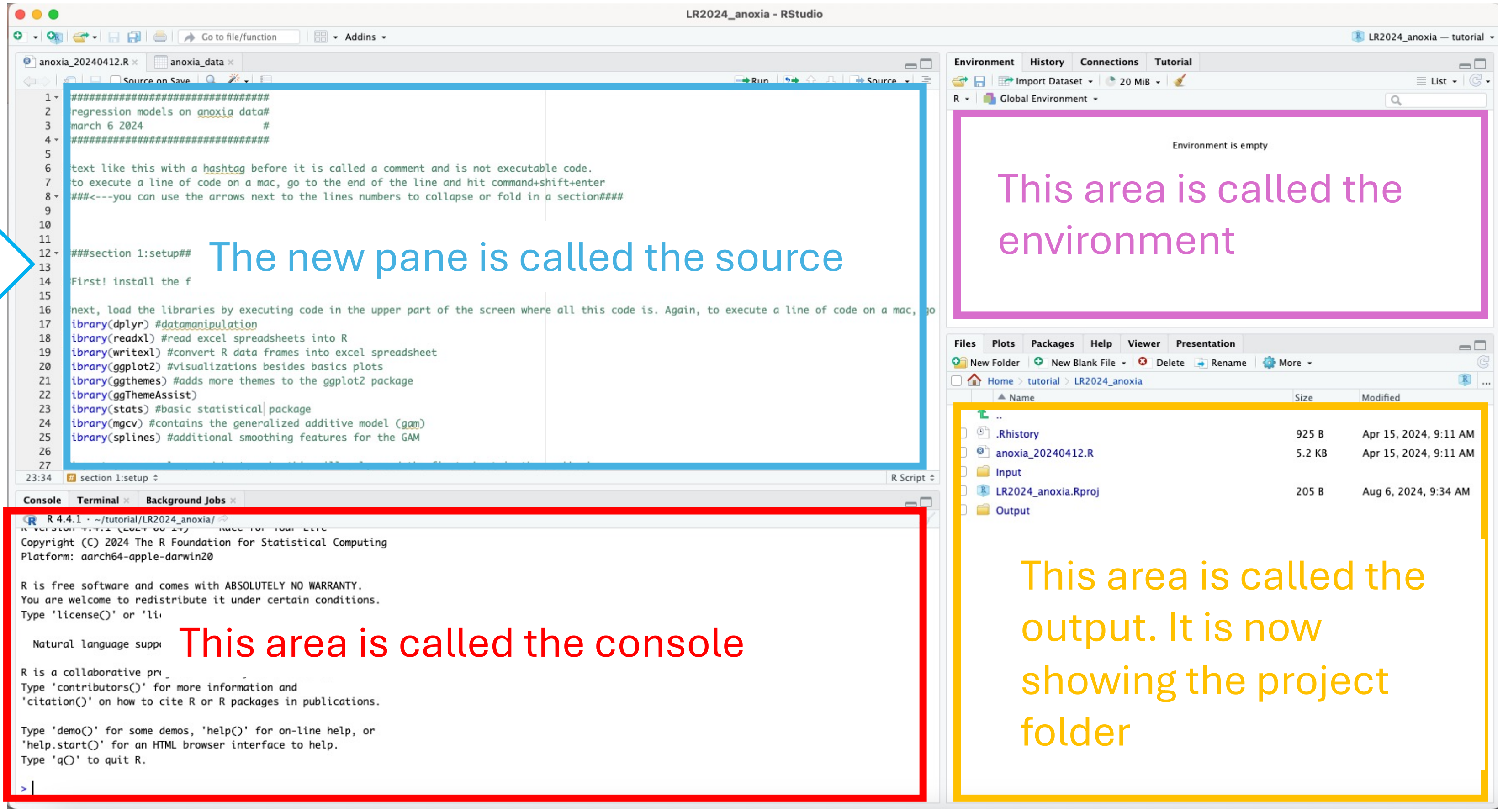

Notice the text colors that indicate the kind of code. In my screen, green is a note, blue is a command, black is an object.

Notes are also indicated by the hashtag # symbol. The first 17 lines are all notes, but line 15 contains the first action you will perform.

anoxia_20240412.R | anoxia_data

Source on Save | Run | Source

```
4   ####################################
5
6   #text like this with a hashtag before it is called a comment and is not executable code.
7   #to execute a line of code on a mac, go to the end of the line and hit command+shift+enter
8   ####<---you can use the arrows next to the lines numbers to collapse or fold in a section####
9
10
11
12  ####section 1:setup####
13
14  #First! install the following packages using install.packages("name of package") in the CONSOLE of RStudio
15  #use this code to install all packages at once:  install.packages(c('dplyr', 'readxl', 'writexl', 'ggplot2',
16
17  #next, load the libraries by executing code in the upper part of the screen where all this code is. Again, tc
18  library(dplyr) #datamanipulation
19  library(readxl) #read excel spreadsheets into R
20  library(writexl) #convert R data frames into excel spreadsheet
```

15:36 | section 1:setup | R Script

Part 3: Coding

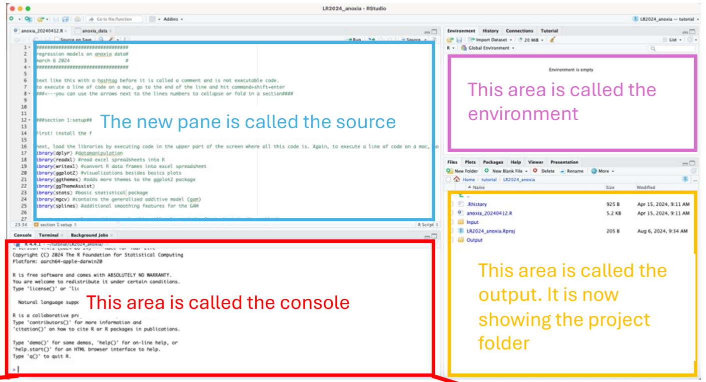

Section 1: Setup

- lines 14-15:
- Install the packages by pasting this line of code into the console, and hit enter:
- install.packages(c('dplyr', 'readxl', 'writexl', 'ggplot2', 'ggthemes', 'ggThemeAssist', 'stats', 'mgcv', 'splines’))
- *This step requires an active internet connection*

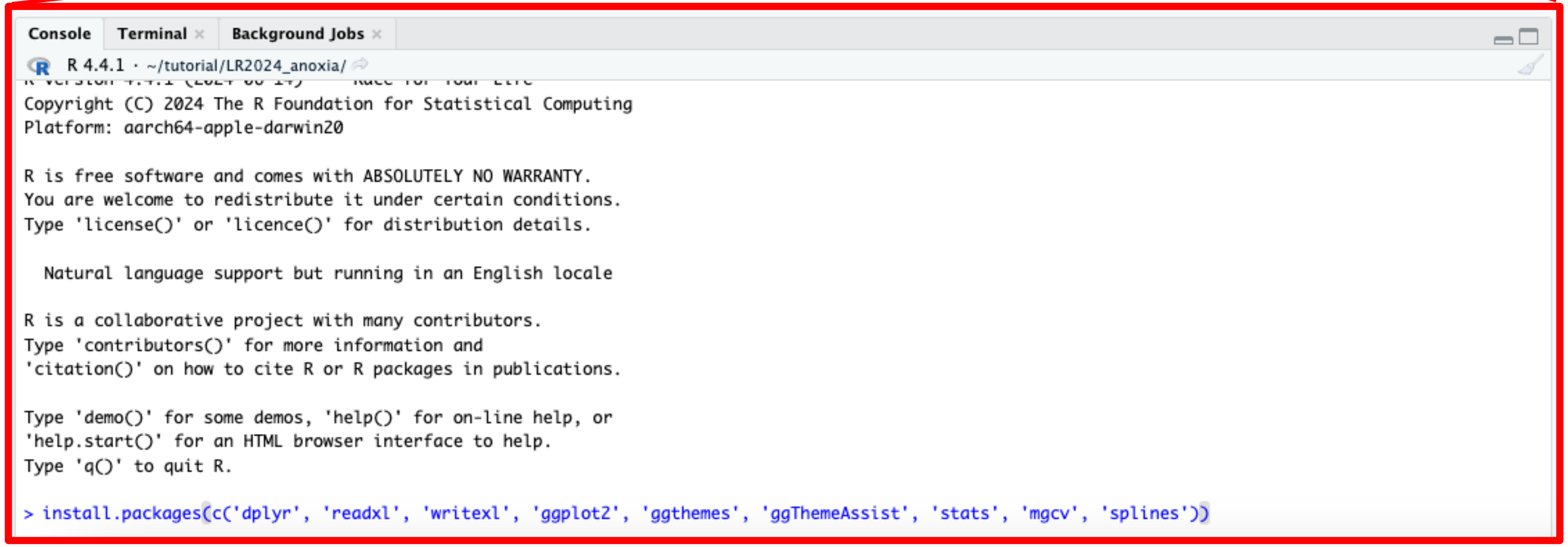

More information on packages: https://www.statmethods.net/interface/packages.html

- lines 17-26:
- After installing packages, you now have access to the libraries contained within those packages. Load the libraries by highlighting lines 17-26 in the source and hit command+enter on your keyboard to execute:

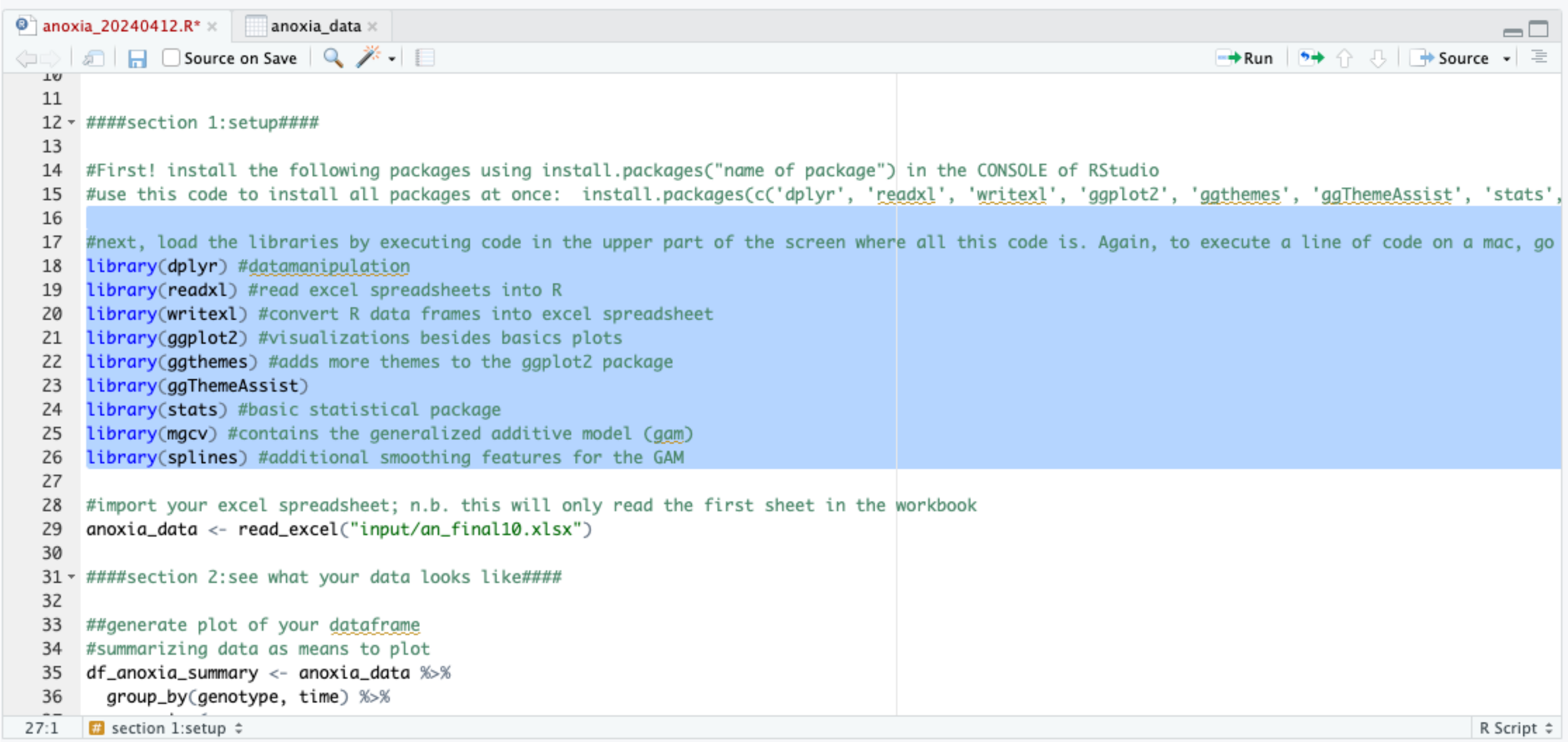

- lines 17-26:
- The command in the source that you just executed will be shown as a task having been done in the console:

```
Console   Terminal ×   Background Jobs ×
R 4.4.1 · ~/tutorial/LR2024_anoxia/
> library(dplyr) #datamanipulation

Attaching package: ‘dplyr’

The following objects are masked from ‘package:stats’:

    filter, lag

The following objects are masked from ‘package:base’:

    intersect, setdiff, setequal, union

> library(readxl) #read excel spreadsheets into R
> library(writexl) #convert R data frames into excel spreadsheet
> library(ggplot2) #visualizations besides basics plots
> library(ggthemes) #adds more themes to the ggplot2 package
> library(ggThemeAssist)
> library(stats) #basic statistical package
> library(mgcv) #contains the generalized additive model (gam)
```

- lines 28-29:
- Import your data. The project data is in the form of an excel spreadsheet (called “an_final10.xlsx”). If your data is in another kind of data table, you will search for the correct command to import that specific file format. This command (“read_excel”) imports excel spreadsheets into R.

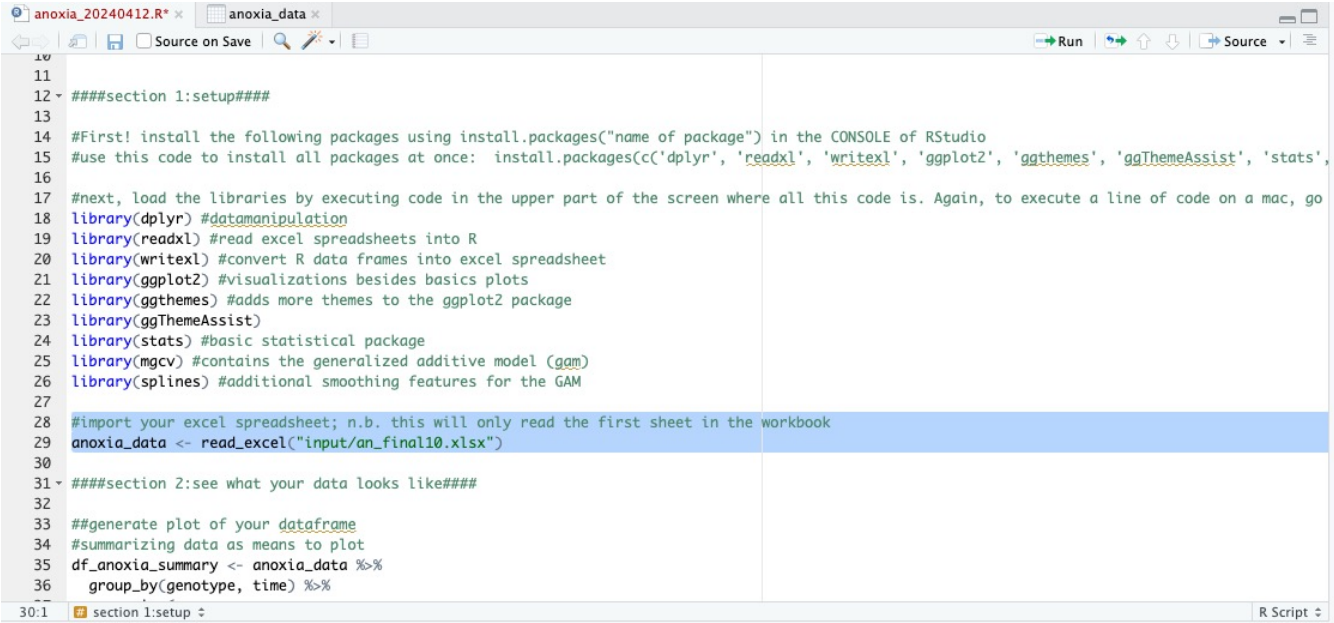

Notice that when you execute line 29, the new object you created (the R version of your excel spreadsheet) appears in your environment pane. If you click on that object in your environment pane, it will open in a new window in the R studio program next to your source code.

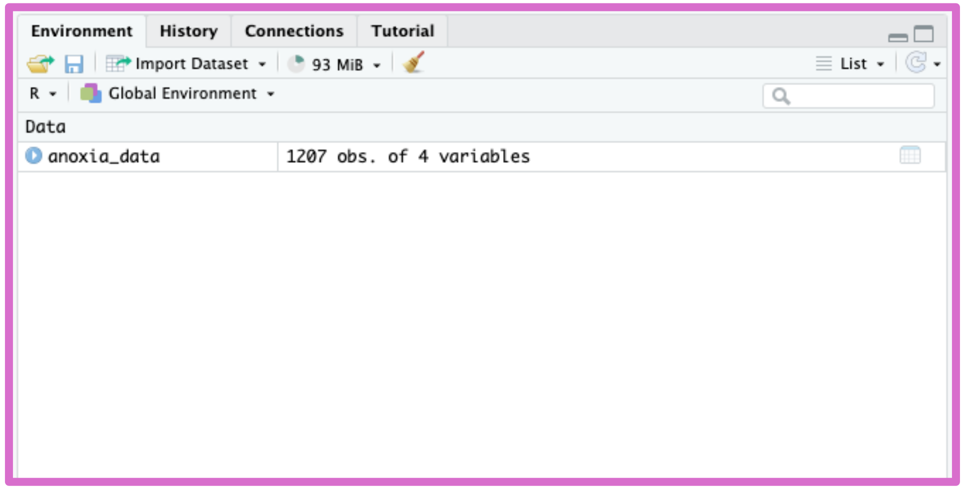

Let’s take a second to dissect the anatomy of a command in R.

```r
anoxia_data <- read_excel("input/an_final10.xlsx")
```

object

command

path where to find the file to read

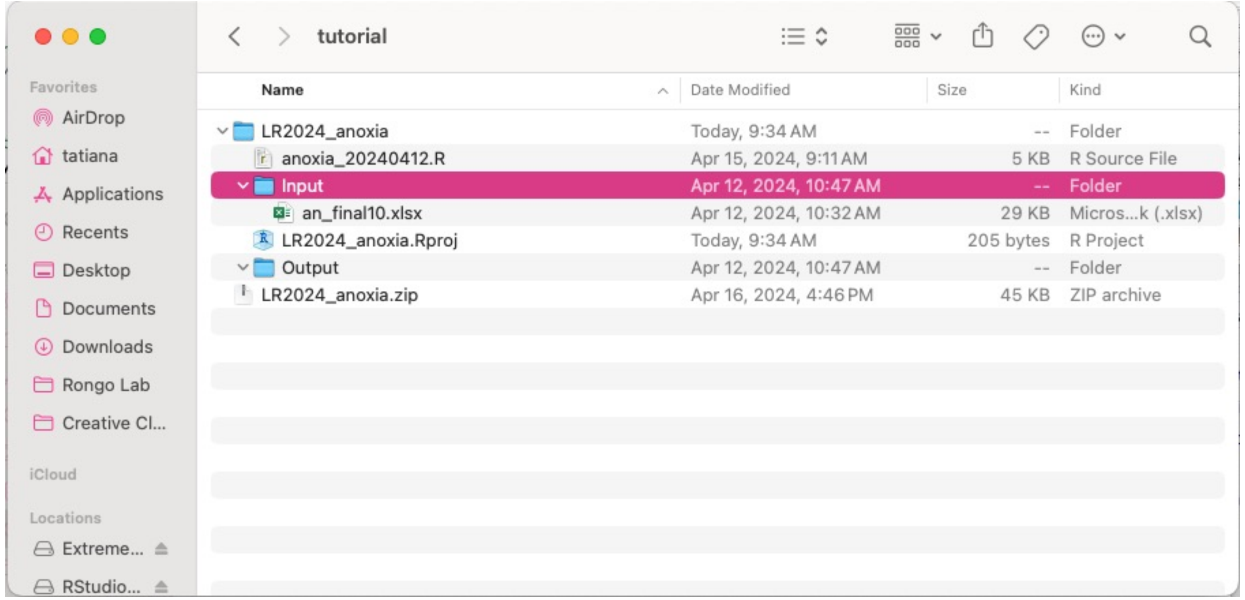

In this case, I am telling R to look in the folder called input for a file called an_final10.xlsx. You will notice that in the .zip file you downloaded (LR2024_anoxia.zip), there is the project file, the R code, and two folders called input and output. Opening up a project tells R you will work in the folder the project is located. If you wanted to import a file not in this folder, then just write out the full path of the file.

More information on figuring out a path: [Mac] [PC] [Linux]

We are now done with section 1, let's collapse Section 1 code by clicking on the arrow next to line number 12. This is not necessary, just aesthetically organized.

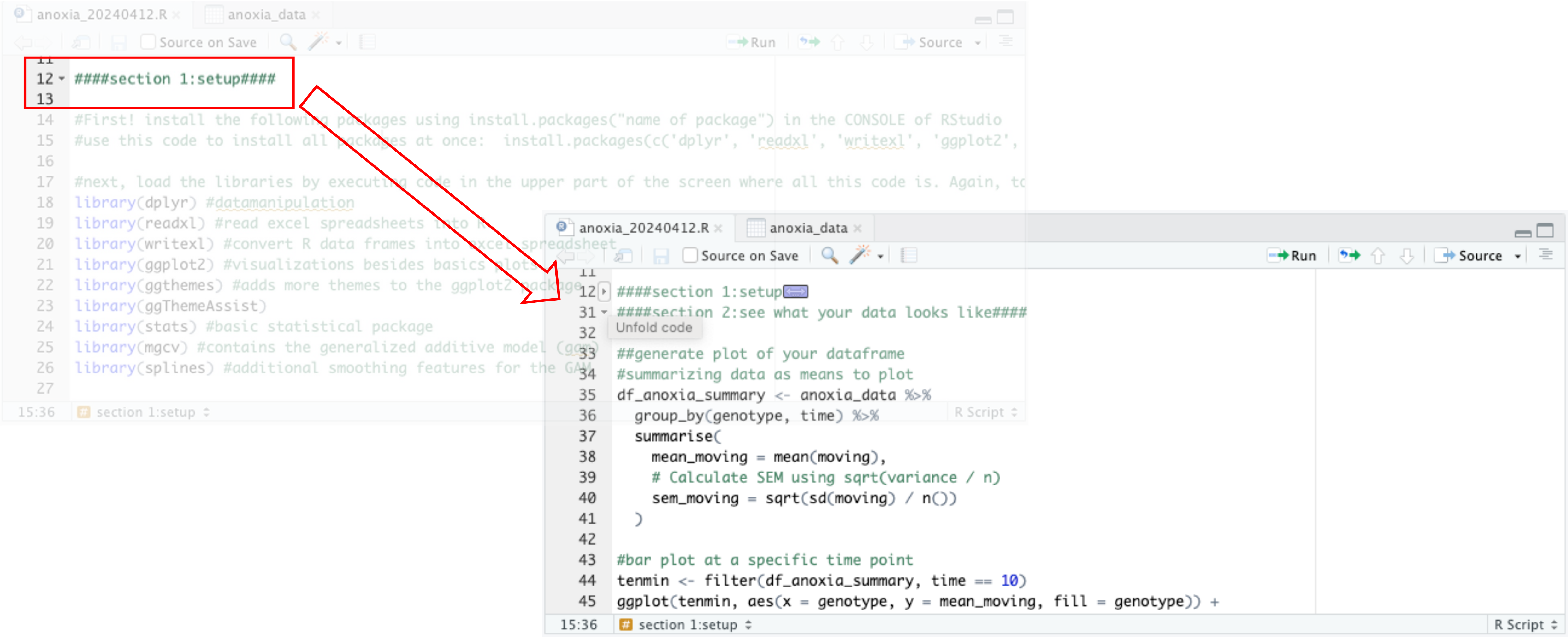

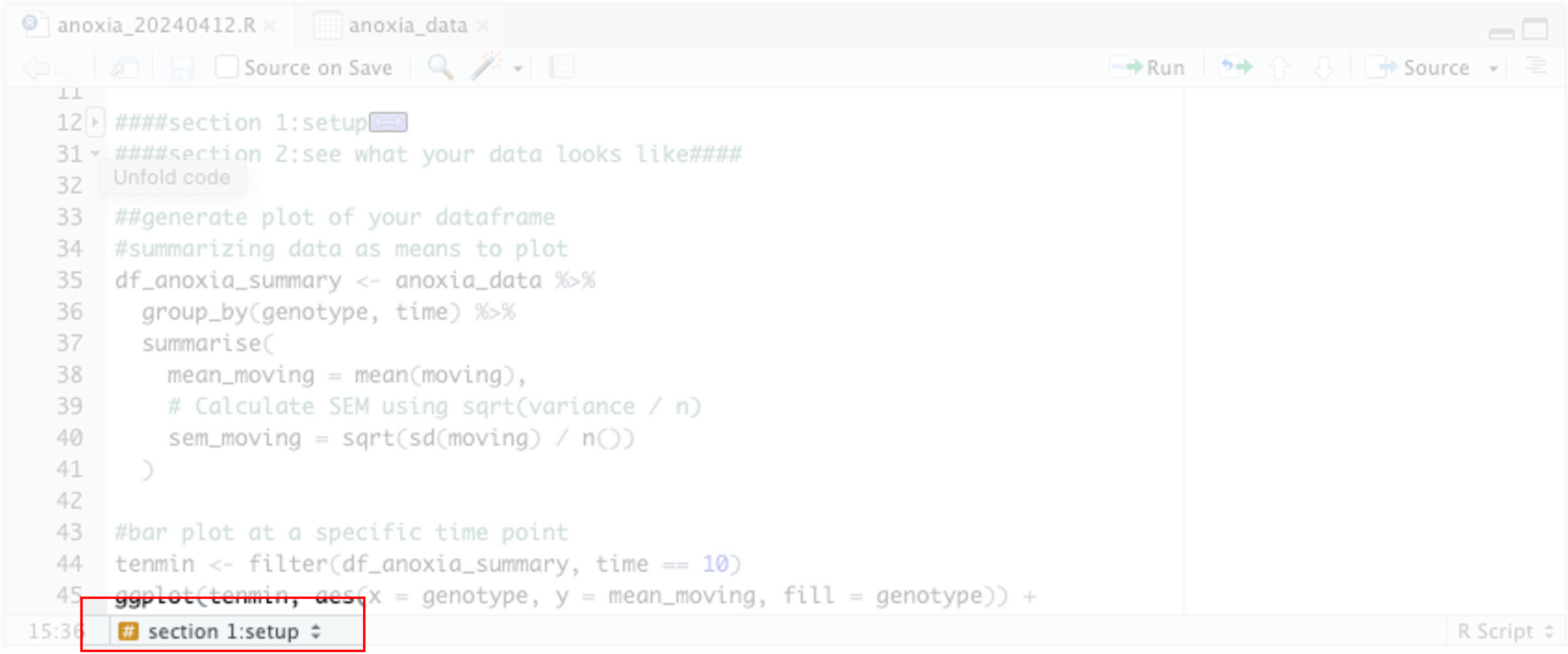

You can also navigate sections by clicking through the list here

Section 2: Data Visualization

- lines 35-41:
- With these lines of code, we create a new object called **df_anoxia_summary**

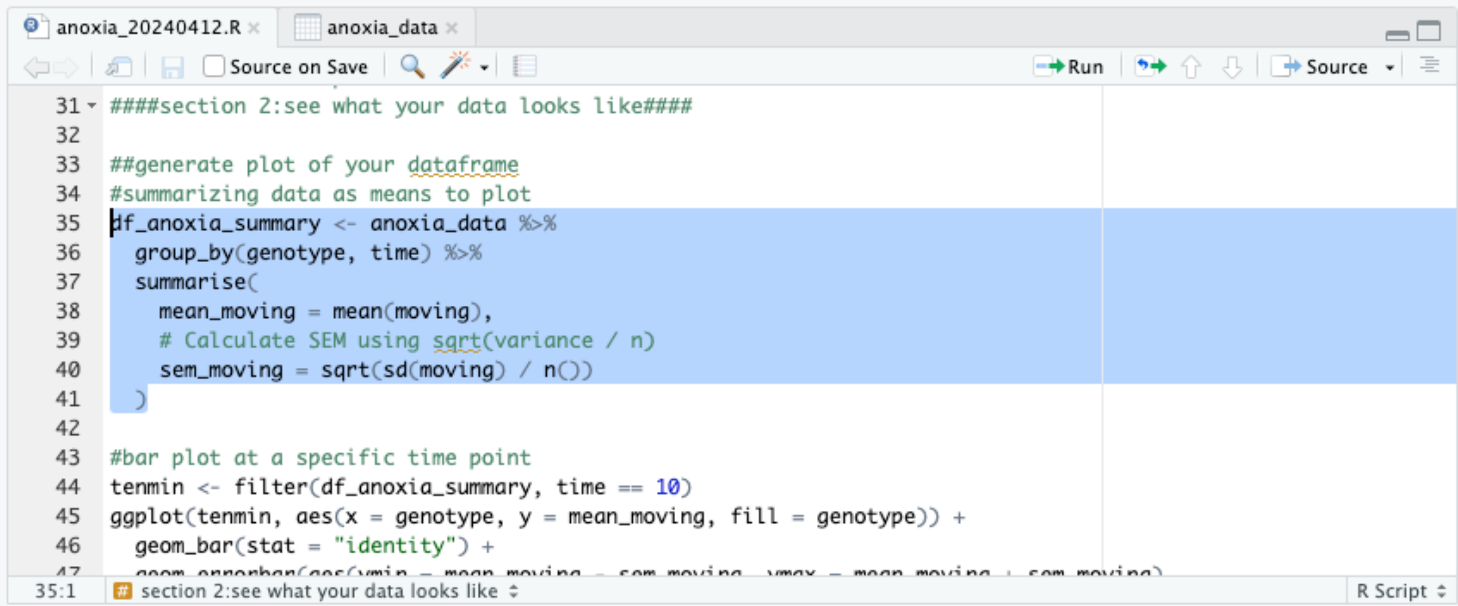

This code (highlighted in the screenshot) tells R to go into the **anoxia_data** object we created in the previous section and to do several things within that data set. The pipe operator (%>%) allows us to accomplish multiple tasks at once.

- The first task is to **group_by** which asks R to reorganize our data first by genotype and then by time within that genotype.
- The second task is to **summarise**, which asks R to create a mean of the moving values at each time point in the respective genotype. We also want to know the variance, in this case the standard error (SEM) of the moving values. So we give R the math equation to calculate SEM.

The new object can be viewed by clicking on it in the environment pane. We can see our data organized by genotype, time, and two new columns (mean_moving and sem_moving). It is a good habit to check new objects you create to verify that R did what you wanted it to.

anoxia_20240412.R | df_anoxia_summary | anoxia_data

Filter

	genotype	time	mean_moving	sem_moving
1	N2	5	0.2333333	0.1197474
2	N2	10	0.2258065	0.1170916
3	N2	15	0.3461538	0.1366022
4	N2	20	0.3076923	0.1345475
5	N2	25	0.3333333	0.1333867
6	N2	30	0.3809524	0.1539346
7	N2	35	0.2307692	0.1285524
8	N2	40	0.2500000	0.1254931
9	N2	45	0.3225806	0.1238093
10	N2	50	0.2400000	0.1320439
11	N2	55	0.3103448	0.1274178

Showing 1 to 11 of 36 entries, 4 total columns

Now that we have our data organized, let's try to visualize it as a simple bar graph. This particular data set has many time points per genotype, so viewing them all could become clunky. Let's view only a critical time point, 10 minutes after oxygen re-exposure.

- lines 43-53:
- With these lines of code, we first create a new object called **tenmin** that is a data sheet only containing the mean and SEM of the 10 minute time point for each genotype. Again, open the **tenmin** object in the environment and view it to double check your data.

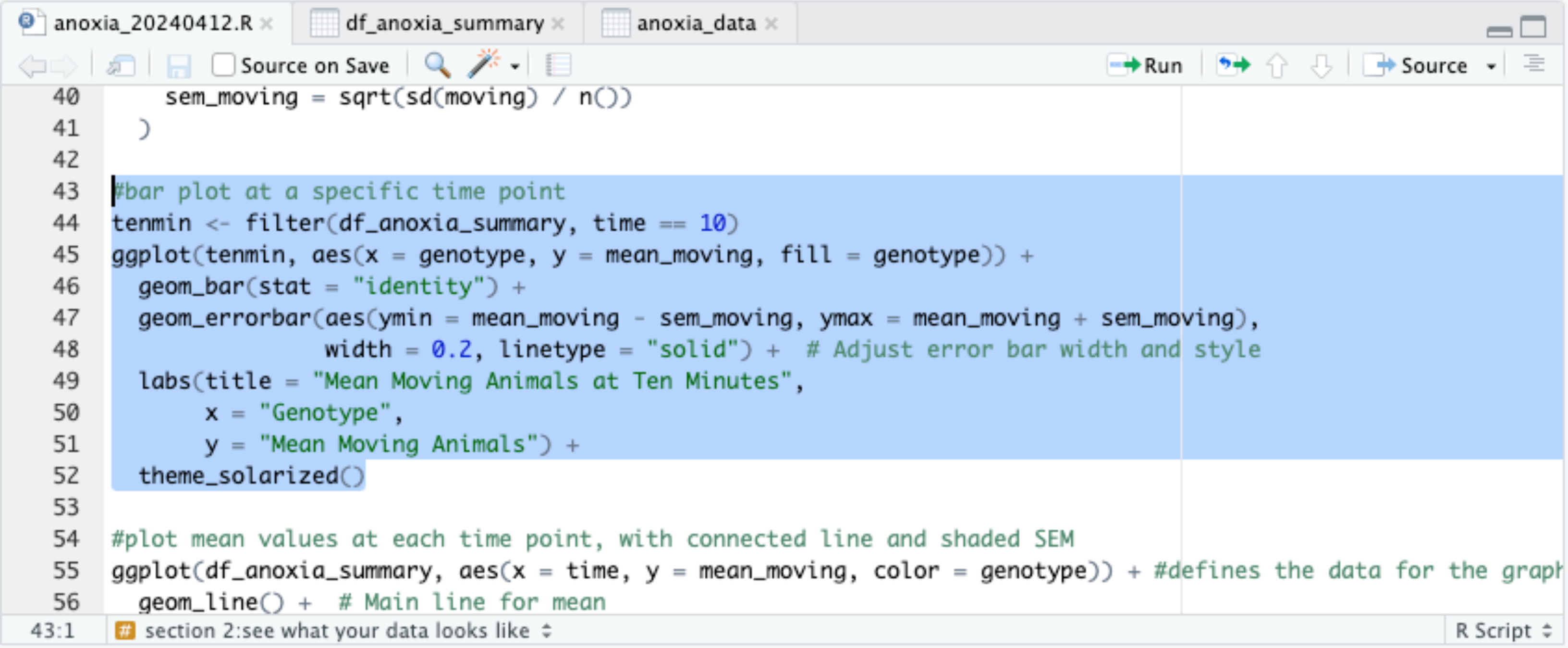


```r
40     sem_moving = sqrt(sd(moving) / n())
41   )
42
43 #bar plot at a specific time point
44 tenmin <- filter(df_anoxia_summary, time == 10)
45 ggplot(tenmin, aes(x = genotype, y = mean_moving, fill = genotype)) +
46   geom_bar(stat = "identity") +
47   geom_errorbar(aes(ymin = mean_moving - sem_moving, ymax = mean_moving + sem_moving),
48                 width = 0.2, linetype = "solid") +  # Adjust error bar width and style
49   labs(title = "Mean Moving Animals at Ten Minutes",
50        x = "Genotype",
51        y = "Mean Moving Animals") +
52   theme_solarized()
53
54 #plot mean values at each time point, with connected line and shaded SEM
55 ggplot(df_anoxia_summary, aes(x = time, y = mean_moving, color = genotype)) + #defines the data for the graph
56   geom_line() +  # Main line for mean
```

We then call on the ggplot function to take the tenmin object and plot it as a bar plot (geom_bar). ggplot (actually, ggplot2 is the current version) has many different visualization options. This is not the only way to create graphs within R. But it is a very versatile option; we can customize axes names, chart line widths, and use a vast variety of themes to give our charts a nice look.

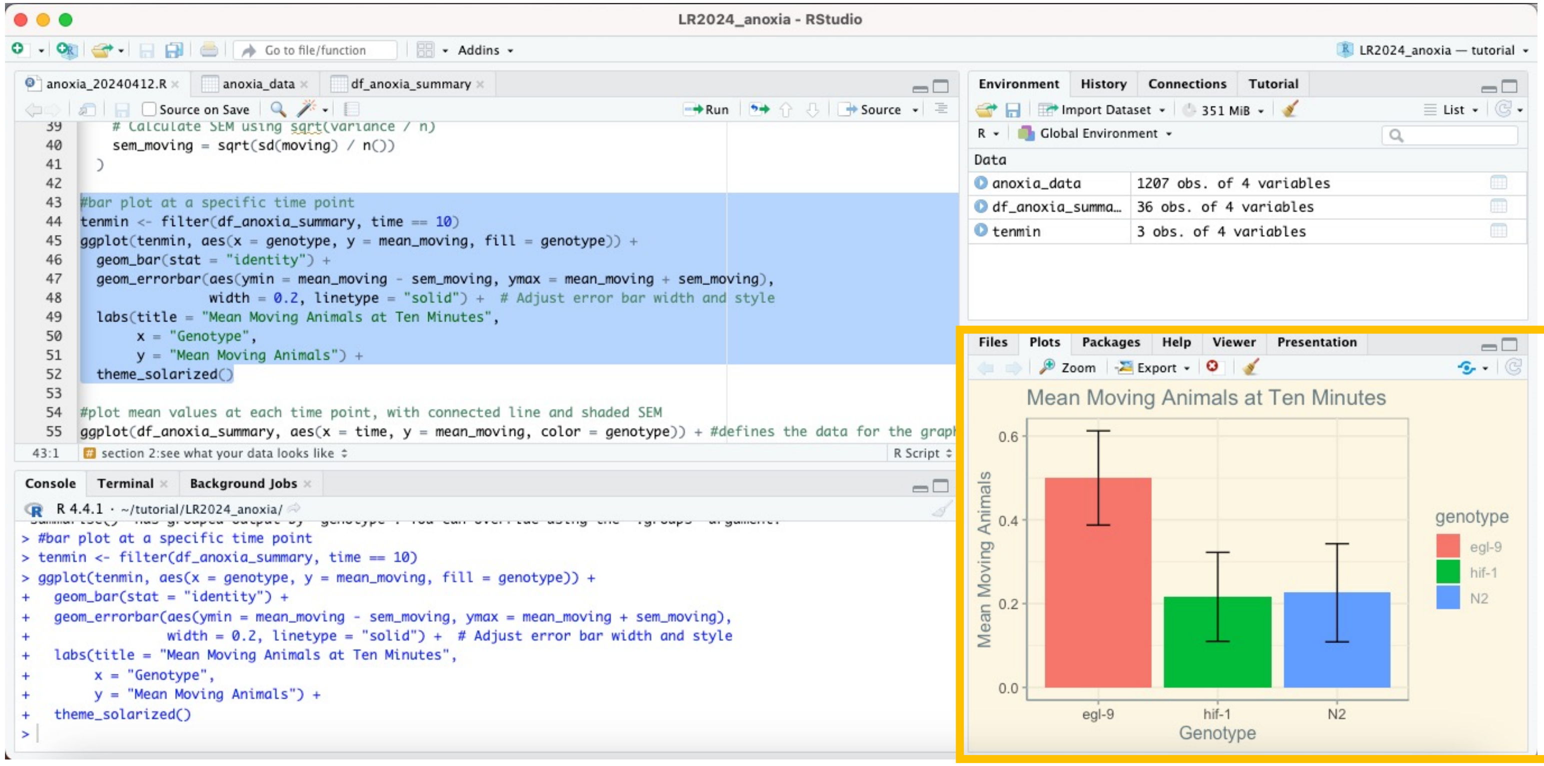

Please do browse more information on ggplot2. We won't go into what each line of code here means, but this information is easily accessible.

Notice the chart can be viewed in the "plots" tab of the Output section

One basic point in the code is you can continue to add customizations to the graph by adding a plus sign and then the desired lines of code. This is notable as we previously strung together code with the pipe operator (%>%)

```
43  #bar plot at a specific time point
44  tenmin <- filter(df_anoxia_summary, time == 10)
45  ggplot(tenmin, aes(x = genotype, y = mean_moving, fill = genotype)) +
46    geom_bar(stat = "identity") +
47    geom_errorbar(aes(ymin = mean_moving - sem_moving, ymax = mean_moving + sem_moving),
48                  width = 0.2, linetype = "solid") +  # Adjust error bar width and style
49    labs(title = "Mean Moving Animals at Ten Minutes",
50         x = "Genotype",
51         y = "Mean Moving Animals") +
52    theme_solarized()
```

In this example:

- we have specified the dataset to use for the graph (line 45)
- the type of graph (line 46)
- added error bars and customized them (lines 47-48)
- added names to the graph and axes (lines 49-51)
- stylized everything according to a theme (line 52).

Now let's move on to the more complex example of how to create a graph. This time, I want to see how the genotypes behave across the whole hour they were observed.

```
53 #plot mean values at each time point, with connected line and shaded SEM
54 ggplot(df_anoxia_summary, aes(x = time, y = mean_moving, color = genotype)) + #defines the data for the graph
55   geom_line() +  # Main line for mean
56  # scale_color_manual(values = primary_colors) +  # Map primary colors directly, can delete this line if no color scheme defined
57   geom_ribbon(aes(ymin = mean_moving - sem_moving,
58                   ymax = mean_moving + sem_moving),
59               fill = "cyan", alpha = 0.2, linetype = "dashed") + #this is the shaded standard deviation
60   theme_solarized() + #theme of choice, makes the background beige and the text light grey
61   labs(title = "Mean anoxia recovery",
62        x = "time (min)",
63        y = "number of animals moving",
64        color = "Genotype") +
65   # Increase the number of breaks for the x-axis
66   scale_x_continuous(breaks = seq(min(0), max(60), length.out = 5))
```

- lines 54-68: With these lines of code, we create another kind of visualization.
- For this plot, we have used the geom_line plot within ggplot2. We have customized the SEM ribbon around the main line to be transparent cyan (line 60) the axes to reflect the fact that we are viewing data scaled to 100 (line 68) and distance between numbers on the x axis (line 67). [Here is another resource] for customizations and more information.
- Note that I have multiplied the "mean" values by 100 so that the y axis shows the % values and not the ratios.
- Our new plot

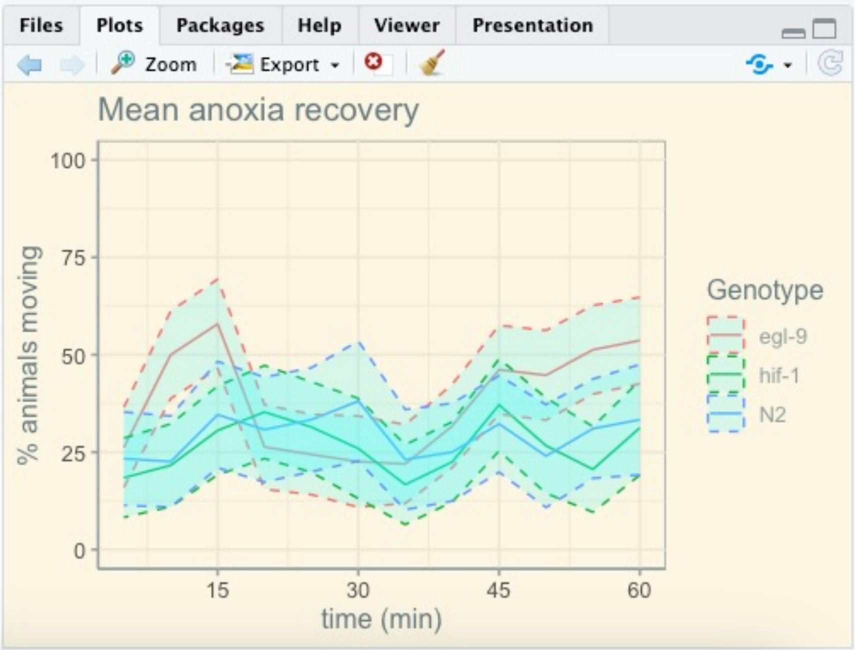

Section 3: Statistical Analysis

Simple example: When we visualize our data, we can see certain trends arise amongst the genotypes. Going back to our bar graph of the ten minute time point, let’s see if the means vary significantly.

- lines 72-77:
- With these lines of code, we are conducting a one way ANOVA (line 74) on a filtered data set at 10 min (line 73), with a Tukey’s post-hoc multiple comparisons test (line 75). We then create a summary of the ANOVA (**tenminaov**).

```
72  #one-way anova at a single time point
73  tenmin2 <- filter(anoxia_data, time == 10)
74  tenminaov <- aov(moving ~ genotype, data = tenmin2)
75  TukeyHSD(tenminaov)
76  summary(tenminaov)
77  print(tenminaov)
```

We can look in the console of the RStudio window for the results of our statistical tests.

- Based on the Tukey's post-hoc comparison, the *egl-9(sa307)* strain is significantly different from both of the other strains tested in this experiment.
- We also see with the summary of the ANOVA that the genotype feature contains significant differences. And we can see the other information used to conduct the test (sum of squares, df, etc.)

```
> TukeyHSD(tenminaov)
  Tukey multiple comparisons of means
    95% family-wise confidence level

Fit: aov(formula = moving ~ genotype, data = tenmin2)

$genotype
                    diff        lwr         upr     p adj
hif-1-egl-9 -0.283783784 -0.5302453 -0.03732227 0.0197461
N2-egl-9    -0.274193548 -0.5327485 -0.01563855 0.0349573
N2-hif-1     0.009590235 -0.2535016  0.27268206 0.9958682
> summary(tenminaov)
             Df Sum Sq Mean Sq F value Pr(>F)
genotype      2  1.968  0.9839   4.763 0.0105 *
Residuals   105 21.690  0.2066
---
Signif. codes:  0 '***' 0.001 '**' 0.01 '*' 0.05 '.' 0.1 ' ' 1
> print(tenminaov)
Call:
   aov(formula = moving ~ genotype, data = tenmin2)

Terms:
                genotype Residuals
Sum of Squares  1.967782 21.689625
Deg. of Freedom        2       105

Residual standard error: 0.4544974
Estimated effects may be unbalanced
```

Complex example: As argued in our text, the most appropriate test to compare our time series behavioral data is a logistic regression.

```
84  ##simple logistic regression
85  an_glm <- glm(moving ~ genotype_unordered, family = binomial(link = "logit"), # the default is logit
86                data = anoxia_data)
87  summary(an_glm)
88  plot(an_glm)
```

- With these lines of code, we are conducting a logistic regression using a generalized linear mode (lines 85-86).
    - Notice that we are telling the model to consider the differences within the moving values amongst the genotypes.
- We can view the results of the test by asking for a summary of the object we created **an_glm** (line 87) and ask R to plot the logistic for us (line 88)

You may have noticed that our genotype group in the GLM was called **genotype_unordered**. This was a little trick to make sure that the model knew which genotype to compare the others to, in our case this I want to compare everything to (the reference) what wild type ("N2") was doing in these conditions.

```
79 # Define genotype as a factor and make N2 (our wild type strain) your reference sequenced
80 genotype_unordered <- factor(anoxia_data$genotype, ordered = FALSE) #makes genotype a factor
81 genotype_unordered <- relevel(genotype_unordered, ref = "N2") #makes N2 within genotype the reference
82 #genotype
```

- We first define genotype as a factor- this is somewhat unnecessary since this is clearly a categorical variable, but sometimes it is better to make sure the model won't make assumptions (line 80). We next tell R that the reference strain is "N2" (line 81).
- If we look in environment, we can see that genotype_unordered has been defined as a value. R knows that this is not an object on its own, rather that it is a value within another object **(anoxia_data).**

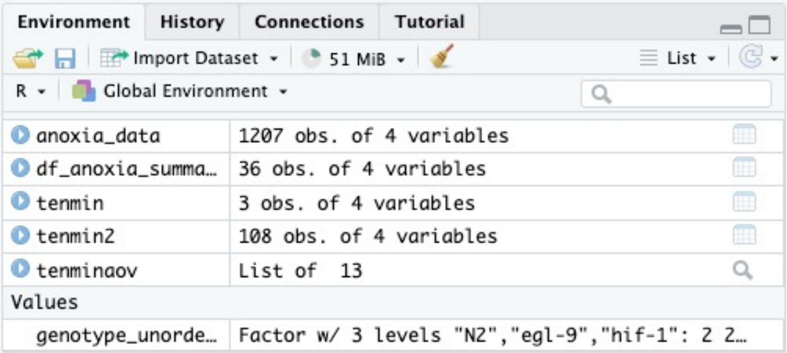

Let’s again look in the console to see the results of the test. Here is another resource that accessibly goes over the results of the logistic GLM in more detail.

```
> ##simple logistic regression
> an_glm <- glm(moving ~ genotype_unordered, family = binomial(link = "logit"),
+               data = anoxia_data)
> summary(an_glm)

Call:
glm(formula = moving ~ genotype_unordered, family = binomial(link = "logit"),
    data = anoxia_data)

Coefficients:
                        Estimate Std. Error z value Pr(>|z|)
(Intercept)              -0.8948     0.1224  -7.309 2.69e-13 ***
genotype_unorderedegl-9   0.4123     0.1550   2.660  0.00781 **
genotype_unorderedhif-1  -0.1342     0.1656  -0.810  0.41792
---
Signif. codes:  0 ‘***’ 0.001 ‘**’ 0.01 ‘*’ 0.05 ‘.’ 0.1 ‘ ’ 1

(Dispersion parameter for binomial family taken to be 1)

    Null deviance: 1506.8  on 1206  degrees of freedom
Residual deviance: 1491.2  on 1204  degrees of freedom
AIC: 1497.2

Number of Fisher Scoring iterations: 4
```

Notice the column called “Estimate Std,” these values are also known as the coefficients. The intercept is the log odds of moving for moving for the reference group. We can take the exponent of the values here to give us an odds ratio. And we can do this all within R! Just write in the console:
>exp(0.4123)
This is the coefficient of egl-9 versus N2. The result:
[1] 1.510287
Tells us that when compared to wild type, *egl-9* has a 1.5 positive odds of moving. You can see for *hif-1*, there is a negative coefficient, so this means that this strain was observed as moving less across the time points than N2. Yet, this was not significant, as we can see from the Pr. column. This P value is calculated from the z value and the ratio of the Std. Error.

The other items reported here don't really apply to our rather simple GLM. But we include some very basic definitions here.

```
> ##simple logistic regression
> an_glm <- glm(moving ~ genotype_unordered, family = binomial(link = "logit"),
+               data = anoxia_data)
> summary(an_glm)

Call:
glm(formula = moving ~ genotype_unordered, family = binomial(link = "logit"),
    data = anoxia_data)

Coefficients:
                        Estimate Std. Error z value Pr(>|z|)
(Intercept)              -0.8948     0.1224  -7.309 2.69e-13 ***
genotype_unorderedegl-9   0.4123     0.1550   2.660  0.00781 **
genotype_unorderedhif-1  -0.1342     0.1656  -0.810  0.41792
---
Signif. codes:  0 '***' 0.001 '**' 0.01 '*' 0.05 '.' 0.1 ' ' 1

(Dispersion parameter for binomial family taken to be 1)

    Null deviance: 1506.8  on 1206  degrees of freedom
Residual deviance: 1491.2  on 1204  degrees of freedom
AIC: 1497.2

Number of Fisher Scoring iterations: 4
```

The dispersion parameter captures variability of the binomial, it is very unusual for this to be taken as anything other than 1. This is not typically something you will look at, as it is not telling you variability of the mean but rather of the distribution.

The AIC is only used to compare models- if we were fitting a bunch of models, which we are not in this example. This metric is mostly outdated, but still reported by default.

The null and residual deviance is used if you are comparing different models- not normally done.

Finally, R reports on how many steps did it took to iterate through the optimization algorithm - if it didn't converge it would tell you.

R has a built-in way to plot the results of the GLM; they are used for troubleshooting your model. The interpretation of these graphs goes beyond the scope of the statistical expertise of this project (i.e. please consult your resident biostatistician ☺); but we provide basic definitions here.

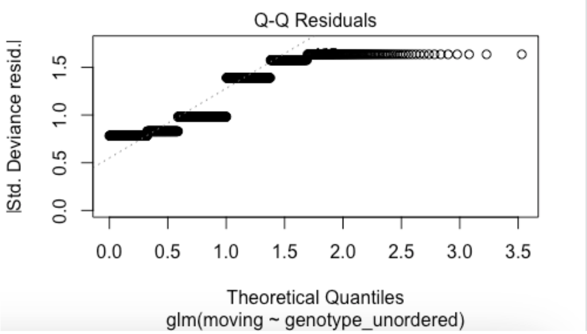

quantile-quantile residuals plot
tells you if your residuals are normally distributed in a linear fashion.

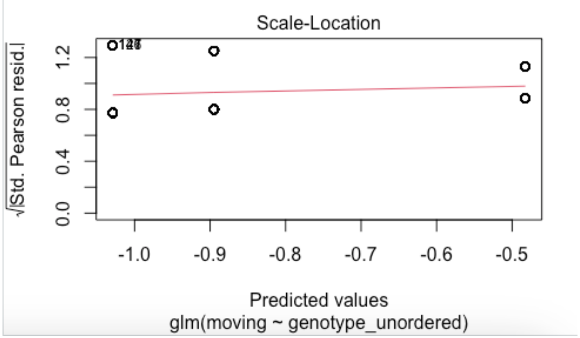

Helps determine heteroscedasticity, a condition when your variance of errors is not constant across observations.

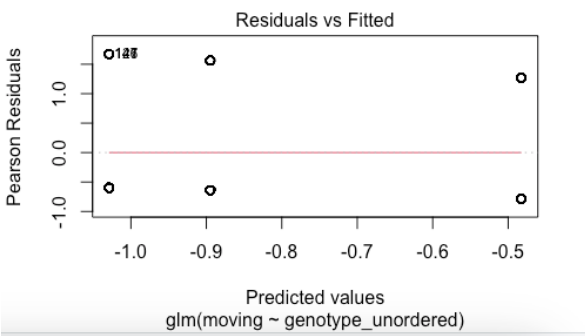

this plot shows you if there are trends in your residuals.

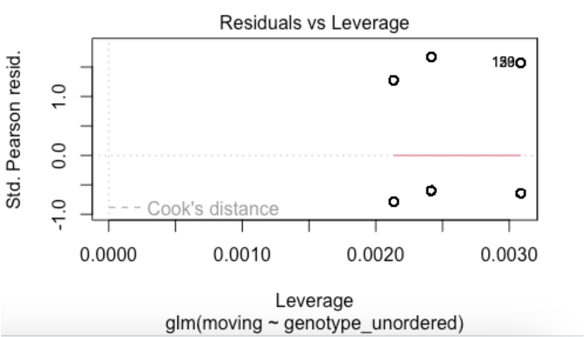

can help identify outliers.

These plots are generally not useful in logistic regression models!

Finally, let’s apply a more complex model to the time series behavioral data: the generalized additive model (GAM). This regression model is useful for data that contains non linear relationships. In our data, it is quite clear that there is a general pattern of behavior, but I really want to be able to define that pattern. A GAM can also help with this.

```
#general additive model
an_gam <- gam(moving ~ genotype_unordered + s(time), data = anoxia_data)
summary(an_gam)
```

- GAM is a regression model and the format of the GAM code is similar to the GLM code. We define what it is to be compared; in this case, its moving versus the behavior of genotype over time.

We can ask R to plot this model. Here, there are three iterations of this.

```
90 plot(an_gam,pages=1,residuals=TRUE)  ## show partial residuals
91 plot(an_gam,pages=1,seWithMean=TRUE) ## `with intercept' CIs
92 plot(an_gam, select = 1, shade = TRUE, seWithMean = TRUE)
93 ## run some basic model checks, including checking
94 ## smoothing basis dimensions...
```

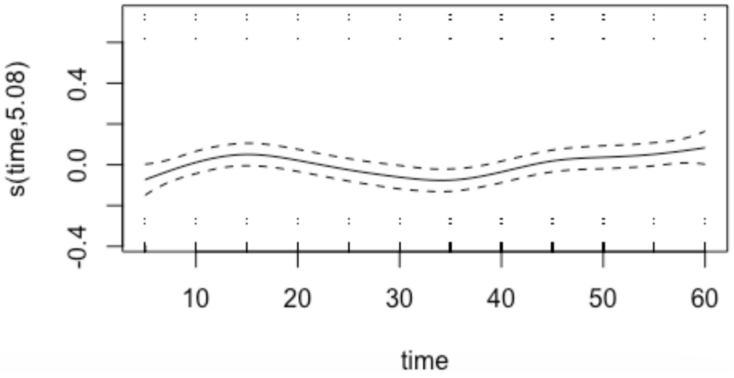

line 90: the basic graph

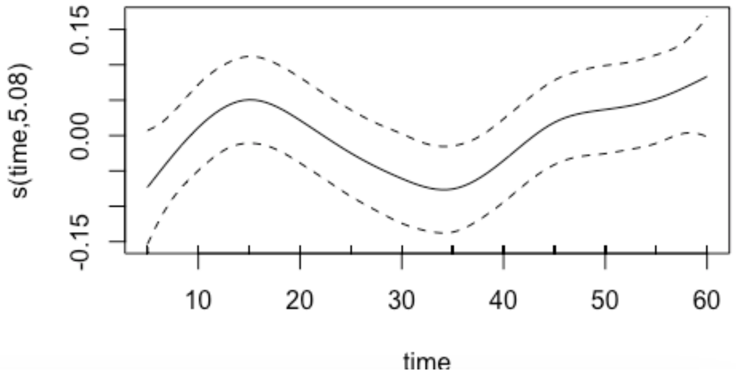

line 91: adjusted y scale

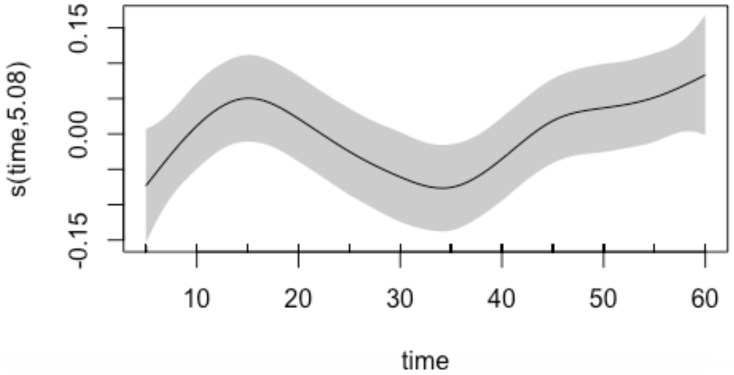

line 92: and because I like the aesthetic of it, I have shaded in the error

Well done on completing the tutorial! You are now a coder.

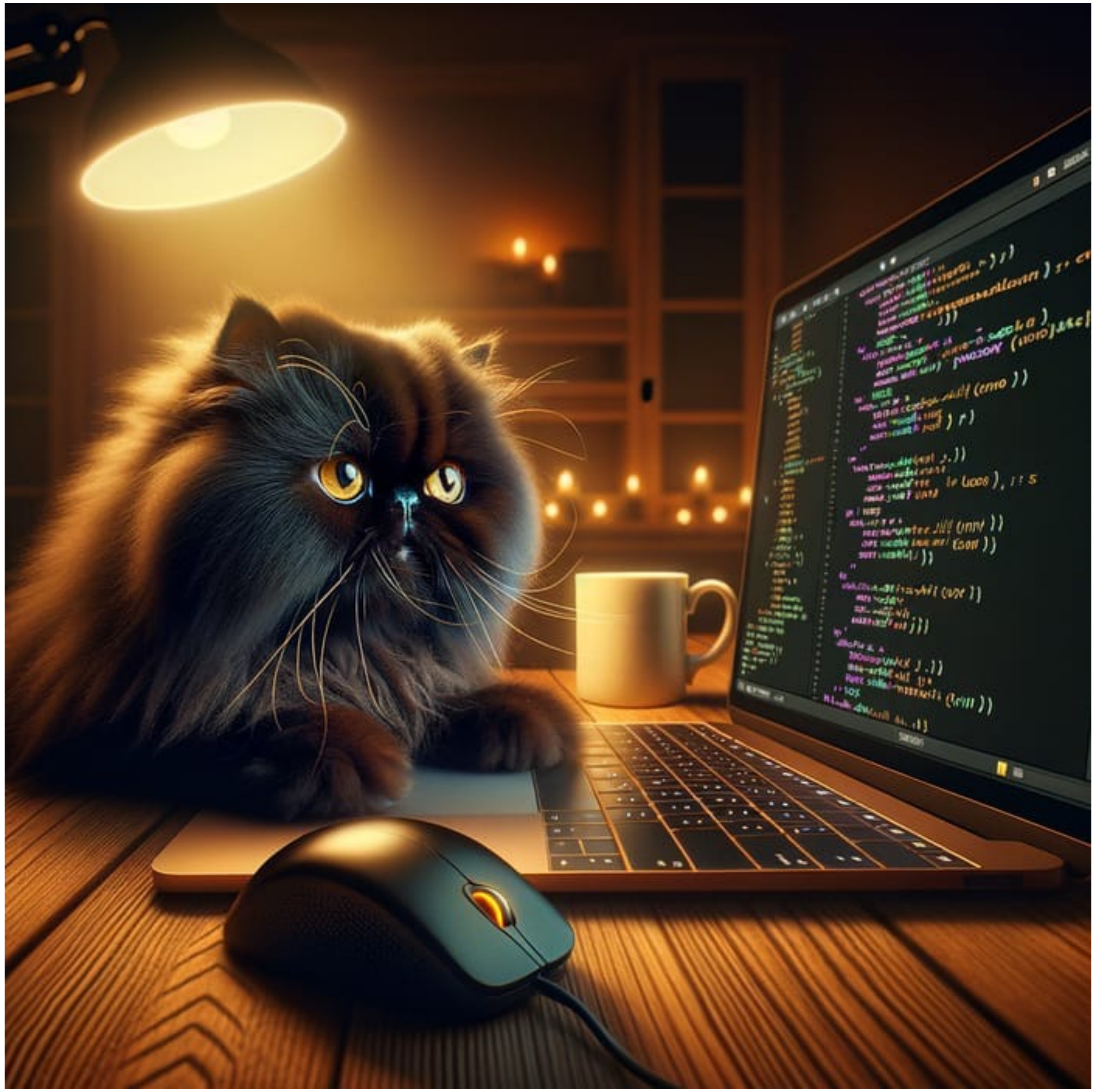

Source
